# Viral introductions and return to baseline sexual behaviors maintain low-level mpox incidence in Los Angeles

**DOI:** 10.1038/s41467-026-71993-w

**Published:** 2026-04-17

**Authors:** Miguel I. Paredes, Citina Liang, Sze-chuan Suen, Ian W. Holloway, Jacob M. Garrigues, Nicole M. Green, Trevor Bedford, Nicola F. Müller, Joseph Osmundson

**Affiliations:** 1 Vaccine and Infectious Disease Division, Fred Hutchinson Cancer Center, Seattle, WA USA; 2 Howard Hughes Medical Institute, Seattle, WA USA; 3 Daniel J. Epstein Department of Industrial and Systems Engineering, University of Southern California Viterbi School of Engineering, Los Angeles, CA USA; 4 School of Nursing, University of California Los Angeles, Los Angeles, CA USA; 5 Los Angeles County Department of Public Health, Los Angeles, CA USA; 6 Division of HIV, Infectious Diseases & Global Medicine, Department of Medicine, University of California San Francisco, San Francisco, CA USA; 7 Department of Biology, New York University, New York, NY USA

**Keywords:** Epidemiology, Pox virus, Phylogenetics

## Abstract

In 2022, mpox clade IIb disseminated around the world, causing outbreaks in more than 117 countries. Despite the decay of the 2022 epidemic and the increased immunity within sexual networks, mpox continues to persist in North America without extinction, raising concerns of future outbreaks. We combined phylodyamic inference and microsimulation modeling to understand the heterogeneous dynamics governing local mpox persistence in Los Angeles County (LAC) from 2023 to 2024. Our Bayesian phylodynamic analysis revealed a time-varying pattern of viral importations into the county, which seeded mpox outbreak clusters that display “stuttering chains” dynamics. Our phylodynamics-informed microsimulation model demonstrated that the mpox cases in LAC can be explained by a combination of waves of viral introductions, a median effective reproductive rate below one, and a return to near-baseline sexual behaviors after the 2022 epidemic. Our counterfactual scenario modeling showed that frequent public health interventions that either promote increased isolation of infectious individuals or enact behavior-modifying campaigns during the periods with the highest viral importation intensity are actionable and effective at curbing mpox cases. Our work highlights the factors that maintain present-day mpox dynamics in a large, urban US county and describes how to leverage these results into community-centered public health interventions.

## Abstract (web summary)

Mpox persists in North America without extinction, raising concerns of future outbreaks. Authors combined phylodynamics and microsimulation to show how viral introductions and rebounding sexual behaviors promote mpox persistence in Los Angeles.

## Introduction

Mpox is a viral infection caused by the monkeypox virus (MPXV), an orthopoxvirus closely related to smallpox^1^. In 2022, mpox spread globally, largely via sexual networks, causing tens of thousands of cases^2^. Mpox clade IIb was introduced into humans around 2014 in Nigeria^3^ and was the causative genetic clade of the 2022 outbreak. Mpox clade IIb continues to spread around the world, including in the United States (US)^4–6^. Additionally, a current outbreak of clade I in Central Africa has also spread internationally^7,8^.

In 2022, clade IIb mpox cases in the US reached over one hundred per day. Mpox infections in the US have since remained at low, but persistent, levels^9,10^. While sporadic larger mpox clusters have occurred, they have neither grown into a large-scale epidemic nor been eradicated, as would be expected if the effective reproduction number (*Rt*) was above or below one, respectively^11^. The mechanisms maintaining sporadic mpox incidence locally could include viral introductions via travel^12^, small local clusters where the effective reproductive number *Rt* is larger than one (e.g., heavy-tailed infection dynamics)^13^, a combination of these factors, or other undescribed mechanisms. If either travel or limited local clusters cause a majority of mpox transmission in a specific geographical location, targeted public health interventions that respond to the dynamics of the epidemic could potentially prevent a large proportion of mpox cases.

Disentangling the contribution of travel-related and local transmission on infectious disease dynamics is difficult from case counts alone. Alternatively, phylodynamics enables the tracking of viral movement across time and space via analysis of viral genomes^14^. Prior work has employed phylodynamics to understand global, regional, and local mpox spread by leveraging global sequencing efforts to examine mpox transmission prior to widespread testing availability and to understand the interplay between viral introductions and local spread^6,15,16^. Phylodynamics works in a retrospective fashion to model viral evolution and transmission.

Microsimulation models simulate individual-level disease trajectories by combining mechanistic descriptions of disease processes with empirical data and parameters, both measured and estimated, to determine the dynamics underlying infectious disease transmission^17^. By simulating counterfactual scenarios, microsimulations can elucidate the mechanistic factors that determine and curb spread. Prior microsimulation work on mpox dynamics has been used at a local level to both understand factors that promoted the decline of the 2022 epidemic and to test the effectiveness of public health interventions^18^. Microsimulation models, however, are often limited by data availability and model assumptions.

To address these shortcomings, we combine phylodynamics and microsimulation modeling to understand mpox spread in Los Angeles County (LAC) in 2023 and 2024. We employ Bayesian phylodynamics to estimate mpox importation dynamics into LAC, and use our phylodynamic results to parameterize a microsimulation model of mpox with a force of viral importations. Through our combined approach, we estimate the role of various factors in promoting mpox persistence in 2023–2024, such as the return of baseline sexual behaviors, rates of isolation for those with diagnosed mpox, and the role of importation versus local mpox transmission. We then use our mpox microsimulation model to evaluate the public health impact of interventions that target the identified dynamics of local spread. The combination of these two computational methods serves to answer complex and essential questions regarding the largely understudied current state of the mpox epidemic and provides direct opportunities for public health action.

## Results

### Low-level mpox incidence predominates in Los Angeles County (LAC) following the 2022 epidemic

**Fig. 1:**
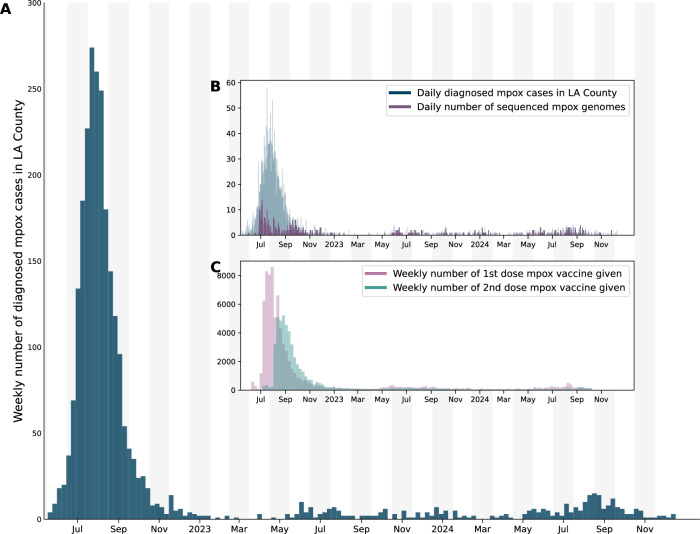
Mpox epidemiology in Los Angeles County (LAC). **A** The figure shows the weekly number of diagnosed mpox cases in LAC from June 2022 through December 2024. **B** The daily number of diagnosed mpox cases (blue) with the daily number of mpox sequences collected in LAC overlaid (purple) from June 2022 to December 2024. **C** The weekly number of mpox vaccinations that were administered in Los Angeles County from June 2022 to October 2024, divided between the number of first doses (pink) and second doses (teal) given. LA Los Angeles. Source data are provided as a Source data file.

After mpox was initially detected in May 2022, the number of diagnosed mpox cases in LAC grew sharply, peaking in mid-August 2022 (Fig. 1A). By November of that year, cases had dropped rapidly, with only 31 cases being reported that month compared to 1033 in August. Since the start of 2023, mpox cases in LAC have been sporadic, mostly characterized by periods of low incidence followed by small clusters of infections usually found from May-July or December-January (Fig. 1A)^19^. Similar patterns can be seen in the number of third-generation mpox vaccinations administered, whereby the majority of first and second doses were given in the summer and fall of 2022, followed by small increases in 2023 and 2024 surrounding early summer (Fig. 1C).

To better understand the complex dynamics of mpox in LAC after 2022, we deployed a phylodynamic approach wherein the genome sequence of a viral infection can differentiate between local spread and introductions from other locations. Without genetic analysis or extensive contact tracing, these modes of transmission are difficult to differentiate with traditional public health surveillance.

### Periods of high viral introduction promote heavy-tailed transmission clusters that maintain low-level mpox incidence

We leveraged pathogen genomes to estimate the lower bound of the number of introductions and evaluate the duration of local infection clusters. Since the start of the epidemic, the Los Angeles County Department of Public Health has sequenced a high volume of confirmed cases, leading to the number of sequences collected increasing as more cases were detected (Fig. 1B). While a low percentage of estimated cases were sequenced at the beginning of the 2022 epidemic, the majority of the months in 2023–2024 had more than 50% of the estimated mpox cases sequenced, allowing for local-scale phylodynamic investigation into ongoing local mpox transmission (Fig. S1).

**Fig. 2:**
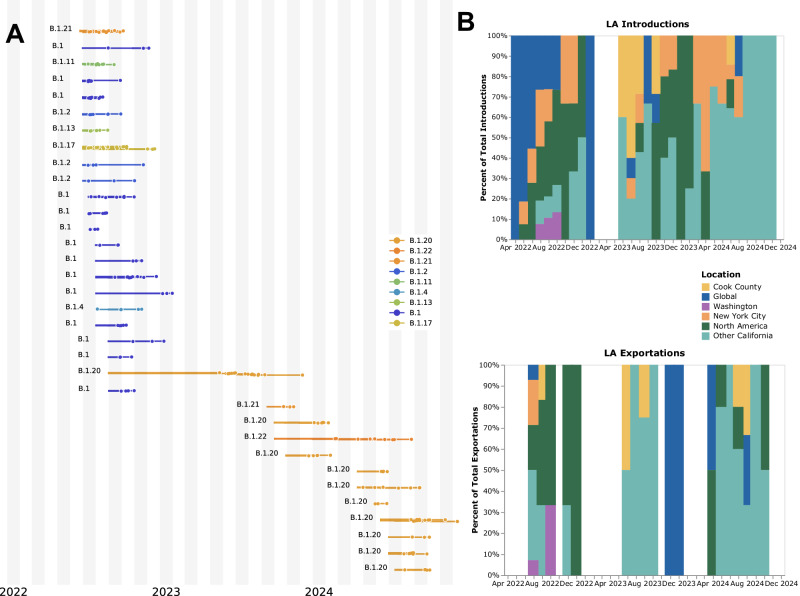
Genomic diversity, source of introduction, and location of exportation of mpox clusters in Los Angeles County (LAC). We analyzed 7859 publicly available mpox clade IIb genomes from around the world via maximum likelihood phylogenetics using Nextstrain. **A** Here, we show an exploded tree view of the maximum likelihood phylogeny that only includes the local outbreak clusters inferred to be in LAC via ancestral trait reconstruction (using Nextstrain’s augur traits functionality). Only clusters with more than three sequences are shown for clarity. The colors represent the assigned lineage of each cluster, showing the changes in mpox lineages circulating over time. **B** The plots on the right represent the inferred source of these imported clusters (*top*) as well as the location of viral exportations from LAC (*bottom*). The colors are shared between the two graphs and were constructed to focus on large metropolitan US cities and areas that have the highest level of mpox sequencing effort. The exportations and importations per month are normalized to 100% to highlight relative changes over time. LA Los Angeles.

To investigate transmission dynamics into LAC, we analyzed 497 mpox genomes sampled in LAC through December 12, 2024, alongside 7362 contextual sequences from around the world by creating a time-resolved phylogeny using Nextstrain (Fig. S2, can be viewed interactively at https://nextstrain.org/groups/blab/mpox/allcladeIIseqs)^20^. We also analyzed the inferred ancestral locations over time (Fig. 2) via discrete trait analysis, focusing on the sequences from LAC (See “Methods”). The majority of LAC clusters in 2023–2024 were found to be part of lineage B.1.20 with one outbreak cluster consisting of lineage B.1.22 (Fig. 2A). While a large part of introductions into LAC in 2022 was inferred to come from global regions outside of North America, we found that in 2023–2024 introductions from within North America, primarily New York City and other parts of California, dominate (Fig. 2B). By inferring the location of viral exports from LAC, we found that, of the sequenced areas and viruses, about half of the viral exports from 2023 to 2024 were to other California regions, while the other half were mostly into Cook County, Illinois, and New York City.

**Fig. 3:**
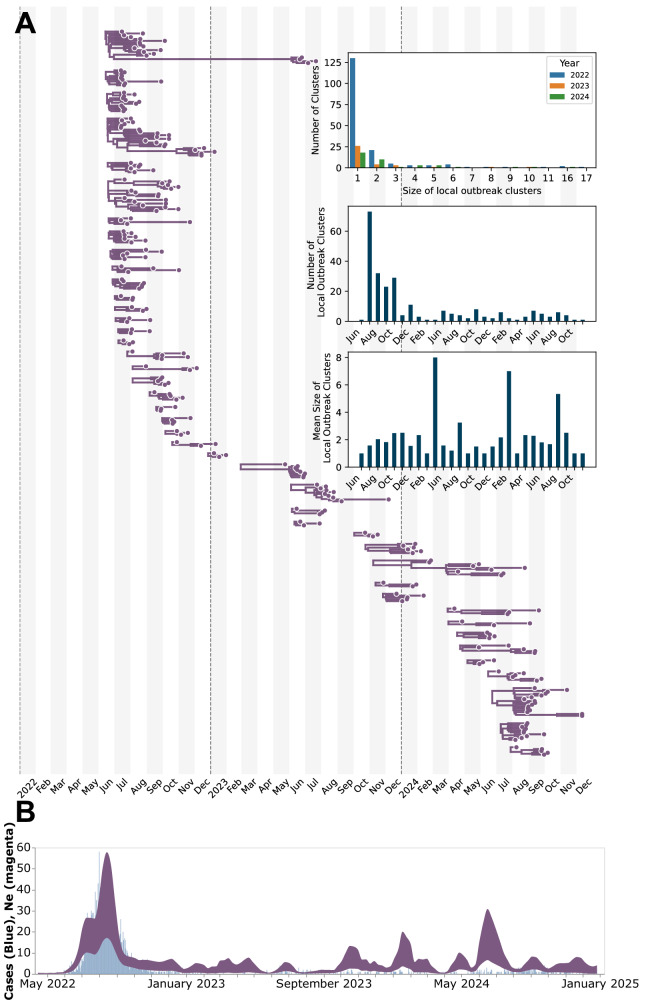
Local Los Angeles County (LAC) dynamics estimated via Bayesian phylodynamics. **A** Maximum clade credibility (MCC) summary tree from local outbreak clusters of 497 sequences showing clusters with more than two sequences. *Top inset* represents the size distribution of the identified outbreak clusters by year; the *middle inset* is the number of identified outbreak clusters by month; and the *bottom inset* represents the mean size of local outbreak clusters over time. The month is determined by the date of the earliest sequence in each cluster. **B** Estimates of effective population sizes (Neτ in years) from May 2022 through December 2024 (dark purple with bands representing the 50% HPD interval) plotted on top of the weekly number of diagnosed mpox cases (light blue). The coalescent time scale depends on both effective population size Ne (number of effective individuals) and on generation time τ (years per generation), resulting in Neτ being a measure of the coalescent time scale in years. HPD highest posterior density. Source data for the insets are provided as a Source data file.

We then split the sequences into local outbreak clusters using parsimony-based clustering to identify groups of sequences whose ancestral states were inferred to be in LAC (see “Methods”, Fig. 3A). In total, we identified 287 clusters, with the majority of them being of size 1 (*n *= 131). The distribution of identified outbreak cluster sizes is right-skewed, similar to the heavy-tailed sexual network distribution that was characteristic of the 2022 mpox epidemic, where the majority of introductions resulted in no secondary infections and a small number of introductions resulted in a large number of secondary cases (Fig. 3A top inset)^13^. While we expected the total number of clusters identified to be affected by the sequencing both within and outside of LAC, we saw a very limited impact in our sample due to the high amount of sequencing worldwide and within LAC (Fig. S3).

We modeled the local mpox dynamics via a multi-tree coalescent phylodynamic approach conditioned on the a priori identified outbreak clusters (see “Methods”). To inform our estimates of transmission dynamics using both genomic and epidemiological data, we also developed a correlated case-based prior on the effective population size estimates using the weekly number of diagnosed cases smoothed using a 3-week moving average (see “Methods”). We validated the ability of our approach to correctly estimate our parameters of interest via simulations that both incorporated transmission heterogeneity and differential sampling schemes (See Phylodynamic simulations under “Methods”, Figs. S4 and 5).

Our case-informed phylodynamic estimates of viral effective population size (*Ne*) were able to capture the temporal trends of empirical case data better than phylodynamic models informed by sequences alone (Figs. 3B and S6). We found time periods with higher *Ne* than expected by case counts alone, such as during the winter of 2023 or summer of 2024, where our *Ne* showed an increase in viral population size while case counts remained relatively constant, suggesting underdetected transmission (Fig. 3B). We found these results to be robust to differences in substitution model specification (Fig. S6).

**Fig. 4:**
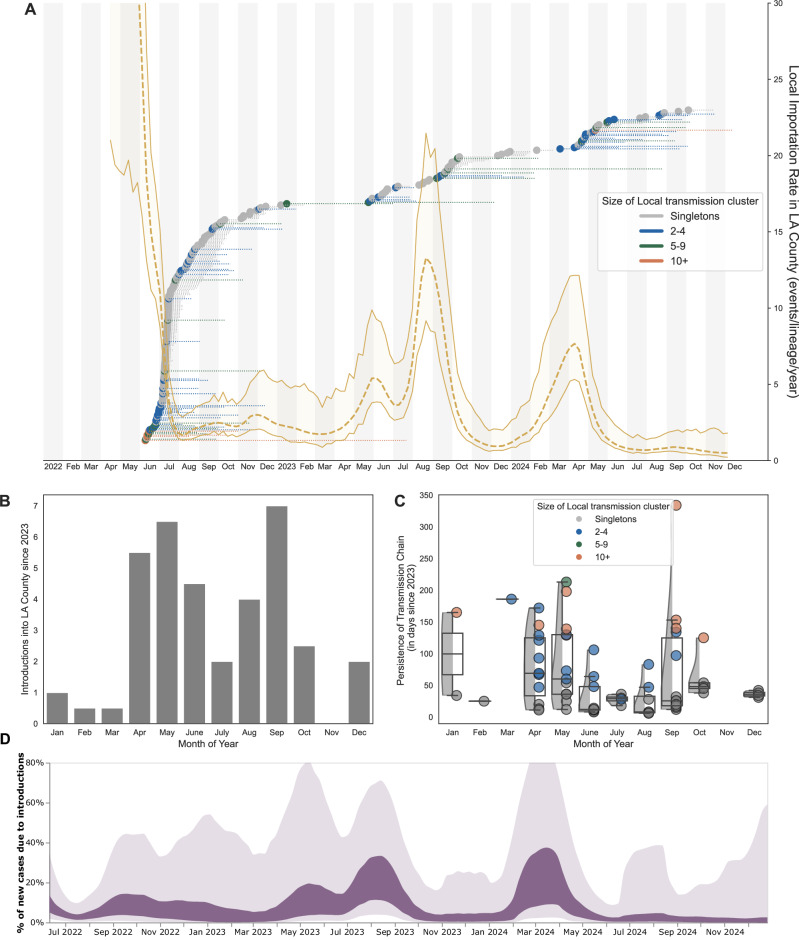
Patterns of viral introductions into Los Angeles County (LAC). **A** Here we plot the time of introduction for each local outbreak cluster estimated via our multitree coalescent approach, colored by the size of the resulting transmission cluster. The dashed line coming out of each point represents the time from the estimated date of introduction to the date of the last sequence sampled in the cluster (i.e., persistence). The yellow plot represents the time-varying rate of viral introductions estimated directly via the multitree coalescent, with the dashed line representing the median value and the upper and lower bounds representing the 95% highest posterior density (HPD). **B** Total number of viral importations into LAC per month since January 2023. The number of importations is adjusted by the number of observations in the sample. **C** The persistence times of downstream clusters by month of introduction since January 2023, with the boxplot representing the interquartile ranges, and the half violin plot representing the distribution of values. Box plots indicate the median (center line), the interquartile range (bounds of the box; 25th–75th percentiles), and whiskers extending to the minima and maxima. Scatter points are colored based on the size of the resulting transmission cluster. **D** The percentage of new cases due to introductions was estimated as the relative contribution of introductions to the overall number of infections in the region, with the dark magenta band representing the 50% HPD and the lighter magenta representing the 95% HPD. HPD highest posterior density.

Through our phylodynamic analysis, we were also able to estimate the date of importation for each identified LAC local transmission cluster, based on the most recent common ancestor time of each cluster, which provides a lower bound on the introduction time (Fig. 3A). The majority of introductions occurred during the summer of 2022, at the height of the 2022 mpox epidemic. In addition to this peak, we also saw the rate of viral introductions increase between February and June and August through October of each subsequent year (Fig. 4A–C).

While the majority of introductions resulted in singletons (one sequenced genome), we found evidence of large transmission clusters introduced in both 2023 and 2024 during those months with a high force of introduction (Fig. 4A). Given the presence of these large transmission clusters, we subsequently estimated the persistence time of each cluster (estimated as the time, in days, between the inferred date of introduction and the sampling date of the latest sequence in the cluster). The persistence times for the largest clusters showed a maintenance of low-level case counts, often extending from one peak of introductions until the subsequent increase (Fig. S7A).

We found that unique introductions on average constitute around 20–30% of estimated new cases in LAC (Fig. 4D, dark bands). In periods of low case counts, we see a wide uncertainty (Figs. 4D and S8, light bands), highlighting the potentially increased impact of introductions during those time periods. Of note, the phylodynamic estimation of the percentage of cases that are due to introductions only counts unique introductions and not secondary local spread after introduction.

The rates of introduction and the percentage of cases from introductions were highest at the times closely prior to the two larger outbreaks in 2023 and 2024 (such as in March 2024), suggesting that outbreaks elsewhere and subsequent introductions in LAC were strong contributors to these outbreaks. When we explored the relative success of importations compared to ongoing lineages over two month time periods (Fig. S9), we found further support of the high impact of introductions, especially in 2023, where introductions not only represented a large proportion of lineages in each time period (often more than 50%), but also were successful in creating downstream descendants that dominated local circulation in a short amount of time (Fig. S9, green lines).

We used our effective population size estimates to calculate *Rt*, the time-varying effective reproductive number (Fig. S10A). We combined our estimates of *Rt* together with our quantifications of the percentage of new cases due to introductions to separate out the individual contributions of introductions and local transmission on *Rt*. We found that increases in *Rt* often follow increases in the percentage of cases due to introductions. While changes in the mean infectious period used to calculate the percentage of cases that are due to introductions and Rt (see “Methods”) impacted the variability and magnitude of our results, the patterns of interplay between introductions and local transmission remain the same (Fig. S10B, C). Comparison of *Rt* estimated from our phylodynamic analysis with *Rt* from empirical case counts alone for 2023–2024 showed similar dynamics when considering the combined impact of both importations and local transmission (Fig. S11B). Removing the influence of viral importations dropped the median *Rt* estimate closer to 1 with high variability.

Additionally, given that the probability to observe a cluster of a given size is determined by the effective reproduction number *R* across a time period, transmission heterogeneity as estimated via the dispersion parameter *k*, and the fraction of infections sequenced^21,22^, we explored how t he probability to observe a cluster of size 16 (knowing we observed 64 clusters from 2023 to 2024) is impacted by *R* and *k* (Fig. S11A), assuming that 5.5% of infections were sequenced^6^. We find that for a value of *k* around 0.36, which is similar to what was estimated for previous mpox outbreaks and during the 2022 epidemic^6,23,24^, it is highly probable to observe our max cluster size of 16 even with R values as low as 0.45, suggesting that the true R could be lower than 1. We estimated the reproduction number *R* from the distribution of sequenced cluster sizes (Fig. 3A, top inset) from the same time period and found an *R* lower than one (Fig. S11B), further suggesting that the true R for the time period is lower than one and that accounting for introductions can help partially correct the overestimation of local *Rt*.

### Using phylodynamics to inform epidemiological microsimulation suggests a return to baseline sexual behavior in 2023–2024

Our previous work used microsimulation modeling to understand the impact of sexual behavior and differential vaccination strategies during the 2022 mpox outbreak in LAC^18^. That model was able to accurately capture the number of diagnosed mpox cases in LAC through the beginning of 2023 (Fig. S12A, orange line). It also showed that mpox incidence would have been expected to drop to zero by March 2023, which is contrary to the low-level incidence seen in 2023–2024 (Fig. 1A). Even if we assumed that sexual activity quickly reverted to baseline in March 2023, our model simulations still predicted quick mpox elimination (Fig. S12E). Only by simulating constant viral introductions every week into demographic strata randomly selected proportional to their population size was our model able to maintain ongoing transmission similar to the empirical number of cases (Fig. S12D), supporting the importance of introductions as seen via our phylodynamic results.

In order to simulate realistic mpox dynamics, we parameterized our microsimulation model with estimates of viral importations derived via phylodynamics. From our phylodynamic results, we estimated the absolute number of viral importations into LAC over time (Figs. 4B and S7B). Using the derived force of importations allowed us to bound the model and estimate the changes needed in sexual behavior such that the model produces outcomes overlapping with epidemiological measurements in 2023–2024.

Our microsimulation model tracked mpox dynamics by age, race/ethnicity, and HIV status, and was calibrated and validated against LAC surveillance data (See “Methods”). While mpox affected more than just MSM^25,26^, an estimated 95% of mpox cases in the US have been among MSM^27^, leading our model to be focused on this population.

Briefly, our microsimulation model included a dimensionless calibration parameter, here referred to as the Infectivity Scalar ($\alpha$), which we vary over time (See Microsimulation model, under “Methods”). The Infectivity Scalar was designed to modify the transmission impact of infected individuals on the susceptible population within their respective demographic group. Given that our model accounts for assortative mixing patterns between demographic groups as well as for the development and waning of vaccination-induced immunity, the Infectivity Scalar largely serves to capture changes in behavior throughout time, representing the relative risk of disease spread.

For the first 5 weeks following the surge of mpox in LAC in 2022 (from early July to early August), $\alpha$ was previously calibrated to 2.2, establishing a baseline for the impact of sexual behavior on mpox transmission (See^18^, visually represented in Fig. S12A). Following those first 5 weeks, $\alpha$ was lowered to 0.7 to align model outputs with empirically observed case estimates, representing a significant reduction in the risk of disease spread via changes in sexual behavior, in concordance with previously-documented reports^28,29^.

**Fig. 5:**
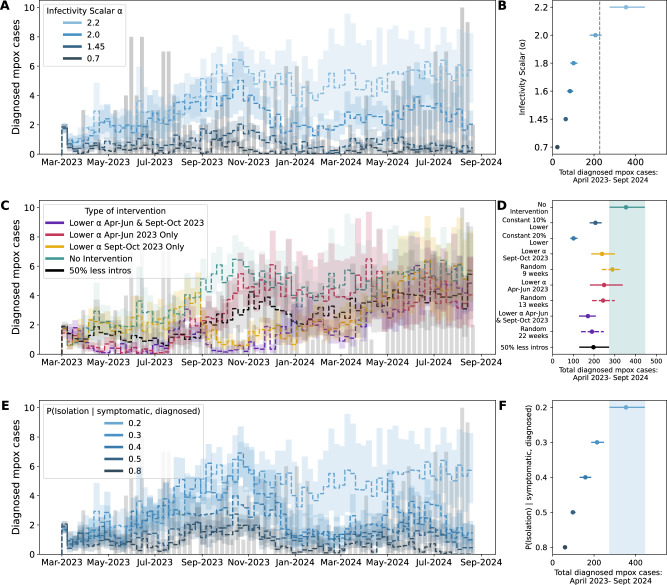
Factors maintaining mpox prevalence and modeling counterfactual public health interventions. After parameterizing our microsimulation model with the number of viral importations estimated via phylodynamics (**A**,** B**), we explored the Infectivity Scalar $\alpha$ parameter that best explains the empirical weekly number of diagnosed mpox cases (gray bars). Line graphs represent the mean weekly number of mpox diagnoses simulated using increasing $\alpha$. Given the non-constant pattern of viral introductions seen in the phylodynamic analysis, we tested different counterfactual scenarios of public health interventions during specific time periods (**C**,** D**) represented by lowering the $\alpha$ to 0.7 while keeping the $\alpha$ at 2.2 during the remaining time. The bold yellow, red, and purple lines represent the simulated weekly number of diagnosed mpox cases under phylodynamic-informed interventions. The black line represents a 50% reduction in introductions. In **D**, we also compared the impact of lowering the $\alpha$ for the same random number of weeks as each specified intervention as well as the impact of constantly lowering the $\alpha$ throughout the entire time period by about 10% and 20% to simulate a constant low-effectivity intervention. The green area represents the upper and lower bounds of the “No Intervention” scenario. We also tested the effect of increasing the probability of isolating upon a symptomatic individual receiving a positive mpox diagnosis on the simulated number of diagnosed mpox cases (**E**,** F**). In **F**, the light blue area represents the bounds of the base model scenario with an $\alpha$ of 2.2. In **A**,** C**,** E**, the gray bars represent the empirical number of mpox diagnoses in LAC. For **B**, the dashed line represents the total empirical number of diagnosed mpox cases from April 2023 through September 2024. For all panels, we calculate the uncertainty of our microsimulation results via bootstrapping with 500 samples to estimate 95% uncertainty intervals for each weekly simulated estimate. Source data for the empirical case numbers are provided as a Source data file; simulated data can be found on https://github.com/blab/mpox-la/tree/main/data/microsimulation_results.

After adding the time-varying weekly number of estimated introductions into our model, we found that our model is able to recapitulate a similar number of diagnosed mpox cases in LAC as in the empirical data (Fig. 5A, B). To do so, however, required increasing $\alpha$ starting in March 2023. We tested different $\alpha$ levels from 0.7 to 2.2, whereby 0.7 represents the decreased sexual activity following the peak of the 2022 mpox outbreak in LAC, and 2.2 represents the baseline $\alpha$ during the beginning of the 2022 outbreak. By comparing the simulated number of mpox diagnoses with the empirical case counts from LAC, we found the optimal $\alpha$ to be 2.0, which represents a significant return in sexual behavior when compared to late 2022 (Fig. 5A, B, dark blue lines). Given that phylogenies only capture successful introductions and that only a number of these introductions are sequenced, the phylodynamic-estimated number of introductions into LAC is expected to be a lower bound. We found that even when doubling the number of estimated importations, both time-varying viral introductions and a subsequent increase in sexual behaviors are still needed to recapitulate the empirical number of mpox cases, adding further support to our conclusion (Fig. S13). We also tested the sensitivity of our findings by increasing and decreasing the average effectiveness of mpox vaccination against infection after 1 year by 25% (from a baseline 1-year average effectiveness drops to 50% of its peak value) and find that the cumulative number of empirical mpox cases still falls within the 95% CI of $\alpha$ of 2.0 (Fig. S14). While we input introductions in our base model as being both diagnosed and symptomatic (See “Methods”), when importations are modeled as asymptomatic and undiagnosed (Figs. S15 and 16), we see that the $\alpha$ needed to recapitulate empirical trends is similar but lower as importations are allowed to contribute more time in the model ($\alpha$ is between 1.8 and 2.0 instead of between 2.0 and 2.2 when introductions are modeled as both diagnosed and symptomatic). Ultimately, all explored model specifications of viral importations support a significant return to near baseline sexual activity.

We also calculated *Rt* from our microsimulation model by tracking the weekly number of secondary cases for each infectious individual (Figs. S11B and S17). For $\alpha$ of 2.0, we found a median *Rt* to be 0.53 (Fig. S17), which is lower than the median *Rt* estimated from other methodologies but in concordance with the *R0* estimation from cluster distributions (Fig. S11B). Additionally, we see a median *Rt* significantly below 1 even at a higher $\alpha$ of 2.2 (median *Rt:* 0.62, Fig. S17), which in Fig. 5A, B resulted in an overestimate of the estimated number of mpox cases. This finding, together with the *R* calculated from the distribution of cluster sizes, suggests that the true median *Rt* during 2023–2024 is significantly below one.

### Counterfactual scenario modeling reveals the potential impact of public health interventions in curbing mpox case counts

We employed our phylo-informed microsimulation model to explore the impact of various potential public health interventions on mpox spread in LAC from 2023 through 2024. Given the time-varying nature of viral introductions seen in the phylodynamic analysis (Fig. 4), we tested the impact of uniformly lowering transmission pressure during the months of highest viral introductions (April through June and September through October in 2023) by lowering the $\alpha$ to 0.7 (Fig. 5C, D). We lower $\alpha$ to simulate significantly reduced sexual behavior prompted via an unspecified public health intervention. During the months without public health intervention, we kept $\alpha$ at the baseline of 2.2. We tested the specificity of our proposed counterfactuals by lowering $\alpha$ to 0.7 for the same number of weeks but selected at random (Figs. 5D and S18).

Our analyses showed that while lowering $\alpha$ during one period of high viral introductions resulted in lower but similar cumulative numbers of total simulated cases when compared to the scenario without intervention, targeting both periods of high-intensity introductions during 2023 resulted in a reduction of almost half of all simulated cases over the entire time period. (Fig. 5C, D, dark purple line). Additionally, we test the impact of interventions that target viral introductions by lowering viral introductions by 50% (Fig. 5C, D black line, S19), showing that it also resulted in about 50% less cumulative cases. In Fig. 5D, we also compared the effect of high-intensity public health interventions only during the high importation months vs having a low-efficacy intervention occurring throughout the entire time period. We simulate this counterfactual by lowering $\alpha$ by approximately 10% and 20% ($\alpha$ of 2 and 1.8 compared to 2.2, respectively). We find that intensely targeting just one time period during 2023 had a similar impact as a constant low efficacy intervention that lowered $\alpha$ by 10% for the entire time period between 2023 and 2024. We also found that having a constant intervention was often more impactful than targeted interventions, suggesting a transmission dynamic dominated by introductions rather than continued local transmission.

Notably, our targeted counterfactual intervention strategies were applied only in 2023. Despite having significant reductions in simulated cases during that year, we see that by the summer of 2024, our model simulates nearly identical weekly case counts for all counterfactuals when compared to our scenario without interventions (Fig. 5C), similarly highlighting how new viral importations, rather than continued local transmission, could be the main contributor of empirical case counts in LAC. The rebound in cases driven by viral importations in 2024 could explain the similar cumulative impact of our targeted interventions only in 2023 when compared to the same number of weeks selected at random (Fig. 5D horizontal dashed lines, Fig. S18).

We also analyzed the impact of increasing the probability of isolation given an infected individual was symptomatic and subsequently diagnosed with mpox (Fig. 5E, F). To do so, we fixed $\alpha$ to the baseline of 2.2 and increased the probability of isolation starting at 0.2, which represents the baseline model. We found that increasing the probability of isolation by increments of 0.1 resulted in large decreases in the total number of diagnosed mpox cases throughout the time period.

## Discussion

After decades as a predominantly regional infection, the mpox virus spread globally in 2022, mostly via sexual networks. While the epidemic in 2022 has been extensively studied^5,6,15,16^, very few studies have investigated the dynamics of mpox clade IIb in 2023 onwards, when cases remain low and sporadic, but resist elimination^19^. Here, we combine phylodynamic and microsimulation modeling approaches to describe the 2023–2024 dynamics of mpox transmission in Los Angeles County, a diverse, metropolitan US County with a high level of rapid genomic mpox surveillance. We show how imported mpox cases and the heavy-tailed pattern of local transmission define the sporadic nature of mpox cases in this large population center, and how the return of typical sexual behaviors alongside a median *Rt* below one might explain the current case trends.

A major strength of our study is the combination of Bayesian phylodynamics inference and microsimulation modeling to help address these knowledge gaps. Both phylodynamic analysis and mathematical modeling have played a crucial role in understanding infectious disease dynamics as well as in informing public health decision-making^30^. Independently, however, both methods have limitations: understanding the interplay of local transmission and viral importations is difficult via case counts alone^11,31^, limiting the power of the microsimulation to capture these dynamics; phylodynamics works to understand shared ancestry as it relates to transmission, making it difficult to simulate counterfactual scenarios and capture dynamics of low-incidence pathogens. Prior work has helped highlight the utility of combining these two approaches^32,33^ but has been limited by the use of deterministic compartmental models and maximum-likelihood phylogenetic methods that are sensitive to differential sampling.

Here, we jointly model both the rate of importation into LAC and local mpox dynamics using Bayesian coalescent phylodynamics, and use those results to inform a stochastic microsimulation model to understand low-incidence pathogen transmission and simulate counterfactual public health interventions. Our work is tailored to the local, heterogeneous demographic and epidemiological landscape of LAC, and models the interplay between local transmission and introductions to understand local mpox dynamics. Ultimately, our study serves as a model for understanding factors that maintain low-level viral disease prevalence in a large, diverse, heterogeneous metropolitan US region.

Our analysis demonstrates that both local transmission and mpox importation contribute to the ongoing sporadic pattern of mpox spread in a large urban center. In alignment with other studies^13,15,34^, we show that most mpox importations lead to a singleton (one case without onward transmission), but a small number of importations transmit to more than ten downstream cases, which are successful in dominating local circulation (Fig. S9). This pattern suggests that identification and intervention, either via vaccination or behavior change, in the small proportion of mpox importations that lead to a large number of local cases could have an outsized impact on the overall dynamics of mpox spread. Local public health efforts to promote vaccination among communities disproportionately impacted by mpox that are community-centered and located in community settings through the use of mobile vaccination teams have encouraged vaccine uptake and provided education regarding sexual behavior harm reduction strategies to prevent mpox transmission^35,36^.

Our results further show that importations of mpox in Los Angeles County varied over time, with a large number of viral importations occurring in mid-fall and spring in 2023 and 2024. These insights may be particularly useful for the formulation and deployment of public health campaigns that promote vaccination and sexual behavior modification strategies. Our counterfactual modeling showed that targeting both of these time periods has the potential to significantly reduce the number of mpox cases (Fig. 5B), allowing for more precise targeting of public health resources. Our results showed, however, that this form of public health action is most effective when multiple time periods are targeted, and that case counts will eventually return to baseline without recurring interventions, suggesting the need for continual implementation of public health action rather than just singular, one-off interventions. When we compared our targeted interventions to a low-efficacy constant public health intervention, we found that high-intensity targeting during just 2–5 months when introductions were highest had a similar effect to continual, low-efficacy interventions, suggesting that identification of high introduction time periods could allow for prioritization of public health effort and showing the high-impact potential of genomics and microsimulation-informed campaigns. Timing social marketing campaigns^37,38^ and vaccine clinics based on patterns of mpox seasonality are promising, as are strategies that focus on raising awareness for travelers and their sexual networks^12^.

Given that the estimates from both the distribution of cluster sizes and the phylodynamics-informed microsimulation model show a mean *Rt* below one (Fig. S11), our results suggest that the true median *Rt* of mpox in LAC during this time period is most likely well below one (which is highly probable as seen in Fig. S11A). This is in concordance with the observation that our microsimulation model needs viral importations to maintain a low case incidence following the 2022 epidemic (Fig. S12D), and with the similar case counts by the summer of 2024 in all our counterfactual scenarios in Fig. 5C, suggesting that weekly case counts are driven by patterns of viral introductions more than by prior levels of local transmission.

The *Rt* results suggest a “stuttering chains” dynamic whereby viral importations result in a heterogeneous distribution of secondary cases but eventually go extinct, which is what we observed in Fig. 4A. Therefore, a consistent inflow of viral introductions is needed to maintain the low case counts. Due to the limitations of passive public health surveillance, stuttering chains can often become “entangled”, resulting in persistent case counts that result in an overestimation of the effective reproductive number as previously shown^24^. This phenomena can be seen in Fig. S7A where the majority of clusters go extinct quickly, but some overlap, giving the impression of constant incidence without exponential growth. Our “entangled stuttering chain” hypothesis is further supported by Fig. S17, where our genomics-informed microsimulation model shows that even at an $\alpha$ of 2.2, which we know overestimates the number of diagnosed mpox cases in LAC (Fig. 5B), the median *Rt* is still significantly below 1 (median *Rt* with $\alpha$ of 2.2*:* 0.62), suggesting the true median *Rt* to be around 0.53 (with $\alpha$ of 2.0).

We expect our estimates of *R* from the distribution of cluster sizes to be artificially elevated as sequencing and phylogenetics are more likely to capture successful and larger clusters than introductions with no secondary cases, artificially increasing the mean cluster size and resulting in an overestimation of *R*^24^. We note that there is high variability in the estimates of median *Rt* regardless of the methodology used (Fig. S8), highlighting the difficulty of estimating these epidemiological parameters at periods with low incidence^39^ and that methods that fail to account for the impact of introductions overestimate the *Rt*^31^ (Fig. S11). Reductions in disease prevalence are often not easily captured in coalescent models, as effective population sizes decrease linearly with decreasing prevalence, but also increase linearly with decreasing incidence^40,41^. This limitation of coalescent models most likely explains why the average phylo Rt even when corrected for the absolute number of introductions, is not further below one, and highlights the importance of combining phylodynamics with microsimulation modeling for understanding pathogens of low incidence. Together, our evidence suggests that the true median *Rt* of mpox in LAC from 2023–2024 is around 0.53 and that time-varying peaks of importations often lead mpox to establish stuttering chains in a densely-connected sexual network that can last until the next peak of introductions.

We used our phylo-informed microsimulation model to uncover factors maintaining the observed low-level mpox prevalence and to test actionable public health interventions. Since our microsimulation model, despite being informed with viral importation estimates, required a recalibration of the Infectivity scalar $\alpha$ parameter, our modeling suggested the low-level, but persistent number of mpox cases in LAC can be explained by a combination of waves of viral introductions, a median *Rt* below one, and a return to near-baseline sexual behavior in 2023–2024. Previous work using online surveys of MSM in North America have shown that more than 78.4% of surveyed individuals who had modified their sexual behavior in response to the 2022 epidemic had reversed their adaptations by May of 2023, showing the plausibility of our results^29^. Of note, both sexual behavior and travel vary by season^6,42,43^, often peaking in summer months, when the 2022 outbreak in LAC began; therefore, the baseline $\alpha$ of 2.2 may represent an upper bound of sexual activity since it was established using only 5 weeks between June-July 2022^18^.

We demonstrate that importations of mpox into LA County follow a roughly seasonal pattern with peaks in August-September and March (Fig. 4), but the importations at these times do not appear to stem from the same geographical locations (Fig. 2b). While we suspect return from summer (LGBTQ+ Pride, an annual celebration of rights, equality, and culture) and holiday travel within the most impacted subpopulations for mpox infection may play a role in these peaks, additional work using data from the International Air Transport Association^6^ and case-specific metadata to determine the scale of travel to Los Angeles at these times could provide more mechanistic detail underlying this pattern. Previous work shows that nearly 20% of MSM travel with a primary goal of engaging in anal sex without a condom^44^, a potentially useful population for mpox outreach. Seasonal patterns in STIs in the US also show peaks in March and August, with the authors suggesting travel relating to summer vacation and university spring breaks as a potential cause, although the data are now over 20 years old^45^. University-related travel may likewise provide an opportunity for mpox vaccination outreach if younger individuals are newer to sexual networks and have not yet received a complete vaccine series and are therefore immunologically naive^46^

While our results offer a mechanistic explanation for present-day transmission dynamics and reveal potential avenues for public health interventions, other factors such as heterogeneity in immunity duration post vaccination or infection^47,48^, or turnover of susceptibles, potentially from younger individuals reaching sexual primacy, might still be impactful. Future work that combines line-level metadata regarding each infection that contains information regarding age, infection history, vaccination status, and zip code of residence, and is matched to viral genomic information, could further elucidate the nuanced mechanisms promoting mpox transmission.

Given the potential return of baseline sexual behavior, the infection control strategies during the ongoing mpox outbreaks might be different than those during the 2022 epidemic^12,23,29^. For example, we tested the impact of increasing the probability of isolation after a symptomatic, infected individual receives a positive diagnosis. We found that increasing the probability from 0.2 to 0.3 resulted in a lower number of diagnosed mpox cases than seen in empirical case counts, highlighting a potential target for public health intervention. Prior modeling work that accounts for the length of viral shedding has shown that isolating three additional days after mpox lesion resolution is sufficient to eliminate more than 95% of post-diagnosis transmission^49^. The authors of that work also note that individual viral shedding kinetics are heterogeneous and that a testing-based isolation strategy could reduce the total time of isolation. Researchers have found, however, that individuals who have previously experienced mpox-like symptoms show a lower willingness to self-isolate after a positive diagnosis, suggesting the need for a more tailored approach for previously-infected individuals^50^. Further work is needed to determine the most effective method of isolation that balances the risk of transmission with the desire for social and sexual contact. For example, prior research has shown that, after adjusting for relevant covariates, engaging in condomless receptive anal sex with an individual with mpox symptoms had the highest association with increased risk of mpox transmission^51^, suggesting that a modification of sexual behavior rather than complete abstention could be a potential harm-reduction strategy. The authors found a potential association between sharing bedding or clothing and the risk of transmission in an unadjusted analysis, but the association was lowered toward the null and nonsignificant when adjusted for relevant covariates, highlighting the need for further work on the risk of non-intimate contact in mpox transmission.

Our results have limitations to note. First, despite our use of all publicly available mpox genomes from LAC, the changing proportion of cases successfully sequenced and uploaded from LAC (Fig. S1) will impact the chance that a case shows up in our data through the period studied. Our phylodynamic analyses are conditioned on the inferred sequence clusters from LAC, which are dependent on the integration of contextual sequences from the US and other regions into a temporally resolved phylogeny. It is possible that differential sampling from other locations could impact our identified clusters and, therefore, our estimates on the rate of introduction. Our simulation analysis, which we downsample different proportions of contextual sequences, however, shows a limited impact on the number of clusters identified as well as the mean cluster size (Fig. S3A). Limited mpox sequence diversity, especially during periods of rapid transmission such as at the beginning of the 2022 epidemic, could affect our ability to break up larger clusters. This might lead to collapsing multiple introductions into LAC into shared clusters, although prior work has shown that APOBEC3 editing associated with human-to-human transmission of mpox results in a mutation rate similar to RNA viruses^6,52,53^. While it would be optimal to explicitly account for locations outside of LAC, ideally through a generalized linear model (GLM) approach that would also help ameliorate the limited sequence diversity, prior work has shown the high computational cost of these approaches^6^. Our approach allows for Bayesian analysis of mpox dynamics within LAC in less than a day, while phylodynamic approaches with a GLM and explicit modeling of different contextual locations have been shown to take upwards of a month. Bayesian coalescent models assume random sampling of infected individuals, meaning that targeted sampling, such as superspreader events or contact tracing, could bias our phylodynamic estimations, although our simulation results show that our models are able to robustly capture complex simulated dynamics that incorporate superspreading (Figs. S9 and 10). Additionally, phylogenies only capture successful introductions into LAC that were ultimately sequenced, meaning that parameterizing our model with the estimated absolute number of introductions inherently underestimates the number of true viral introductions. While informing our model with the estimated absolute number of introductions was necessary due to the underlying microsimulation model structure, our main conclusion – both time-varying viral introductions and an increase to near-baseline sexual activity are needed to explain current mpox dynamics in LAC – is robust, even when we doubled the number of importations estimated (Fig. S13). Future work should focus on parameterizing models with the rate of introductions or the percentage of cases due to introductions.

While we calibrated our microsimulation model using vaccination data from the LAC Department of Public Health^54^, the model does not explicitly account for seasonal variations in mpox vaccination rates, such as the observed increases from May to September 2023 and from July to September 2024 (Fig. 1C). Despite this, the microsimulation model successfully captures the overall vaccination trends by dosage and subgroups, including HIV status, as illustrated in Fig. S20. Given the low likelihood of reinfection following mpox infection^55^, our model assumes only waning vaccine-induced immunity, which may increase the number of susceptible individuals over time. Of note, the Infectivity Scalar ($\alpha$) is a global parameter and does not capture heterogeneity in sexual behaviors or other unmeasured mechanisms that might modify the risk of transmission. While the model incorporates age- and race-stratified mixing patterns, individual-level transmission risk variation within those demographic groups is not accounted for, and nor is variation over time within those groups, although prior work has shown that collective behavior assumptions can effectively approximate population-level mpox dynamics^55^. Microsimulation results also rely on the assumption that $\alpha$ remains constant after mid-2022. In reality, transmission pressure and sexual activity may have fluctuated during the analysis period, creating small variations in weekly case counts. However, there is a lack of reliable data on these short-term behavioral changes, and adopting a time-varying $\alpha$ risk overfits transient noise in the surveillance data. Consequently, we assumed a constant $\alpha$, which means the model does not match empirical case counts exactly week by week, although it successfully captures the overall epidemic trajectory. Additionally, changes in $\alpha$ in our model were derived through calibration and are not based on direct observation; as such, an unobserved, time-varying effect that modified transmission rates during the analysis period could lead to bias in our $\alpha$ calibration. To mitigate this possibility, we account for as many known modifiers of mpox incidence as possible, given the available data (the model includes testing, diagnosis, treatment, disease progression, and recovery rates; see ref. ^18^ for details). Additionally, our counterfactual scenarios simulate only a generalized increase in $\alpha$ during specified periods, which may not fully reflect the true dynamics of disease interventions. Notably, we bounded our modeled interventions in the realm of plausible scenarios that could be enacted by public health agencies. While complete elimination of viral importations (as seen in Fig. S12E) or complete cessation of sexual activity would likely result in elimination of locally-transmitted mpox, such strategies are implausible to implement. Finally, while we addressed stochastic uncertainty using multiple model iterations and bootstrap resampling, we did not perform a full probabilistic sensitivity analysis across all model parameters. However, we partially assessed parameter uncertainty by conducting sensitivity analyses on key inputs, such as vaccine efficacy (VE).

In conclusion, our results suggest that the persistent transmission of mpox in 2023-2024 in a large urban US county can be explained by a combination of time-varying viral importations, a median *Rt* significantly below one, and the return of baseline sexual behaviors that were altered during the 2022 mpox epidemic. Our modeling supports that education and support for mpox patients, such that they can maintain isolation from sexual networks while infectious and symptomatic, may decrease the number of mpox cases in large urban centers. Further, messaging and targeted vaccination around travel, especially in mid-fall and spring, may decrease the number of clusters generated by mpox importations during this time. Our synergistic phylodynamic and microsimulation approach can reveal factors in ongoing mpox dynamics that lead to significant local spread and can be leveraged by local health departments for specific health interventions.

## Methods

### Ethics approval

All data utilized in this study are publicly accessible and consist exclusively of appropriately anonymized viral genomic sequences and aggregate epidemiological information. This work therefore, does not meet the regulatory definition of human-subjects research and so does not require institutional review board (IRB) approval or informed consent.

### Mpox case count data source

Data on the number of diagnosed mpox cases in Los Angeles County were downloaded from the Los Angeles County mpox data dashboard (http://publichealth.lacounty.gov/media/monkeypox/data/index.htm; last accessed on 01-20-2025).

### Estimation of mpox incidence, prevalence, and effective reproduction number via case counts

To jointly estimate mpox case incidence, prevalence, and effective reproduction number, we used the renewal equation framework from Figgins and Bedford^56^. Similar to Paredes et al.^6^, the time-varying effective reproduction number (i.e., the average number of secondary cases infected by a single primary case) was modeled using a 4th-order spline with 5 evenly spaced knots, assuming a discretized gamma-distributed generation time with mean 12.6 days and standard deviation 5.7 days^57^. Case counts were modeled using a Poisson distribution. This model produces posterior estimates of daily incidence (defined as the number of newly infected individuals in absolute counts) and effective reproduction number.

Models were fit to aggregated case counts for each region using full-rank stochastic variational inference. Optimization was performed using the ADAM optimizer with a learning rate 4e-3 and for 50,000 iterations, and 500 samples were drawn from the approximate posterior.

As an additional comparison, we also independently estimate *Rt* using case counts alone via EpiFilter, which has been found to be more robust during periods of low case incidence^39^. To calculate the Rt, we assume a gamma-distributed serial interval of 8.7 days estimated by Ponce et al.^58^.

To estimate the proportion of cases that were sequenced, mpox incidence estimated by the above renewal equation framework was aggregated into monthly estimates for year; the same was done for the number of sequences from LAC. The monthly incidence was then divided by the number of monthly LAC sequences. Due to the limitations of the renewal equation framework (not accounting for overdispersion, strong smoothing) as well as the stochastic nature of genomic sequencing, some months were found to have more sequences than estimated cases. In this situation, we created a ceiling of 100% as a way to bound the variance of estimates.

### Microsimulation model

#### Model overview

In this study, we utilized an individual-based Markovian microsimulation with a weekly cycle to project the dynamics of the 2022 mpox outbreak among MSM in LAC^18^. The model simulates transmission, diagnosis, vaccination, isolation, and treatment, with individuals transitioning through health states (susceptible, exposed, asymptomatic, symptomatic, diagnosed, isolated, treated, and recovered) based on probabilities that vary by age, race/ethnicity, and HIV status. Transmission occurs exclusively through sexual contact during the symptomatic phase. The original model was run from July 2022 to March 2023 to generate a realistic baseline population, which served as the starting population for the present analysis covering March 12, 2023, to October 27, 2024 (85 weeks). The model schematic is shown in Fig. S21, and the key initial conditions and weekly transition probabilities are summarized in Table S1.

For the current study, we refined the model using updated data and improved vaccine modeling. The original model had been calibrated and validated against diagnosed cases and vaccination uptake from July 2022 to March 2023, stratified by age, race/ethnicity, and HIV status. Building on this foundation, we incorporated updated surveillance data disaggregated by dose number and HIV status for March 2023–October 2024 (Fig. S20). We also replaced the previous assumption of constant VE with a time-varying, piecewise linear VE function. VE is modeled separately for people with HIV (PWH) and HIV-negative individuals and for one-dose versus two-dose regimens. Following vaccination, VE increases from zero to its peak over 2 weeks, remains at peak for 2 weeks, and then wanes linearly over 52 weeks. In the base case, VE declines to 50% of its peak 1 year after vaccination (e.g., two-dose VE in HIV-negative individuals declines from 87.8% to 43.9%)^47,48,59^). Sensitivity analyses evaluate alternative waning assumptions of 25% and 75% of peak efficacy (see Fig. S22 for the vaccine waning efficacy visualization).

#### Modeling infection risk

The probability of mpox infection for an individual, denoted as $P({infection})$, was determined by the interplay of multiple factors capturing demographic variation and behavioral dynamics in the population. In our weekly-cycle model, $P({infection})$ represents the probability that a susceptible individual becomes infected within a single week. Specifically, it was defined as:

1 $$P({infection})=(\min )\,[(1-{\prod }_{{d}_{p}\in D}(1-{\alpha \gamma }_{a}{\beta }_{d}\frac{{I}_{{dp}}}{N{d}_{p}}){P}_{d}{M}_{{d}_{p}})\cdot {{RR}}_{{HIV}},1]$$

Where:

$D:$ Set of demographic groups considered in the mixing matrix

${d}_{p}:$ Partner demographic group

$$\alpha :$$ Infectivity scalar

${\gamma }_{a}:$ Age-specific calibration parameter for the susceptible individual, where $a\in \{15-24,\,25-34,\,35-44,\,45-100\}$

${\beta }_{d}:$ Race/ethnicity-specific calibration parameter for the susceptible individual, where ${d}\in \{{Black},{Hispanic},{White}\}$

${I}_{{d}_{p}}:$ Number of infectious (not isolated) individuals in partner demographic group ${d}_{p}$

${N}_{{d}_{p}}:\,$Total number of individuals in partner demographic group ${d}_{p}$

${P}_{d}:$ Average number of partners for an individual in the demographic group $d$

${M}_{{d}_{p}}:$ Probability that a susceptible individual mixes with partners in demographic group ${d}_{p}$

$R{R}_{{HIV}}:$ Relative risk multiplier applied to individuals with HIV to reflect increased susceptibility

This formulation incorporates group-specific transmission risk, including differences by age, race/ethnicity, and HIV status, together with sexual partner mixing patterns modeled using an age- and race/ethnicity-specific mixing matrix (see Appendix Fig. 3 in ref. ^18^), and variation in weekly partner counts across demographic groups. These components allow infection risk to reflect the underlying structure of sexual networks and demographic variation in exposure patterns.

#### Calibrating and estimating the infectivity scalar ($\alpha$)

The microsimulation uses a calibration parameter we refer to as the “Infectivity scalar” ($\alpha$) to adjust the probability of infection and align transmission dynamics with observed trends. In the initial model setup, $\alpha$ was calibrated through a grid search across 2.1–2.5, in combination with the asymptomatic infections in the initial cohort, and was set to 2.2. This value aligns with empirical trends from the early phase of the mpox outbreak in LAC, reflecting the high transmission levels observed prior to August 2022. Following the pronounced peak and subsequent sharp decline in reported cases during late summer and early fall of 2022, $\alpha$ was recalibrated using a grid search across 0.6–0.9 and set to 0.7, which likely reflects the impact of enhanced public health guidelines and changes in public behaviors during that period. Details of the grid-search procedures used to determine these $\alpha$ values are provided in Liang et al.^18^. This one-time adjustment allowed the model to capture both the elevated transmission in mid-2022 and the sharp reduction afterward.

The calibrated model accurately reflected the observed transmission trends. By December 2022, the seven-day average number of new cases had dropped below two, and by March 2023, the model predicted the potential cessation of local mpox transmission in LAC in the absence of external introductions. Further details on calibration, validation, and full model inputs can be found in Liang et al.^18^.

In the present study, $\alpha$ is held constant within each baseline simulation scenario and is varied only across scenarios within the empirically supported range of 0.7– 2.2. We did not implement continuous or weekly time-varying calibration because empirical fluctuations after March 2023 are small and noisy. However, in a subset of intervention scenarios, $\alpha$ is intentionally reduced during predefined time windows to represent short-term reductions in sexual activity associated with public health interventions. These changes are imposed mechanistically to reflect hypothesized behavioral responses rather than calibrated to short-term fluctuations in reported case counts. This approach preserves the interpretability of intervention effects while avoiding overfitting to transient noise in the surveillance data.

#### Simulating introductions and integrating phylodynamic estimates

While the model suggests the potential cessation of local mpox transmission in LAC by March 2023, the empirical sporadic cases and minor surges observed afterward highlight the need to account for external viral introductions. To explore this, we first tested hypothetical scenarios in which 5, 10, or 15 symptomatic cases were introduced into the model each week. These cases were randomly drawn in proportion to the simulation demographic and were allowed to infect susceptibles in the model.

Simulation results showed that higher levels of constant weekly importations led to more local transmissions. However, these simplified scenarios did not reproduce the observed temporal patterns in mpox case counts, nor did they explain the persistence of low-level transmission (Fig. 2D). To better align the model with real-world dynamics, we integrated time-varying viral importation estimates from our phylodynamic analysis. By incorporating both the number and timing of imported cases more precisely, we improved the model’s ability to simulate short-term surges and temporal patterns in mpox case counts. Linking these external seeding events with behavioral changes modeled via the Infectivity Scalar ($\alpha$), the combined framework helped explain how ongoing transmission can arise even after local transmission declined. As in the hypothetical scenarios, these phylodynamics-informed imported cases were treated as already diagnosed upon introduction, ensuring they were not counted as newly diagnosed within LAC.

#### Running the model and analyzing output

Due to the stochastic nature of our model, we ran twenty independent iterations to capture inherent variability in the outcomes. To estimate uncertainty intervals for key metrics, such as incident case counts, we employed a bootstrap approach with 500 samples. Each sample consisted of a resample (with replacement) from the ten iterations. We calculated weekly averages for each sample, forming the data into a 500 $\times$ 85 matrix, where each row represents a bootstrap sample, and each column corresponds to a week. From this matrix, we computed the mean, lower bound (2.5th percentile), and upper bound (97.5th percentile) of these averages. This methodology captures the model’s stochastic variability and associated uncertainty in predicted outcomes. We assessed the impact of parameter uncertainty through sensitivity tests on two alternative assumptions for VE, in which efficacy drops to 25% and 75%, compared to the base case 50% (see Fig. S14).

*Rt* was estimated by multiplying the average number of secondary infections per infected individual by the duration of infectiousness.

All simulations were programmed in MATLAB and executed on the high-performance computing facilities at the Center for Advanced Research Computing (CARC)^60^. Using a parallel computing setup, we ran 20 iterations simultaneously, with each batch for a given scenario completing in approximately 13 min on a computing node with at least 20 available CPU cores. This approach allowed us to efficiently scale the number of iterations while maintaining a practical runtime for model calibration and scenario analysis.

### Phylodynamic analysis

#### Genomic data and maximum likelihood tree generation

All available MPXV sequences were downloaded from GenBank on 01-20-2025. Sequences with an ambiguous date of collection in the month column, with a sample collection earlier than January 2022, and flagged as being low quality by Nextclade (https://docs.nextstrain.org/projects/nextclade/en/stable/user/algorithm/06-quality-control.html)^61^ were excluded. Given that mpox transmission in the United States is driven by clade IIb viruses, sequences from other clades were also excluded, resulting in 7859 genome sequences included in our analysis.

A temporally-resolved phylogeny was created using a modified version of the Nextstrain^20^ mpox workflow (https://github.com/nextstrain/mpox), which aligns sequences against the MK783032 (collection date: Nov. 2017) reference using nextalign^61^, infers a maximum-likelihood phylogeny using IQ-TREE^62^ with a GTR nucleotide substitution model, and estimates molecular clock branch lengths using TreeTime^63^. The resulting phylogeny specific to this dataset can be found at https://nextstrain.org/groups/blab/mpox/allcladeIIseqs

#### Geographic scales

Due to the low number of sequences from various countries, we analyzed mpox spread with a focus on large metropolitan US cities and areas that have the highest level of mpox sequencing effort. Our focus areas were: Los Angeles County, California; Washington State; Cook County, Illinois; New York City, New York; California without Los Angeles County; North America excluding the areas previously mentioned; and Global regions outside of North America.

Given that the Los Angeles County Department of Public Health (LA DPH) sequences the mpox cases for LAC, we assume that any genome labeled as being sequenced by LA DPH was sampled in LAC, while those sampled by the California Department of Health (CDPH) were sampled in locations within California but outside of LAC. From these 719 genomes, the dataset was filtered down to 497 by LA DPH to remove duplicated sequences from the same individual and samples that were collected outside of LA DPH. Despite this, there is always a small chance that CDPH might have received and sequenced an LAC case, but we expect this to be small and should result in a conservative bias, as misclassification of an LAC sequence as non-LAC would result in smaller clusters and less intense transmission dynamics.

Phylogeographic reconstruction of mpox spread was conducted using the same Nextstrain workflow via ancestral trait reconstruction^64^ of the aforementioned focus areas. This was done using the “augur traits” function^65^.

#### Clustering

To identify local outbreak groups in Los Angeles County, we clustered all LAC sequences based on inferred internal node location. Following Müller et al.^66^ and Paredes et al.^67^, we used a parsimony-based approach to reconstruct the locations of internal nodes. Briefly, using the Fitch parsimony algorithm, we inferred internal node locations by considering only two sequence locations: LAC and then anywhere else. We then identified local outbreak clusters by selecting groups of sequences in which all their ancestral nodes were inferred to be from LAC, up until there was a change in location.

We then plotted the mean cluster size and the number of local clusters per month by using the month of collection for the first collected sequence of each identified outbreak cluster over time.

#### Estimating population dynamics jointly from multiple local outbreak clusters

To analyze the local transmission dynamics of mpox in LAC from 2022 to 2024, we used a multi-tree coalescent model to jointly model mpox dynamics from the inferred outbreak clusters, originally described in Müller et al.^66^. Briefly, we assumed that each identified cluster was the result of a single introduction into LAC and that the sequences that make up each cluster were the result of local transmission. Doing so allowed us to model mpox transmission as a structured coalescent process where the migration history is conditional on the clustering done a priori. The model allows mpox lineages to coalesce within LAC but can also originate from outside the sampled area. The migration history of the coalescent process is conditioned on the identified transmission clusters, whereby we assume that the introduction event into LAC occurred prior to the most recent common ancestor of the sequences in each cluster. This time of introduction is explored via an MCMC run. We used a skyline approach to estimate both the effective population size (*Ne)* and rates of introduction throughout time using predefined change points (every 7 days), assuming exponential growth or decline between each change point. We ran two independent chains, and employed a strict molecular clock with a uniform distribution from 0 to 1 and a fixed value of 6 × 10^−5^^6,20^ and an HKY + Γ nucleotide substitution model with an estimated $\kappa$. We also repeat the analysis to test the sensitivity of our results with the following specifications: with a GTR + Γ substitution model with the same fixed clock rate and estimated frequencies and transitions; and with an eight-category discrete Γ prior instead of four^68^.

Similar to Müller et al.^66^, we apply an exponential coalescent model with time-varying growth rates by accounting for correlations between adjacent *Nes* via the skyride approach, which assumes the log of adjacent *Ne* is normally distributed with a mean of 0 and an estimated variance. We also assumed the differences in growth rates were normally distributed with a mean of 0 and an estimated variance. This formulation was validated in Supplementary Fig. 3 of Müller et al.^66^.

#### Deriving and implementing case-based Ne prior

Additionally, we also conduct a separate analysis by allowing the Ne to be informed by the total number of diagnosed mpox cases in each month. In a standard formulation of the coalescent model of infectious diseases parameterized by Susceptible-Infected-Recovered (SIR) dynamics^69^,

2 $${Ne} \, \tau=\frac{I(t)}{2\beta S(t)}$$

where $\tau$ refers to the generation time, $I(t)$ and $S(t)$ to the time varying prevalence and number of susceptibles in the population, respectively, and $\beta$ to the transmission rate. We represent the scaler $\frac{1}{2\beta S(t)}$ via $\theta (t)$ so that,

3 $${Ne} \, \tau=I(t)\theta (t)$$

If we assume that the number of diagnosed mpox cases can approximate the prevalence *I(t)*, then we can rewrite the above equation as

4 $$\log ({Ne})=\log (\varTheta )+\log ({cases})$$

To account for time-varying observation noise and variability in the above assumptions, we can add an error term so that,

5 $$\log ({Ne})=\log (\varTheta )+\log ({cases})+\epsilon$$

By rearranging the terms, we get

6 $$\epsilon=\log ({Ne})-\log (\varTheta )-\log ({cases})$$

We then account for correlations between adjacent errors by assuming that the difference in errors is normally distributed with a mean of 0 and an estimated variance.

We implemented and ran these models as an extension to BEAST2 software version 2.6.2^70^ and can be found on https://github.com/miparedes/mab.We performed effective population size and migration rate inference using an adaptive multivariate Gaussian operator^71^ and ran the analyses using an adaptive Metropolis-coupled MCMC^72^ using two chains with a length of $2.5\times 1{0}^{8}$. We repeat our analysis without the rolling mean smoothing, as well as without any cases, to test the sensitivity of our results.

### Posterior processing of phylodynamic analyses

Parameter traces were visually evaluated for convergence using Tracer^73^, tree distributions were visually inspected using IcyTree^74^, and 20% burn-in was applied for all phylodynamic analyses. All tree plotting was performed with baltic (https://github.com/evogytis/baltic), and data plotting was done using Altair^75^, matplotlib^76^, and seaborn^77^.

Following Bedford et al.^78^, persistence time was measured by calculating the average number of days for a lineage to leave LAC, walking backwards up the phylogeny from the tip up until the node location was outside of LAC. We also cycled through the posterior set of trees to find the median time of importation into LAC for each identified local outbreak cluster

#### Estimating the number of importations into LAC for the microsimulation model

The absolute number of viral importation events into LAC was estimated by calculating the number of transitions walking from tips to root in the posterior set of trees and calculating the median as well as the 50% and 95% highest posterior density estimates (HPD).

#### Estimating the percentage of new cases due to introductions

We estimated the percentage of new cases due to introductions for each global region by adapting the methods previously described^6^. Briefly, the percentage of cases due to introductions $\pi$ at time *t* can be calculated by dividing the number of introductions at time *t* by the total number of new cases at time *t*. We first represented the total number of new cases in a region as the sum of the number of introductions and the number of new local infections due to local transmission, resulting in the following equation:

7 $$\pi (t)=\frac{{{\rm{\#}}}\,{{\mathrm{of}}}\,{{\mathrm{introductions}}}(t)}{{{\rm{\#}}}\,{{\mathrm{of}}}\,{{\mathrm{new}}}\,{{\mathrm{local}}}\,{{\mathrm{cases}}}(t)\,+\,{{\rm{\#}}}\,{{\mathrm{of}}}\,{{\mathrm{introductions}}}(t)\,}$$

We estimated the number of new local cases at time *t* by assuming the local epidemic in each global region follows a simple transmission model, in which we derived the number of new cases at time *t* as the product of the transmission rate $\beta$ (new infections per day per infected individual) multiplied by the number of people already infected in that region *I*. For the number of introductions, we similarly assumed that the number of introductions equals the product of the rate of introduction (introductions per day per infectious individual, which we refer to as migration rate *m*) and the number of people already infected in that region *I*. We use the number of infected individuals in the destination region rather than the origin region for calculating the number of introductions since the approximate structured coalescent approach models epidemic processes as backwards-in-time, resulting in the equation containing only information about the number of infected individuals in the destination region (more information on backwards migration rates below). We then rewrote the above equation as

8 $$\pi (t)=\frac{m(t)\,I(t)}{\beta (t)\,I(t)\,+\,m(t)\,I(t)\,}$$

where *I*(*t*) denotes the number of infected people in that region at time *t*. Given the presence of *I*(*t*) in every element, we factored out *I*(*t*) to arrive at

9 $$\pi (t)=\frac{m(t)}{\beta (t)\,+\,m(t)\,}$$

For each region, we considered introductions at time *t* to be the sum of the introductions coming into LA County from outside the region. We define the percentage of new cases due to introductions $\pi$ at time *t* for LAC as

10 $${\pi }_{{LA}}(t)=\frac{{{m}^{b}}_{{LA}\,\to i}(t)}{{\beta }_{{LA}}(t)\,+\,{{m}^{b}}_{{LA}\,\to i}(t)\,}$$

where ${{m}^{b}}_{{LA}\to i}$ denotes the backwards migration rate per lineage per day into LAC from outside and is estimated directly via our multi-tree coalescent model.

In a SEIR transmission modeling framework (employed due to the incubation period of MPXV), the transmission rate $\beta$ is a function of the infectious period $\gamma$, the incubation period $\sigma$, and the exponential growth rate *r* (as adapted from Example 4 in Ma 2020^79^):

11 $$\beta=\frac{{(2r+\gamma \,+\sigma )}^{2}-\,{(\sigma -\gamma )}^{2}}{4\sigma }$$

To compute the growth rate in region *y* for use in calculating the percentage of cases due to introductions only, we assumed that differences in effective population size between adjacent time intervals can approximate the growth rate *r* and thus $\frac{d \left(\right.\log (N{e}_{y})}{{dt}}\,\approx {r}$. In addition, we assumed that $\frac{{dNe}}{{dt}}$ is independent of the rate of introduction. We calculated the growth rate of the effective population size $\frac{{dNe}}{{dt}}$ as

12 $$\frac{d(\log ({Ne}))}{{dt}}=\frac{\log ({Ne}(t+\varDelta t))-\log \left(\right.{Ne}(t)}{\varDelta t}$$

where $\left(\right.{Ne}\left(t\right)$ denotes the effective population size of a region at time *t*. We ran our analysis using weekly time intervals but averaged over 3-week intervals ($\Delta t$ = 3) for the growth rate in order to reduce noise and account for the long generation time for mpox.

We calculated the transmission rate $\beta$ at time *t* in LAC as

13 $${\beta }_{LA}(t)=\frac{ ( 2{(\frac{d\left(\right.\log (N{e}_{LA})}{dt})+\gamma \,+\sigma })^{2}-\,(\sigma -\gamma )^{2}}{4 \sigma }$$

#### Incubation and infectious period estimates for use in phylodynamic analyses only

For the incubation period, we used a mean of 8 days based on prior literature^58,80^. The infectiousness period for mpox has yet to be definitively characterized^81^, as such, we used the estimates of the infectious period (10.9 days) from Jeong et al.^49^ for our main analysis, as they were defined via analysis of viral load and viral shedding in more than 90 mpox cases. To account for variability in this estimate, we also repeated our percentage of cases due to introductions and *Rt* analyses using a mean infectious period of 4.5 days and 21 days (Fig. S5). The mean infectious period of 4.5 days was estimated from the comparison of the generation time of 12.5 days^57^ and the aforementioned incubation period through the formulation of the generation time in Wallinga and Lipsitch^82^. This lower estimate of the infectious period is in concordance with the infectious period estimations from Zhang et al.^83^. The mean estimate of 21 days refers to the average time of resolution of symptoms^84^ and has been previously used as a mainly clinical proxy for infectiousness^85^.

#### Estimating the effective reproductive number Rt from pathogen genomes

We calculated the effective reproductive number *Rt*^82^, the time-varying average of secondary infections from a primary infected individual, in LAC, assuming an exponentially distributed infectious and incubation period of mean, respectively $1/\gamma,$ and 1/$\sigma \,$, yielding

14 $${Rt}=(1+\frac{r}{\gamma })(1+\frac{r}{\sigma })$$

Additionally, we sought to separate out the contributions of introductions versus local transmission to *Rt*_*t*_. To do so, we modified the *Rt* equation to include the percent of new cases from introductions as an estimate of local community spread so that

15 $${Rt}=(1+\frac{r}{\gamma })(1+\frac{r}{\sigma })(1-\pi )$$

where $\pi$ refers to the percentage of new cases due to introductions as described above.

Of note, our *Rt* calculations assume that the change in *Ne* over time is proportional to the change in the number of infected individuals over time.

To further validate our estimates of *Rt*, we fit the estimated cluster distributions taken from the sizes of the identified sequenced outbreak clusters to the formulation in Tran-Kiem & Bedford^22^ which allows for the estimation of *R* and the dispersal parameter *k* and accounts for the probability of a case being detected and sequenced (similar to^24,86^). Given that we use all available sequences and not just identical sequences, we set the probability that a transmission event occurs before a substitution event *p* as 1. We also assume a range of case detection rates from 5% to 100% of all cases detected and then sequenced. We report the results assuming a 5% case detection rate as the most conservative estimate. Similarly, we also explored the probability of observing at least a cluster of size 16 (the largest size found in 2023–2024) among 64 total clusters as a function of the effective reproduction number *R* across a time period, transmission heterogeneity as estimated via the dispersion parameter *k*, and the fraction of infections sequenced. This estimation has been previously derived in other work^21,22^. We explore this probability among R values ranging from 0.1 to 1.6 and *k* values from 0 to 10, assuming a probability of case detection of 5.5% which was estimated to be the average case sequencing rate throughout the 2022 mpox epidemic^6^. While we expect the fraction of infections sequenced to be higher in LAC for 2023–2024 (Fig. S1), we use 5.5% as a conservative estimate, as increasing the fraction sequenced is likely to make even lower R values more likely.

#### Estimating the relative importance of introductions compared to ongoing lineages

To estimate the relative success of introductions over 2-month time windows from March 1, 2023, through December 12, 2024 (the date of the last sequence in our sample), we adapted the methods in Lemey 2021^87^. Briefly, we analyzed the posterior set of trees from our unstructured coalescent analysis to estimate the posterior mean and 95% HPDs of three proportions: the proportion of unique introductions in the time period over the total number of unique persisting lineages and unique introductions; the proportion of unique introductions whose downstream transmission chains persisted at least until the end of that time period over the total number of persistent introductions and ongoing lineages; and the proportion of descendant lineages from these unique introduction events over the total number of descendants circulating after the end of the two month time period. We define a unique introduction as the inferred migration date of an identified outbreak cluster that falls between the start and end date of each two-month time slice. We define an ongoing lineage as a lineage whose inferred date of introduction is before the start date of each time period and exists through the end date. Similarly, we define descendants as the number of leaves (sequences) stemming either from the unique introductions or the ongoing lineages that exist at or after the end date of each time period.

#### Phylodynamic simulations

To test the applicability of our multitree coalescent model, both with the standard implementation as well as our cases-informed *Ne*, we simulated phylogenetic trees under an SEIR model with superspreading^66^. We also assumed a constant force of introduction per unit time into the region. We assumed the number of newly infected individuals to be negatively binomially distributed such that the mean number of introductions at any point in time *t* was equal to *Rt* and the dispersion parameter *k* = 0.3 as previously estimated^6,23^. To approximate real-life sampling dynamics, we parameterized the sampling rate based on the estimated time to present to healthcare in the UK in 2022^88^. We next simulated a structured phylogenetic tree from this approach and then simulated genetic sequences on top of this phylogenetic tree using Seq-Gen^89^, assuming an HKY substitution model, a genome size of 197,000 bps, and a clock rate of $6\times 1{0}^{-5}$, similar to our main analysis above. To understand the impact of undersampling, we also randomly subsampled 50% of the simulated sequences and ran all the simulations via our multi-tree coalescent models. We then compared the estimated *Ne, Rt*, and percentage of cases due to introductions with the same values calculated from the SEIR dynamics.

After simulating a local mpox outbreak with a constant force of introduction and superspreading with two different sequencing schemes, we found that our case-based prior approach is better at capturing temporal trends than analyses using sequences alone (Figs. S9 and 10). While the scenario with a Skygrowth prior on the growth rate analyzing sequences alone had the highest *R*^2^ value when comparing estimated *Rt* and the percentage due to introductions with simulated values, the 95% HPD intervals often failed to include the true value, while the Skyline prior of the *Ne* informed by case counts had a similarly high *R*^2^ while more often containing the true parameter value within the 95% HPD intervals. The case-informed Skyline prior was also found to be more robust to having 50% fewer genomes available when compared to the same model with a Skyline prior but without any case information. Ultimately, all three specifications of our model are able to capture the simulated dynamics, showing the utility of genomic information to inform investigations into local mpox dynamics as well as the added benefit of incorporating epidemiological information into our phylodynamic analyses.

### Reporting summary

Further information on research design is available in the Nature Portfolio Reporting Summary linked to this article.

## Supplementary information

Supplementary Information

Peer Review file

Reporting Summary

## Source data

Source Data

## Data Availability

All sequences are available on GenBank with accession numbers found in the supplementary information as well as in https://github.com/blab/mpox-la/blob/main/final_acknowledgements_table_genbank.tsv. All other de-indentified data to replicate the results can be found on https://github.com/blab/mpox-la/tree/main. Source data are provided with this paper.

## Code availability

Nextstrain builds, BEAST2 XMLs, scripts, sequence information, and de-identified data for the phylogenetic and phylodynamic analyses can be found at https://github.com/blab/mpox-la/tree/main. The code for the microsimulation model developed to study mpox incidence and dynamics is available at https://github.com/citina/microsimulation-mpox, which includes all scripts, parameter files, and usage instructions necessary to replicate the study findings.

## References

[1] Titanji, B. K., Hazra, A. & Zucker, J. Mpox clinical presentation, diagnostic approaches, and treatment strategies: a review. *JAMA* **332**, 1652–62 (2024). 39401235 10.1001/jama.2024.21091

[2] Thornhill, J. P. et al. Monkeypox virus infection in humans across 16 countries — april–june 2022. *N. Engl. J. Med.* **387**, 679–91 (2022). 35866746 10.1056/NEJMoa2207323

[3] Parker, E. et al. Genomics reveals zoonotic and sustained human mpox spread in West Africa. *Nature* **643**, 1343–1351 (2025). 10.1038/s41586-025-09128-2 PMC12310364 40388983

[4] Gigante, C. M. et al. Multiple lineages of monkeypox virus detected in the United States, 2021–2022. *Science* **378**, 560–5 (2022). 36264825 10.1126/science.add4153 PMC10258808

[5] Isidro, J. et al. Phylogenomic characterization and signs of microevolution in the 2022 multi-country outbreak of monkeypox virus. *Nat. Med.* **28**, 1569–72 (2022). 35750157 10.1038/s41591-022-01907-y PMC9388373

[6] Paredes, M. I. et al. Underdetected dispersal and extensive local transmission drove the 2022 mpox epidemic. *Cell* **187**, 1374–1386.e13 (2024). 38428425 10.1016/j.cell.2024.02.003 PMC10962340

[7] Kinganda-Lusamaki, E. et al. Clade I mpox virus genomic diversity in the Democratic Republic of the Congo, 2018–2024: Predominance of zoonotic transmission. *Cell* **188**, 4–14.e6 (2025). 39454573 10.1016/j.cell.2024.10.017

[8] Vakaniaki, E. H. et al. Sustained human outbreak of a new MPXV clade I lineage in eastern Democratic Republic of the Congo. *Nat. Med.* **30**, 2791–5 (2024). 38871006 10.1038/s41591-024-03130-3 PMC11485229

[9] Tuttle, A. Notes from the field: clade II Mpox surveillance update — United States, October 2023–April 2024. *Morb. Mortal. Wkly. Rep*. 2024. Available from: https://www.cdc.gov/mmwr/volumes/73/wr/mm7320a4.htm 10.15585/mmwr.mm7320a4 PMC11115435 38781102

[10] CDC. Mpox. 2025 [cited 2025 Jan 29]. Mpox in the United States and Around the World: Current Situation. Available from: https://www.cdc.gov/mpox/situation-summary/index.html

[11] Cori, A., Ferguson, N. M., Fraser, C. & Cauchemez, S. A new framework and software to estimate time-varying reproduction numbers during epidemics. *Am. J. Epidemiol.* **178**, 1505–12 (2013). Nov 1. 24043437 10.1093/aje/kwt133 PMC3816335

[12] Xiridou, M. et al. Combining mpox vaccination and behavioural changes to control possible future mpox resurgence among men who have sex with men: a mathematical modelling study. *bmjph* **3**, (2025). 10.1136/bmjph-2025-002682 PMC12315014 40756169

[13] Endo, A. et al. Heavy-tailed sexual contact networks and monkeypox epidemiology in the global outbreak, 2022. *Science* **378**, 90–4 (2022). 36137054 10.1126/science.add4507

[14] Volz, E. M., Koelle, K. & Bedford, T. Viral phylodynamics. *PLOS Comput. Biol.* **9**, e1002947 (2013). 23555203 10.1371/journal.pcbi.1002947 PMC3605911

[15] Pekar, J. E. et al. Transmission dynamics of the 2022 mpox epidemic in New York City. *Nat. Med.* **31**, 1464–1473 (2025). 10.1038/s41591-025-03526-9 PMC12092259 40133528

[16] Borges, V. et al. Viral genetic clustering and transmission dynamics of the 2022 mpox outbreak in Portugal. *Nat. Med.* **29**, 2509–17 (2023). 37696933 10.1038/s41591-023-02542-x PMC10579057

[17] Rutter, C. M., Zaslavsky, A. & Feuer, E. Dynamic microsimulation models for health outcomes: a review. *Med. Decis. Mak.* **31**, 10–8 (2011). 10.1177/0272989X10369005 PMC3404886 20484091

[18] Liang, C. et al. A microsimulation model of Mpox in Los Angeles county: implications for future disease prevention and control strategies among men who have sex with men. *Open Forum Infect. Dis.* **11**, S137–45 (2024). 39415828 10.1093/ofid/ofae401 PMC11477083

[19] Leonard, C. M. et al. Mpox Outbreak - Los Angeles County, California, May 4-August 17, 2023. *Morb. Mortal. Wkly. Rep.* **73**, 44–8 (2024). 10.15585/mmwr.mm7302a4 PMC10803096 38236779

[20] Hadfield, J. et al. Nextstrain: real-time tracking of pathogen evolution. *Bioinformatics* **34**, 4121–3 (2018). Dec 1. 29790939 10.1093/bioinformatics/bty407 PMC6247931

[21] Kucharski, A. J. & Althaus, C. L. The role of superspreading in Middle East respiratory syndrome coronavirus (MERS-CoV) transmission. *Eurosurveillance* **20**, 21167 (2015). 26132768 10.2807/1560-7917.es2015.20.25.21167

[22] Tran-Kiem, C. & Bedford, T. Estimating the reproduction number and transmission heterogeneity from the size distribution of clusters of identical pathogen sequences. *Proc. Natl. Acad. Sci. USA* **121**, e2305299121 (2024). 38568971 10.1073/pnas.2305299121 PMC11009662

[23] Maniscalco, D. et al. Role of behaviour change in controlling the 2022 Paris mpox outbreak. *Nat. Health* **1**, 226–237 (2026).

[24] Blumberg, S. & Lloyd-Smith, J. O. Inference of R0 and transmission heterogeneity from the size distribution of stuttering chains. *PLOS Comput. Biol.* **9**, e1002993 (2013). 23658504 10.1371/journal.pcbi.1002993 PMC3642075

[25] Blackburn, D. Epidemiologic and clinical features of Mpox in transgender and gender-diverse adults — United States, May–November 2022. *Morb. Mortal Wkly. Rep*. Available from: https://www.cdc.gov/mmwr/volumes/71/wr/mm715152a1.htm 10.15585/mmwr.mm715152a1 PMC9812441 36580418

[26] Thornhill, J. P. et al. Human monkeypox virus infection in women and non-binary individuals during the 2022 outbreaks: a global case series. * Lancet* **400**, 1953–65 (2022). 36403584 10.1016/S0140-6736(22)02187-0 PMC9671743

[27] McQuiston, J. H. The CDC domestic Mpox response — United States, 2022–2023. *Morb. Mortal Wkly. Rep.* Available from: https://www.cdc.gov/mmwr/volumes/72/wr/mm7220a2.htm 10.15585/mmwr.mm7220a2 PMC10205168 37200231

[28] Delaney, K. P. Strategies adopted by gay, bisexual, and other men who have sex with men to prevent monkeypox virus transmission — United States, August 2022. *Morb. Mortal Wkly. Rep.*Available from: https://www.cdc.gov/mmwr/volumes/71/wr/mm7135e1.htm 10.15585/mmwr.mm7135e1 PMC9472779 36048582

[29] Prochazka, M. et al. Temporary adaptations to sexual behaviour during the mpox outbreak in 23 countries in Europe and the Americas: findings from a retrospective cross-sectional online survey. *Lancet Infect. Dis.* **24**, 1309–18 (2024). 39305907 10.1016/S1473-3099(24)00531-0 PMC11810863

[30] Heesterbeek, H. et al. Modeling infectious disease dynamics in the complex landscape of global health. *Science* **347**, aaa4339 (2015). 25766240 10.1126/science.aaa4339 PMC4445966

[31] Creswell, R. et al. Heterogeneity in the onwards transmission risk between local and imported cases affects practical estimates of the time-dependent reproduction number. *Philos. Trans. R. Soc. Math. Phys. Eng. Sci.* **380**, 20210308 (2022). 10.1098/rsta.2021.0308 PMC9376709 35965464

[32] Reichmuth, M. L., Hodcroft, E. B. & Althaus, C. L. Importation of alpha and delta variants during the SARS-CoV-2 epidemic in Switzerland: phylogenetic analysis and intervention scenarios. *PLOS Pathog.* **19**, e1011553 (2023). 37561788 10.1371/journal.ppat.1011553 PMC10443857

[33] Parino, F. et al. Integrating dynamical modeling and phylogeographic inference to characterize global influenza circulation. *PNAS Nexus* **4**, pgae561 (2025). 39737444 10.1093/pnasnexus/pgae561 PMC11683419

[34] Murayama, H. et al. Accumulation of immunity in heavy-tailed sexual contact networks shapes Mpox outbreak sizes. *J. Infect. Dis.* **229**, 59–63 (2024). Jan 15. 37402631 10.1093/infdis/jiad254 PMC10786257

[35] Holloway, I. W. Lessons for community-based scale-up of monkeypox vaccination from previous disease outbreaks among gay, bisexual, and other men who have sex with men in the United States. *Am. J. Public Health* **112**, 1572–5 (2022). 35981275 10.2105/AJPH.2022.307075 PMC9558182

[36] Osmundson, J. et al. Mobile Mpox vaccination in New York city provided flexible community-responsive vaccine access during the 2022 global Mpox emergency [Internet]. Available from: https://papers.ssrn.com/abstract=4859548 10.1093/ofid/ofaf053 PMC11949096 40166647

[37] Cascalheira, C. J. et al. Analysis of smartphone text data related to Mpox from a U.S. sample of gay, bisexual, and other men who have sex with men during the 2022 outbreak. *LGBT Health* **10**, 560–5 (2023). 37219872 10.1089/lgbt.2022.0307 PMC10552145

[38] Cascalheira, C. J. et al. An analysis of Mpox communication on Reddit vs Twitter during the 2022 Mpox outbreak. *Sex Res. Soc. Policy*10.1007/s13178-024-01058-4

[39] Parag, K. V. Improved estimation of time-varying reproduction numbers at low case incidence and between epidemic waves. *PLoS Comput. Biol.* **17**, e1009347 (2021). 34492011 10.1371/journal.pcbi.1009347 PMC8448340

[40] Volz, E. M. Complex population dynamics and the coalescent under neutrality. *Genetics* **190**, 187–201 (2012). 22042576 10.1534/genetics.111.134627 PMC3249372

[41] Volz, E. M., Kosakovsky Pond, S. L., Ward, M. J., Leigh Brown, A. J. & Frost, S. D. W. Phylodynamics of infectious disease epidemics. *Genetics* **183**, 1421–30 (2009). 19797047 10.1534/genetics.109.106021 PMC2787429

[42] Li, B. et al. Seasonal variation in gonorrhoea incidence among men who have sex with men. *Sex. Health* **13**, 589–92 (2016). 27712614 10.1071/SH16122

[43] Jackson, K. J. & Santos, G. M. Advertising patterns of internet-based male sex workers who have sex with men (MSMSW): the association between LGBTQIA+ events and advertising for work during the 2022 pride season. *Am. J. Mens. Health* **17**, 15579883231205984 (2023). 37822094 10.1177/15579883231205984 PMC10571702

[44] Elsesser, S. A. et al. Seasons of risk: anticipated behavior on vacation and interest in episodic antiretroviral pre-exposure prophylaxis (PrEP) among a large national sample of U.S. men who have sex with men (MSM). *AIDS Behav.* **20**, 1400–7 (2016). 26538056 10.1007/s10461-015-1238-0 PMC4854804

[45] Shah, A. P., Smolensky, M. H., Burau, K. D., Cech, I. M. & Lai, D. Recent change in the annual pattern of sexually transmitted diseases in the United States. *Chronobiol. Int.* **24**, 947–60 (2007). 17994348 10.1080/07420520701648325

[46] Allan-Blitz, L. T. & Klausner, J. D. Prevalence of mpox immunity among the core group and its potential to prevent future large-scale outbreaks. *Lancet Microbe* **5**, 100957 (2024). 39127055 10.1016/j.lanmic.2024.100957

[47] Phipps, K. et al. Early release - short-lived neutralizing antibody responses to monkeypox virus in smallpox vaccine–naive persons after JYNNEOS vaccination. *Emerg. Infect. Dis. J.* **31**, 2 (2025). Available from: https://www.nc.cdc.gov/eid/article/31/2/24-1300_article 10.3201/eid3102.241300 PMC11845161 39793541

[48] Matusali, G. et al. Mpox immune response elicited by MVA-BN vaccine over 12 months of follow-up. *J. Infect.* **89**, 106309 (2024). 39368640 10.1016/j.jinf.2024.106309

[49] Jeong, Y. D. et al. Modelling the effectiveness of an isolation strategy for managing mpox outbreaks with variable infectiousness profiles. *Nat. Commun.* **15**, 7112 (2024). 39187511 10.1038/s41467-024-51143-w PMC11347573

[50] Chen, F. et al. Intentions of healthcare seeking and self-isolation for MPOX among men who have sex with men in China: a national cross-sectional study. *Emerg. Microbes Infect.* **13**, 2352426 (2024). 10.1080/22221751.2024.2352426 PMC11132697 38713582

[51] Chard, A. N. Risk of Clade II Mpox Associated with Intimate and Nonintimate Close Contact Among Men Who Have Sex with Men and Transgender Adults — United States, August 2022–July 2023. MMWR Morb Mortal Wkly Rep [Internet]. 2024 [cited 2025 Jan 29];73. Available from: https://www.cdc.gov/mmwr/volumes/73/wr/mm7340a2.htm 10.15585/mmwr.mm7340a2 PMC11466375 39388387

[52] O’Toole, Á. et al. Putative APOBEC3 deaminase editing in MPXV as evidence for sustained human transmission since at least 2016 [Internet]. bioRxiv; 2023 [cited 2023 Apr 19]. p. 2023.01.23.525187. Available from: https://www.biorxiv.org/content/10.1101/2023.01.23.525187v1

[53] Sanjuán, R. From molecular genetics to phylodynamics: evolutionary relevance of mutation rates across viruses. *PLOS Pathog.* **8**, e1002685 (2012). 22570614 10.1371/journal.ppat.1002685 PMC3342999

[54] Mpox LA County Department of Public Health. Available from: http://publichealth.lacounty.gov/media/monkeypox/data/index.htm

[55] Zhang, W. et al. Behavior changes influence mpox transmission in the United States, 2022–2023: insights from homogeneous and heterogeneous models. *PNAS Nexus* **4**, pgaf025 (2025). 39925853 10.1093/pnasnexus/pgaf025 PMC11803423

[56] Figgins, M. D. & Bedford, T. Inferring variant-specific effective reproduction numbers from combined case and sequencing data. *eLife* **14**, (2025). 10.7554/eLife.104802 PMC13349379 42423252

[57] Guzzetta, G. et al. Early Estimates of Monkeypox Incubation Period, Generation Time, and Reproduction Number, Italy, May–June 2022 - Volume 28, Number 10—October 2022 - Emerging Infectious Diseases journal - CDC. [cited 2023 Apr 19]; Available from: https://www.nc.cdc.gov/eid/article/28/10/22-1126_article 10.3201/eid2810.221126 PMC9514338 35994726

[58] Ponce, L. et al. Incubation period and serial interval of Mpox in 2022 global outbreak compared with historical estimate. *Emerg. Infect. Dis. J.* **30**, 6 (2024). Available from: https://www.nc.cdc.gov/eid/article/30/6/23-1095_article 10.3201/eid3006.231095 PMC11138990 38781950

[59] Collier, A. risY. et al. Decline of Mpox Antibody responses after modified vaccinia ankara–bavarian nordic vaccination. *JAMA* **332**, 1669–72 (2024). Nov 19. 39361499 10.1001/jama.2024.20951 PMC11581614

[60] USC Center for Advanced Research Computing | Distribution License. Available from: https://www.carc.usc.edu/about/license

[61] Aksamentov, I., Roemer, C., Hodcroft, E. B. & Neher, R. A. Nextclade: clade assignment, mutation calling and quality control for viral genomes. *J. Open Source Softw.* **6**, 3773 (2021).

[62] Minh, B. Q. et al. IQ-TREE 2: new models and efficient methods for phylogenetic inference in the genomic era. *Mol. Biol. Evol.* **37**, 1530–4 (2020). 32011700 10.1093/molbev/msaa015 PMC7182206

[63] Sagulenko, P., Puller, V. & Neher, R. A. TreeTime: maximum-likelihood phylodynamic analysis. *Virus Evol.* **4**, vex042 (2018). 29340210 10.1093/ve/vex042 PMC5758920

[64] Lemey, P., Rambaut, A., Drummond, A. J. & Suchard, M. A. Bayesian phylogeography finds its roots. *PLOS Comput. Biol.* **5**, e1000520 (2009). 19779555 10.1371/journal.pcbi.1000520 PMC2740835

[65] Huddleston, J. et al. Augur: a bioinformatics toolkit for phylogenetic analyses of human pathogens. *J. Open Source Softw.* **6**, 2906 (2021). 34189396 10.21105/joss.02906 PMC8237802

[66] Müller NF, et al. Viral genomes reveal patterns of the SARS-CoV-2 outbreak in Washington State. *Sci. Transl. Med*. 2021 May 26. Available from: https://stm.sciencemag.org/content/13/595/eabf0202 10.1126/scitranslmed.abf0202 PMC8158963 33941621

[67] Paredes, M. I. et al. Local-scale phylodynamics reveal differential community impact of SARS-CoV-2 in a metropolitan US county. *PLOS Pathog.* **20**, e1012117 (2024). 38530853 10.1371/journal.ppat.1012117 PMC10997136

[68] Ferretti, L. et al. Biased estimates of phylogenetic branch lengths resulting from the discretised Gamma model of site rate heterogeneity [Internet]. *bioRxiv* Available from: https://www.biorxiv.org/content/10.1101/2024.08.01.606208v1 10.1093/sysbio/syag037 42161371

[69] Frost, S. D. W. & Volz, E. M. Viral phylodynamics and the search for an ‘effective number of infections. *Philos. Trans. R. Soc. B Biol. Sci.* **365**, 1879–90 (2010). 10.1098/rstb.2010.0060 PMC2880113 20478883

[70] Bouckaert, R. et al. BEAST 2.5: An advanced software platform for Bayesian evolutionary analysis. *PLOS Comput. Biol.* **15**, e1006650 (2019). 30958812 10.1371/journal.pcbi.1006650 PMC6472827

[71] Baele, G., Lemey, P., Rambaut, A. & Suchard, M. A. Adaptive MCMC in Bayesian phylogenetics: an application to analyzing partitioned data in BEAST. *Bioinform. Oxf. Engl.* **33**, 1798–805 (2017). 10.1093/bioinformatics/btx088 PMC6044345 28200071

[72] Müller, N. F. & Bouckaert, R. R. Adaptive metropolis-coupled MCMC for BEAST 2. *PeerJ* **8**, e9473 (2020). 32995072 10.7717/peerj.9473 PMC7501786

[73] Rambaut, A., Drummond, A. J., Xie, D., Baele, G. & Suchard, M. A. Posterior summarization in Bayesian phylogenetics using Tracer 1.7. *Syst. Biol.* **67**, 901–4 (2018). 29718447 10.1093/sysbio/syy032 PMC6101584

[74] Vaughan, T. G. IcyTree: rapid browser-based visualization for phylogenetic trees and networks. *Bioinformatics* **33**, 2392–2394 (2017). 10.1093/bioinformatics/btx155 PMC5860111 28407035

[75] VanderPlas, J. et al. Altair: Interactive statistical visualizations for Python. *J. Open Source Softw.* **3**, 1057 (2018).

[76] Hunter, J. D. Matplotlib: a 2D graphics environment. *Comput Sci. Eng.* **9**, 90–5 (2007).

[77] Waskom, M. L. seaborn: statistical data visualization. *J. Open Source Softw.* **6**, 3021 (2021).

[78] Bedford, T., Cobey, S., Beerli, P. & Pascual, M. Global migration dynamics underlie evolution and persistence of human influenza A (H3N2). *PLOS Pathog.* **6**, e1000918 (2010). 20523898 10.1371/journal.ppat.1000918 PMC2877742

[79] Ma, J. Estimating epidemic exponential growth rate and basic reproduction number. *Infect. Dis. Model* **5**, 129–41 (2020). 31956741 10.1016/j.idm.2019.12.009 PMC6962332

[80] Angelo, K. M. et al. Epidemiological and clinical characteristics of patients with monkeypox in the GeoSentinel Network: a cross-sectional study. *Lancet Infect. Dis.* **23**, 196–206 (2023). 36216018 10.1016/S1473-3099(22)00651-X PMC9546520

[81] Brochero, C. D. et al. Decoding mpox: a systematic review and meta-analysis of the transmission and severity parameters of the 2022–2023 global outbreak. *BMJ Glob. Health* Available from: https://gh.bmj.com/content/10/1/e016906 10.1136/bmjgh-2024-016906 PMC11792283 39890207

[82] Wallinga, J. & Lipsitch, M. How generation intervals shape the relationship between growth rates and reproductive numbers. *Proc. R. Soc. B Biol. Sci.* **274**, 599–604 (2007). 10.1098/rspb.2006.3754 PMC1766383 17476782

[83] Zhang, X. S. et al. Transmission dynamics and effect of control measures on the 2022 outbreak of mpox among gay, bisexual, and other men who have sex with men in England: a mathematical modelling study. *Lancet Infect. Dis.* **24**, 65–74 (2024). 37708908 10.1016/S1473-3099(23)00451-6

[84] WHO. Mpox [Internet]. [cited 2025 Feb 13]. Available from: https://www.who.int/news-room/fact-sheets/detail/mpox

[85] Savinkina et al. Modelling vaccination approaches for mpox containment and mitigation in the Democratic Republic of the Congo. *Lancet Glob. Health* **12**, e1936–44 (2024). 39393385 10.1016/S2214-109X(24)00384-X

[86] Lloyd-Smith, J. O., Schreiber, S. J., Kopp, P. E. & Getz, W. M. Superspreading and the effect of individual variation on disease emergence. *Nature* **438**, 355–9 (2005). 16292310 10.1038/nature04153 PMC7094981

[87] Lemey, P. et al. Untangling introductions and persistence in COVID-19 resurgence in Europe. *Nature* **595**, 713–7 (2021). 34192736 10.1038/s41586-021-03754-2 PMC8324533

[88] Hinch, R. et al. Quantification of the time-varying epidemic growth rate and of the delays between symptom onset and presenting to healthcare for the mpox epidemic in the UK in 2022. *Sci. Rep.* **14**, 19755 (2024). 39187529 10.1038/s41598-024-68154-8 PMC11347625

[89] Rambaut, A. & Grass, N. C. Seq-Gen: an application for the Monte Carlo simulation of DNA sequence evolution along phylogenetic trees. *Bioinformatics* **13**, 235–8 (1997). 10.1093/bioinformatics/13.3.235 9183526

