# Supplementary material for: Viral introductions and return to baseline sexual behaviors maintain low-level mpox incidence in Los Angeles
Source: Nat Commun. 2026 Apr 17;17:5376. doi: 10.1038/s41467-026-71993-w (PMC13276399; doi:10.1038/s41467-026-71993-w)
Supplement: Supplementary file 1 — Supplementary Information [file 41467_2026_71993_MOESM1_ESM.pdf]

Supplementary Material:

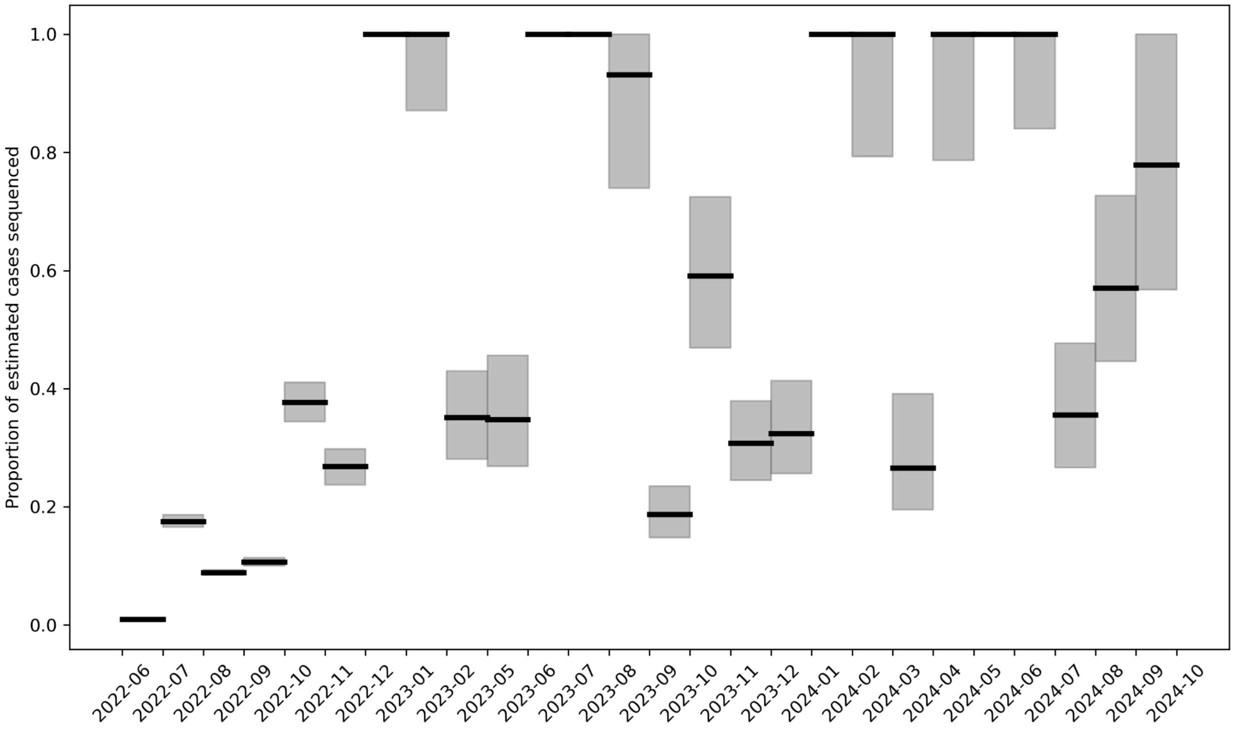

Figure S1: Estimated proportion of mpox cases sequenced in Los Angeles County (LAC). The proportion of cases sequenced was calculated by dividing the total number of mpox sequences from LAC found on GenBank by the monthly mpox incidence estimated from case counts using a renewal equation framework. The dark black horizontal line refers to the median estimates with the grey bars representing the 95% CI based on uncertainty in the incidence estimates. Months where the estimated proportion was greater than 100% (due to uncertainty incidence estimation due to low case counts or sample collection at dates different than diagnosis) were bounded at 100%.

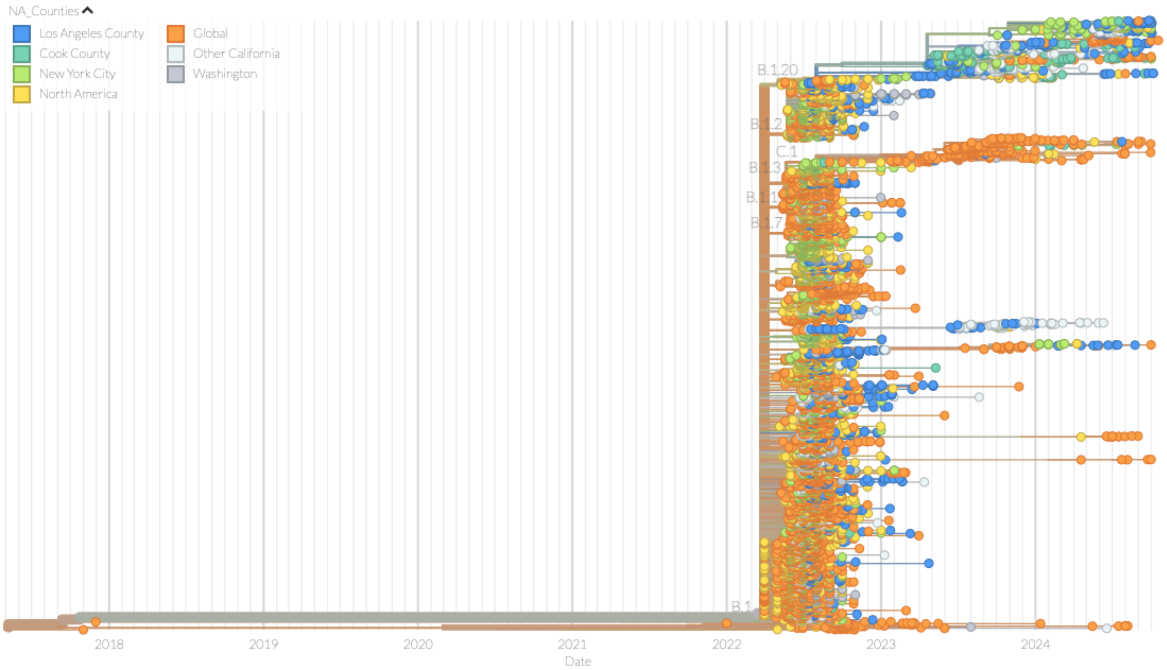

Figure S2: Time-resolved maximum likelihood phylogenies for mpox clade IIb sample collection dates between January 1 2022 and December 12, 2024 Tip color represents the focus areas in North America with high sequencing effort. Branches are colored based on inferred ancestry. The full tree can be explored interactively at https://nextstrain.org/groups/blab/mpox-la/allcladeIIseqs?c=focus_areas

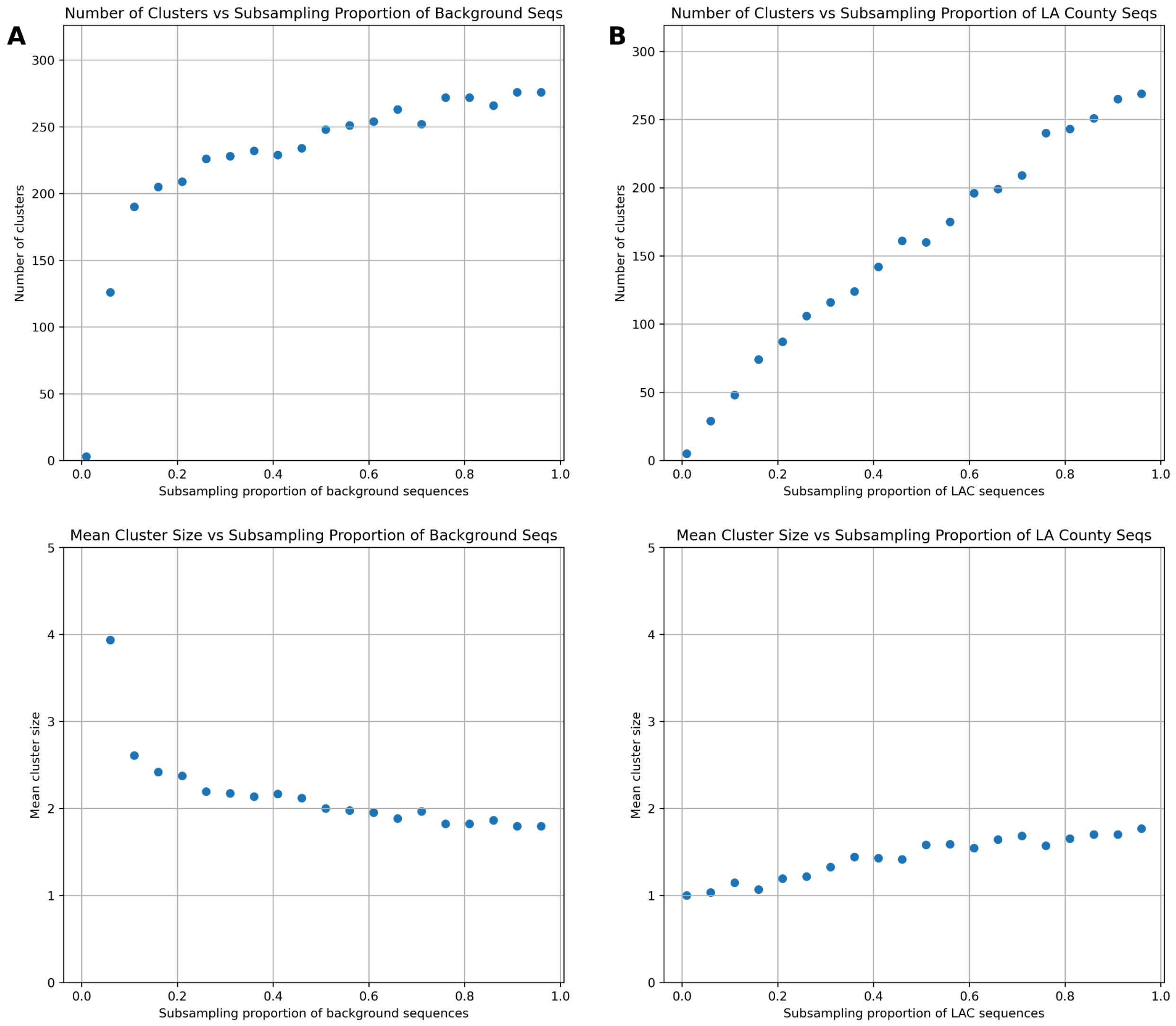

Figure S3: The impact of subsampling on the number and size of transmission clusters identified. We randomly subsampled different proportions of background sequences **(A)** and sequences from LA **(B)** and ran our clustering algorithm to show the impact of increasing the proportion sequences relative to the full dataset on the total number of clusters identified (Top row) and the mean size of those clusters (bottom row). LA = Los Angeles

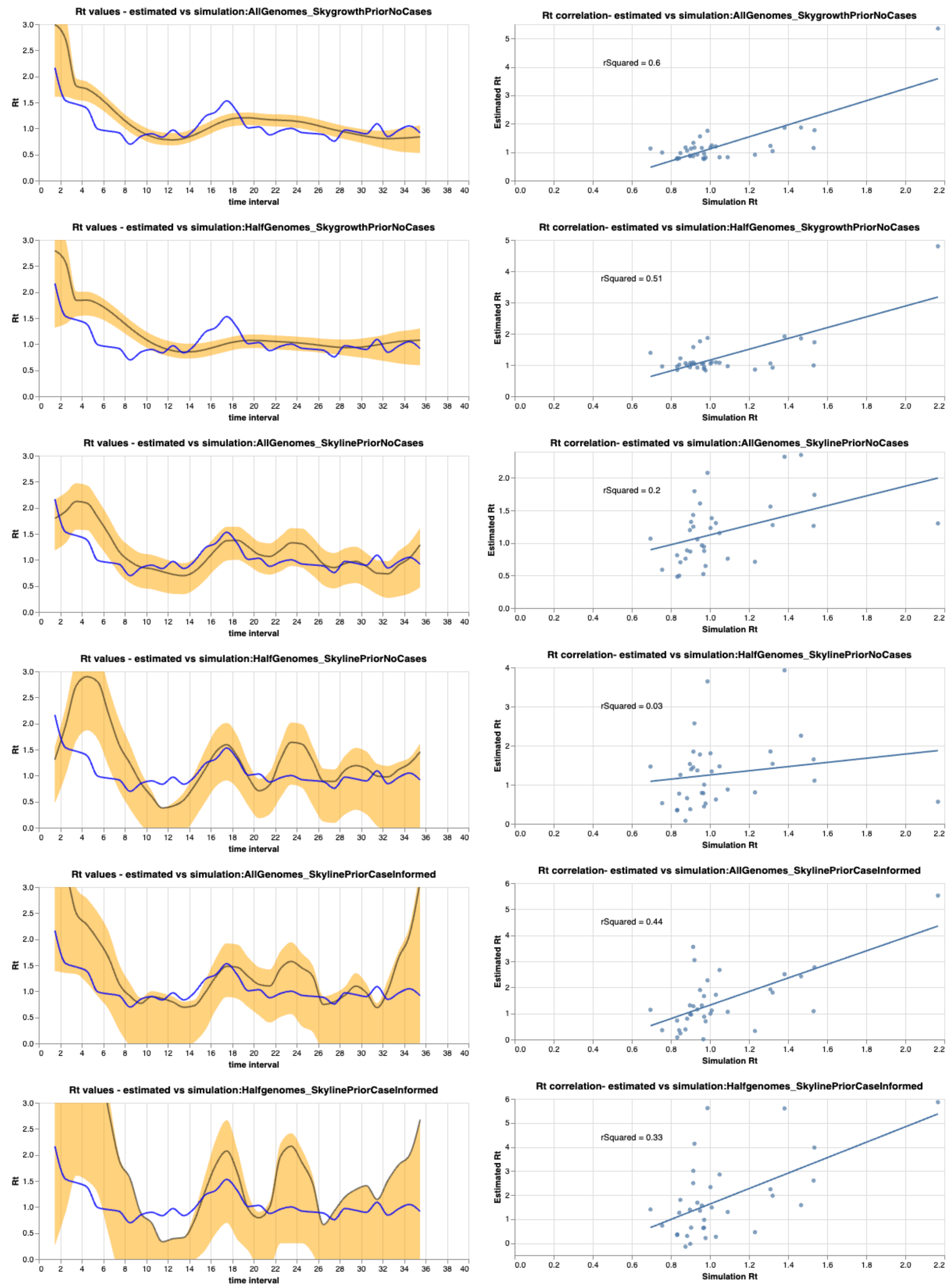

Figure S4: Estimation of *Rt* from simulations. We tested the ability of our multitree coalescent approach under different Ne priors to correctly estimate *Rt* from simulations. To do so, we simulated a

local outbreak using a constant rate of introduction. We then sampled cases based on the estimated time to seek care during the 2022 mpox epidemic, subsampled, simulated genetic sequences, and then used the local transmission cluster to estimate Rt. The left column shows the true *Rt* in blue with the estimated *Rt* in yellow (showing the 95% HPD intervals) with the grey line representing the median estimate. The right column shows the correlation between simulated and the estimated *Rt* with a linear regression fit and R^2 calculated. The first two rows represent a skygrowth prior on growth rate, the second two represent a skyline prior on the Ne without cases, and the bottom set of two represent the main analysis of a skyline prior on the Ne informed by mpox cases. For each set of priors, the top analysis represents 100% of the sampled genomes used while the bottom analysis represents only 50% of the genomes used. Estimates were smoothed using a 14 day rolling average. Rt = effective reproductive number, Ne = effective population size.

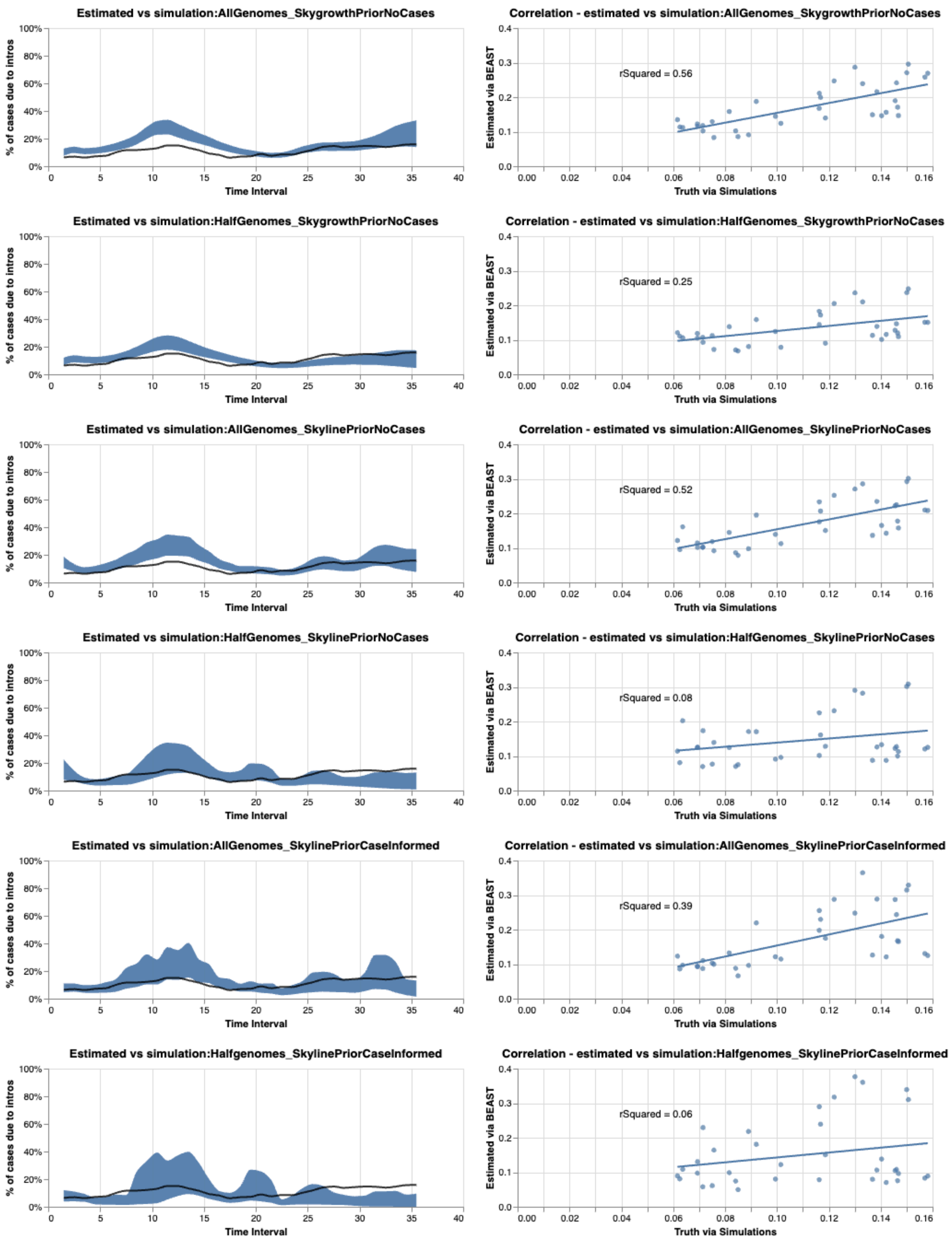

Figure S5: Estimation of percentage of cases due to introductions from simulations. We tested the ability of our multitree coalescent approach under different Ne priors to correctly estimate the percentage of cases due to introductions from simulations. To do so, we simulated a local outbreak using a constant

rate of introduction. We then sampled cases based on the estimated time to seek care during the 2022 mpox epidemic, subsampled, simulated genetic sequences, and then used the local transmission cluster to estimate percentage The left column shows the true percentage in black with the estimated percentage in blue (showing the 95% HPD intervals). The right column shows the correlation between simulated and the estimated percentage with a linear regression fit and R^2 calculated. The first two rows represent a skygrowth prior on growth rate, the second two represent a skyline prior on the Ne without cases, and the bottom set of two represent the main analysis of a skyline prior on the Ne informed by mpox cases. For each set of priors, the top analysis represents 100% of the sampled genomes used while the bottom analysis represents only 50% of the genomes used. Estimates were smoothed using a 14 day rolling average. Ne = effective population size.

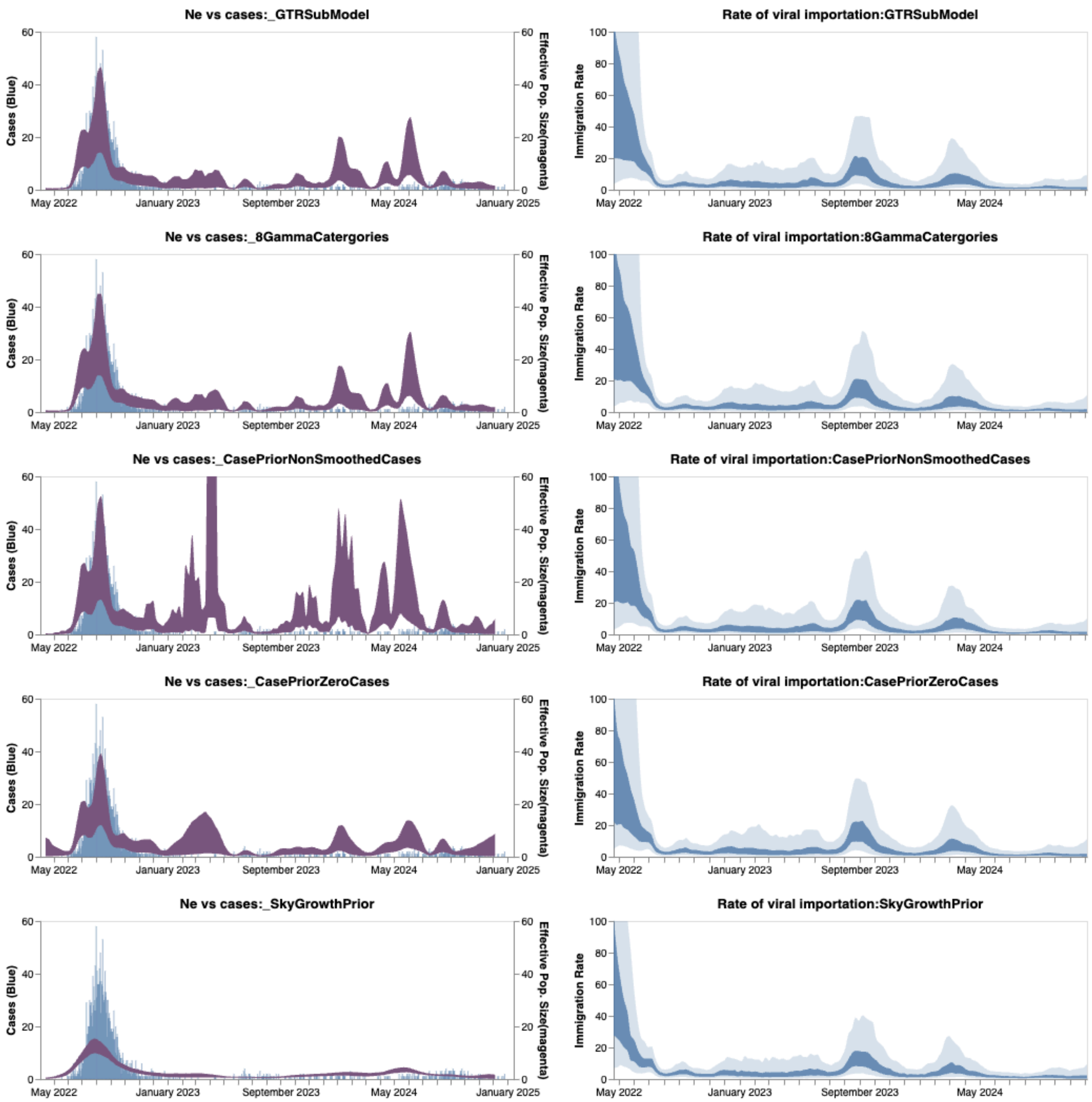

Figure S6: Sensitivity analysis of phylodynamic results: We tested the robustness of our phylodynamic results by repeating our main analyses under different model specifications. The left column shows the empirical case counts in blue and the estimated effective population size (Ne) in magenta (50% HPD). The right columns the inferred immigration rate of mpox into Los Angeles County, with the dark blue band representing the 50% HPD and the lighter blue representing the 95% HPD. The first row represents the same case-informed estimates as our main result but with a GTR substitution model instead of HKY. Second row represents 8 category discretization of the gamma distribution prior instead of 4 categories. The third row is our main model but without the cases being smoothed prior to being inputted into the model. The fourth row is a skyline prior represented by having zero case information in the case prior. The final row is a skygrowth prior with no case information instead of the skyline case-informed prior. Ne = effective population size.

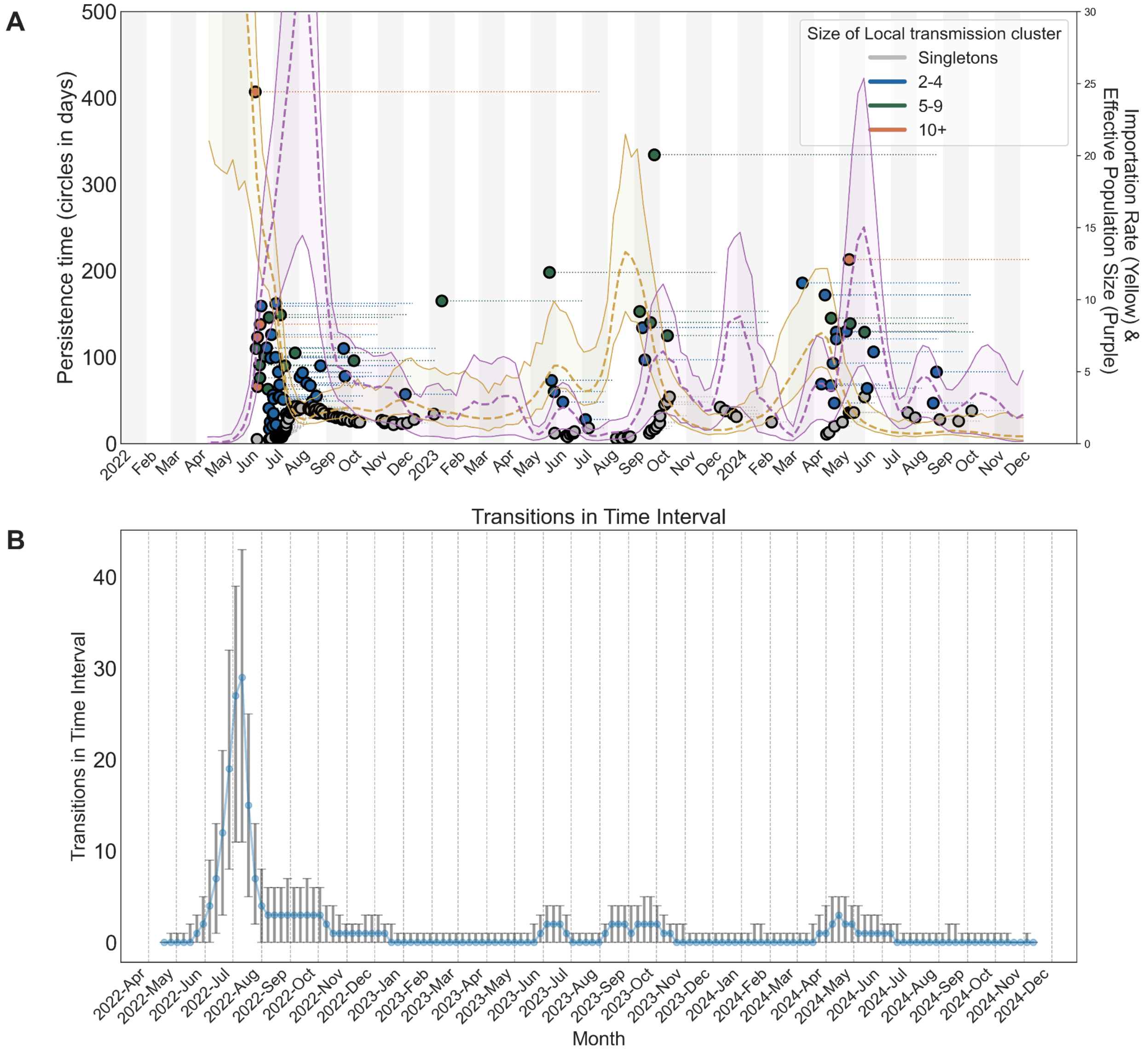

Figure S7: Mpox importation dynamics in LA County estimated via Bayesian Phylodynamics. Panel **A** shows the persistence time of each identified local outbreak cluster according to the date of its inferred introduction time. Each dot represents an inferred introduction into LA County, the radius of the dot is proportional to the size of the resulting transmission cluster. The yellow streamgraph is the rate of introduction (events/lineage/year) into LA county and the purple streamgraph represents the estimated effective population size, both inferred by our phylodynamic model. The dashed line represents the median and the bands represent the 95% HPD. Panel **B** shows the absolute median number of viral introductions inferred via our phylodynamic model for each week that was calculated by analyzing the entire posterior set of phylogenetic trees after burn in. The error bars represent the 95% HPD and these estimates were used to parameterize our microsimulation model.

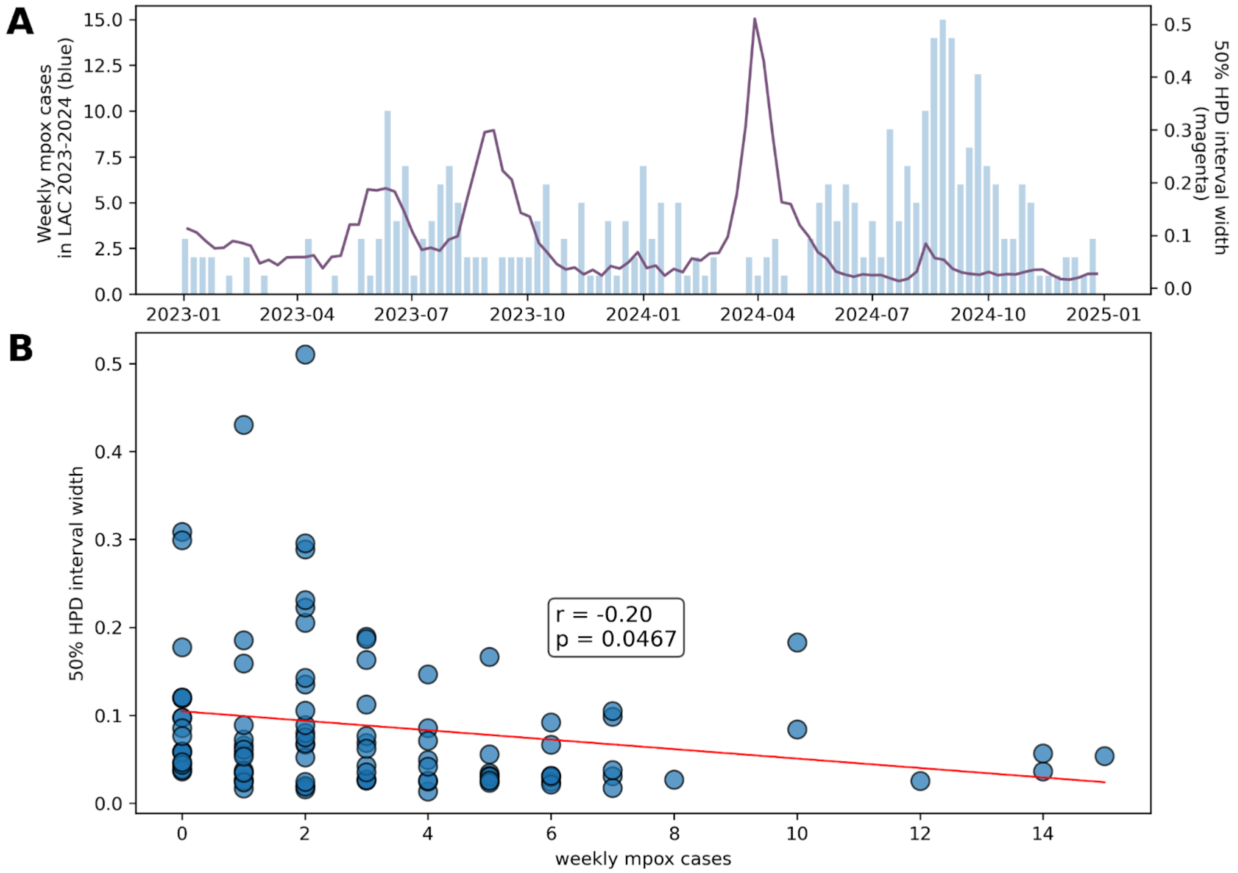

Figure S8: Relationship between uncertainty in percentage of cases due to introductions and case counts. Panel **A** plots the weekly case counts in Los Angeles County from March 2023 through December 2024 in light blue. The purple line denotes the width of the 50% HPD interval found for the estimated percentage of cases due to introductions found in Fig 3D as a measure of uncertainty. Panel **B** shows the relationship between the weekly number of mpox cases in LAC and the width of the same 50% HPD interval. The red line is a linear regression of the scatterplot with the associated Pearson correlation coefficient and p value. HPD = Highest posterior density.

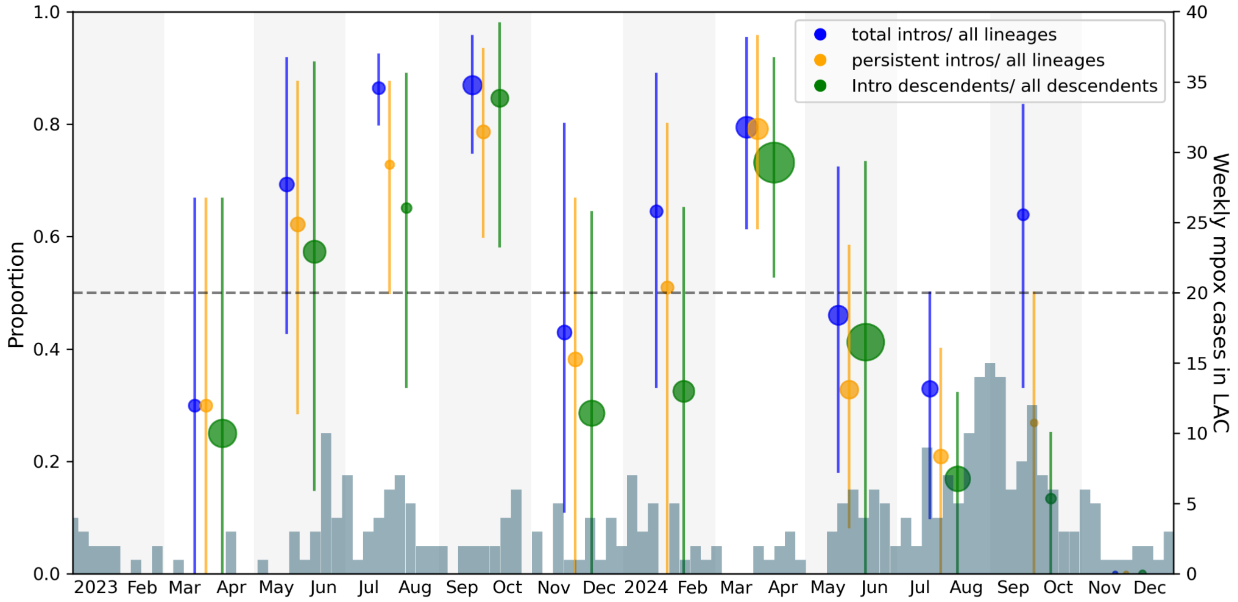

Figure S9: Posterior estimates of relative impact of introductions in Los Angeles County (LAC) every two months from March 2023- December 2024. For every two months from March 1, 2023 through December 12, 2024 (denoted by alternating white and light gray vertical stripes), we report three posterior means and 95% HPD intervals for the relative impact of introductions in LAC estimated using the posterior set of phylogenetic trees from our unstructured coalescent model: (**Blue)** the proportion of unique introductions in the time period over the total number of unique persisting lineages and unique introductions; **(orange)** the proportion of unique introductions whose downstream transmission chains persisted at least until the end of that time period over the total number of persistent introductions and ongoing lineages; and **(green)** the proportion of descendant lineages from these unique introduction events over the total number of descendants circulating by the end of the two month time period. The points are scaled proportional to the total number of lineages or descendants in each time period that respectively contributed to the proportion. The gray bars represent the weekly cases in LAC. Given that our most recent sequence was on Dec 12th 2024, no viral introductions were found in Nov-Dec 2024 as more samples and time are needed to observe successful introductions.

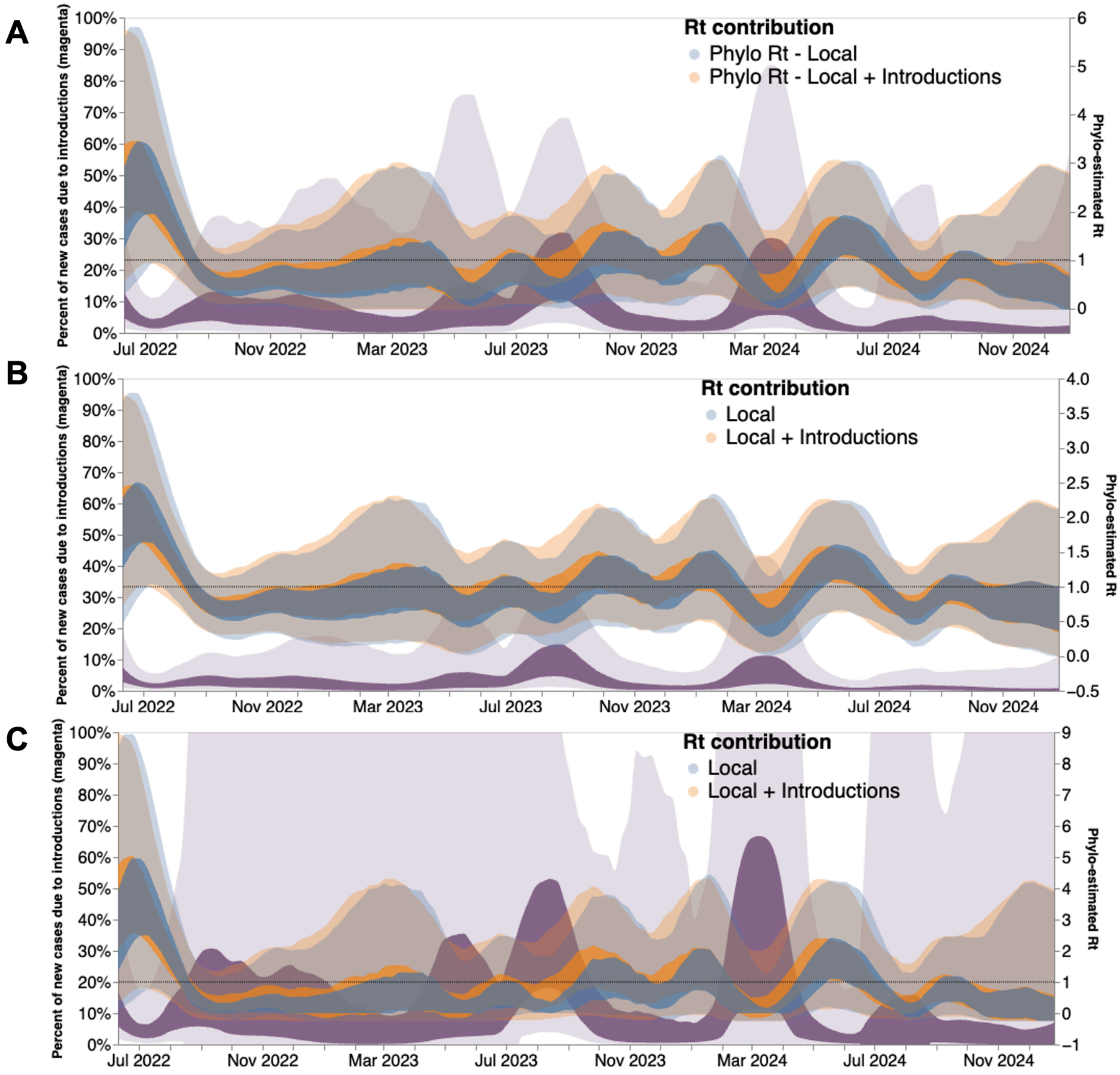

Figure S10: Percentage of new cases due to introductions and *Rt* with infectious period of (A) 10.9, (B) 4.5 and (C) 21 days. The inner area denotes the 50% HPD interval, and the outer area denotes the 95% HPD interval. The blue and orange bands lines represent estimates of *Rt* highlighting the contribution of local transmission only (blue) as well as that of viral introductions (orange). Dashed line highlights an *Rt* value of 1. *Rt* estimates were smoothed using a 14-day rolling average. Rt = effective reproductive number.

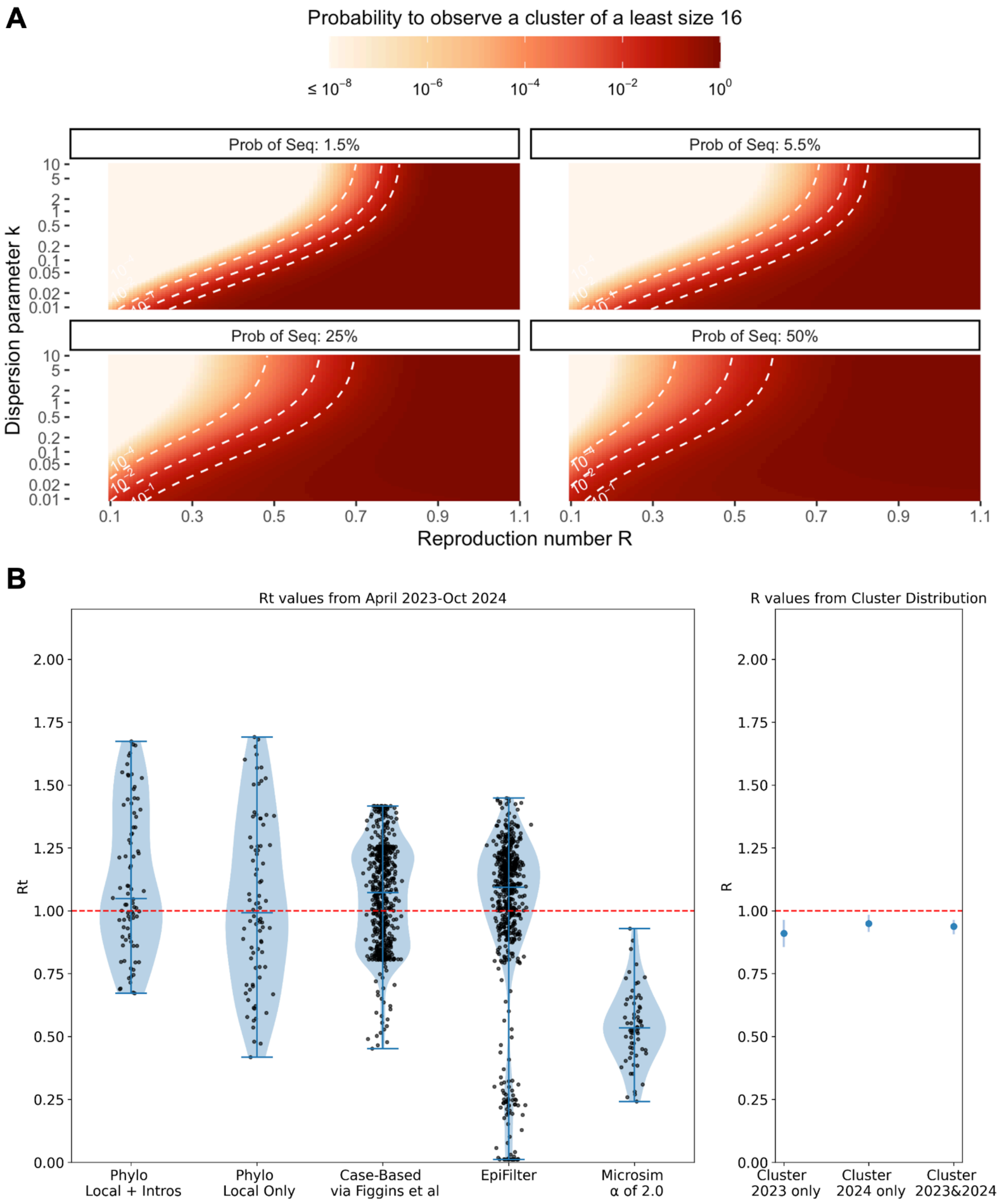

Figure S11: Reproductive number estimation for LAC from March 2023 through October 2024. A. Probability to observe a cluster of size 16 among 64 clusters as a function of the reproduction number R and the dispersion parameter k assuming 1.5%, 5.5%, 25%, and 50% of infections are sequenced. The dotted white lines correspond to contour lines for probabilities of 10−4, 10−2, and 10−1. **B.** The mean estimates of *Rt* (left) or R (right) for mpox showing the spread via a violin plot with the extremes and the median highlighted by the darker blue horizontal lines for diverse methodologies. The left panel plots the spread of weekly *Rt* estimations while the right panel shows the estimates of R with 95% CIs found from the distribution of cluster sizes for either 2023, 2024, or both years combined. The x axis of the left panel shows the methodology used and the dashed red line denotes an R or *Rt* of 1. Rt = effective reproductive number

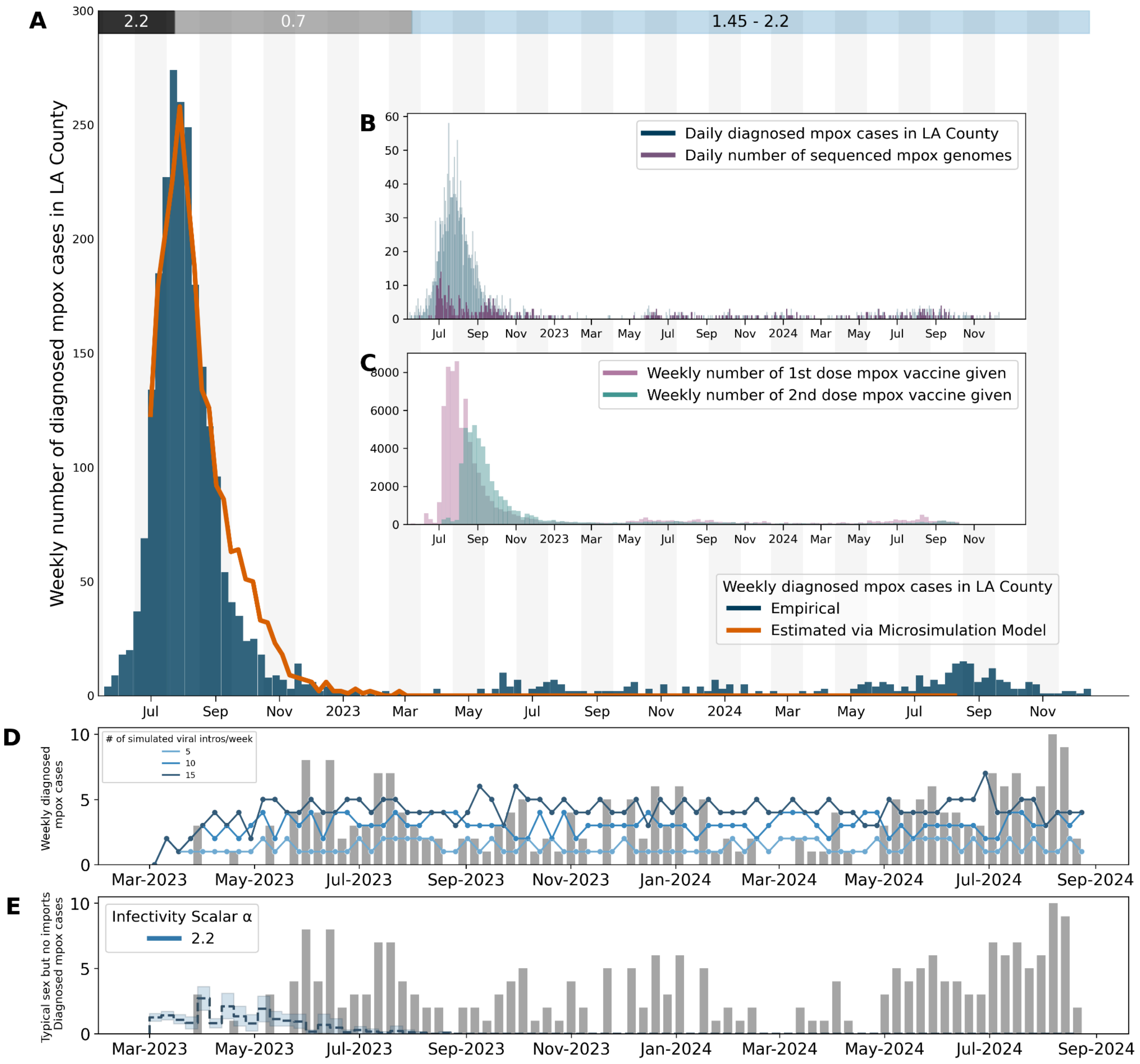

Figure S12: Infectivity Scalar (α) during the 2022 mpox outbreak in Los Angeles County. (A) Main figure shows the weekly number of diagnosed mpox cases in LA County from June 2022 through December 2024 (blue) with the number of diagnosed mpox cases simulated via our microsimulation model without viral importations overlaid in orange. The bars in the top of the figure are a visual representation of the periods of time for which α was calibrated. Panel **B** shows the daily number of diagnosed mpox cases (blue) with the daily number of mpox sequences collected in LAC overlaid (purple) from June 2022 to December 2024. Panel **C** shows the weekly number of mpox vaccinations that were administered in Los Angeles County from June 2022 to October 2024 divided between the number of first doses (pink) and second doses (teal) given. The grey bars represent the initial model calibration for the epidemic period and the blue bar shows the period of interest for this study. **(D)** Scenario analysis of the impact of a constant force of viral introductions on our microsimulation model from March 2023 to September 2024. The empirical mpox case counts are represented by the gray bar chart while the simulated cases are represented by the blue point and line charts with an increasing number of viral importations per week. Panel **E** represents the counterfactual scenario of a complete revision to baseline sexual activity in March 2023 (represented by an α of 2.2) but without any introductions into the model

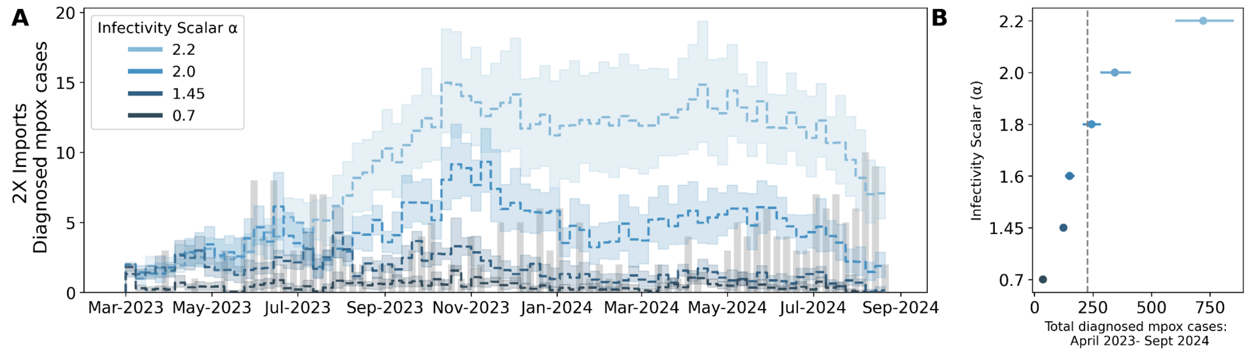

Figure S13: Infectivity Scalar α with twice as many phylodynamics-informed viral introductions. To test the impact of underestimating the number of viral introductions into LAC, we doubled the number of introductions (**A**), reran our microsimulation model, and explored the Infectivity Scalar α that best explains the empirical weekly number of diagnosed mpox cases (gray bars). Line graphs represent the mean weekly number of mpox diagnoses simulated using increasing α. Each weekly estimate represents the average of 10 independent iterations of our model. The dashed line in **B** represents the total empirical number of diagnosed mpox cases from April 2023 through September 2024. For **all panels**, we calculate the uncertainty of our microsimulation results via bootstrapping with 500 samples to estimate 95% uncertainty intervals for each weekly simulated estimate.

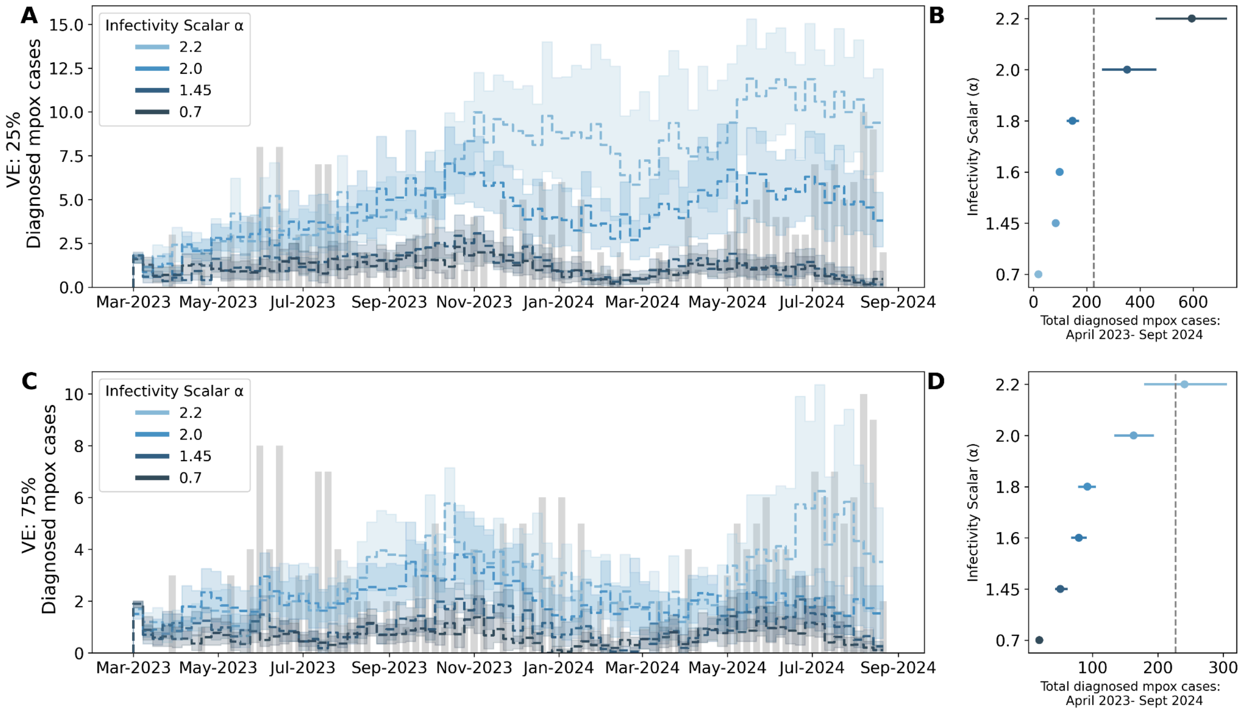

Figure S14: Sensitivity analysis of the impact of vaccine immune waning on α calibration. Here we repeat the analysis in Main Figure 5A-B, with the vaccine effectiveness against infection declining to 25% **(A-B)** and 75% **(C-D)** after one year. Line graphs **(A)** represent the mean weekly number of mpox diagnoses simulated using increasing α. The dashed line in **B** represents the total empirical number of diagnosed mpox cases from April 2023 through September 2024. For **all panels**, we calculate the uncertainty of our microsimulation results via bootstrapping with 500 samples to estimate 95% uncertainty intervals for each weekly simulated estimate.

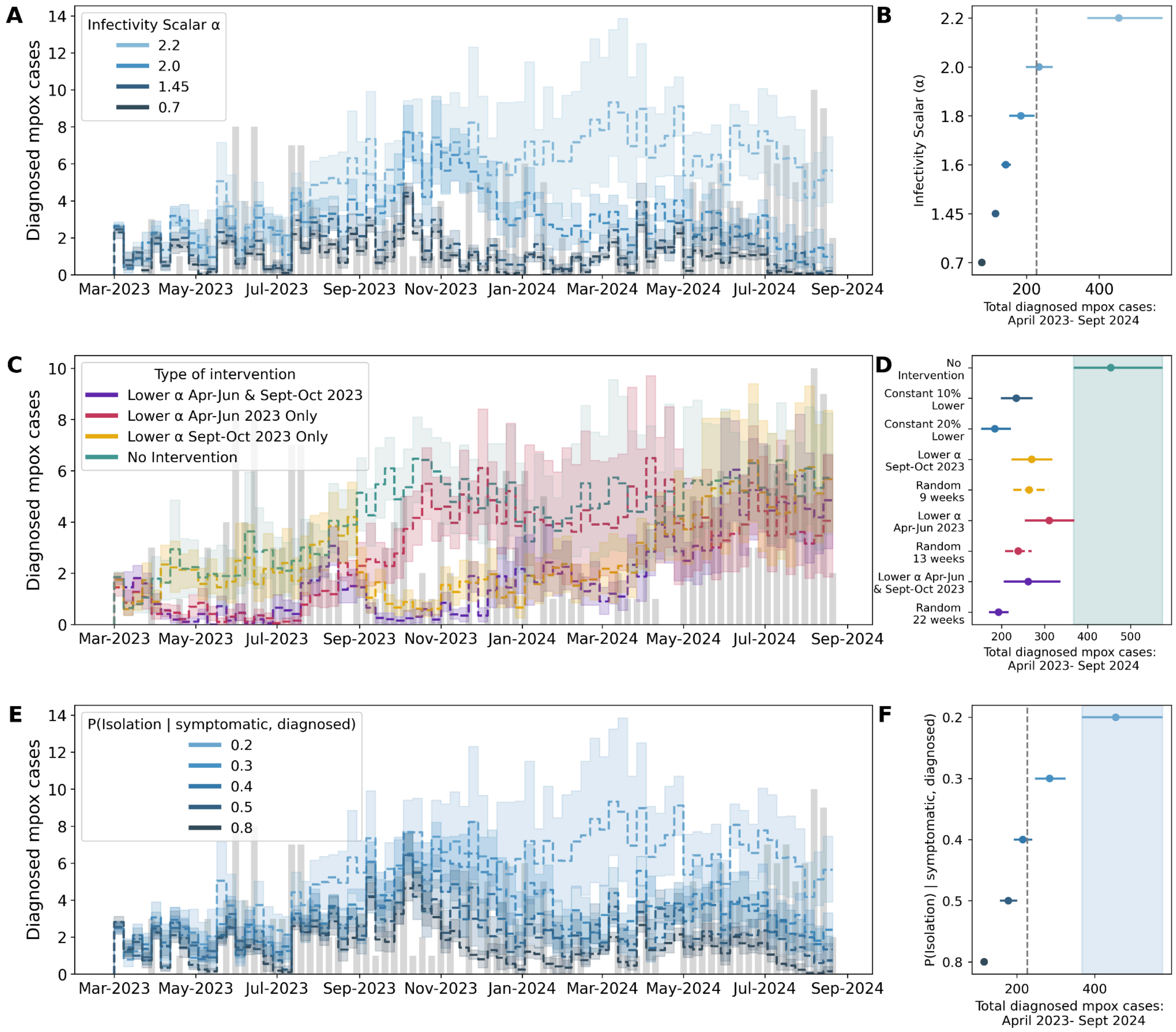

Figure S15: Inputting importations as symptomatic but undiagnosed. Here we repeat the analysis in Main Figure 5 but rather than assume that viral introductions enter the model as symptomatic and diagnosed, they enter as symptomatic and undiagnosed, allowing them to contribute longer to secondary infections before possibly becoming diagnosed. Panels **A** and **B** represents an exploration of the Infectivity Scalar α parameter that best explains the empirical weekly number of diagnosed mpox cases (gray bars). Line graphs represent the mean weekly number of mpox diagnoses simulated using increasing α. Panels **C** and **D** repeat the different counterfactual scenarios of public health interventions during specific time periods represented by lowering the α to 0.7 while keeping the α at 2.2 during the remaining time. The bold yellow, red, and purple lines represent the simulated weekly number of diagnosed mpox cases under phylodynamic-informed interventions. The black line represents a 50% reduction in introductions. In **D,** we also compared the impact of lowering the α for the same random number of weeks as each specified intervention as well as the impact of constantly lowering the α throughout the entire time period by about 10% and 20% to simulate a constant low-effectivity intervention. The green area represents the upper and lower bounds of the “No Intervention” scenario. We also tested the effect of increasing the probability of isolating upon a symptomatic individual receiving a positive mpox diagnosis on the simulated number of diagnosed mpox cases **(E, F).** In **F,** the light blue area represents the bounds of the base model scenario with an α of 2.2. In **A, C, E**, the grey bars

represent the empirical number of mpox diagnoses in LAC. For **B,** the dashed line represents the total empirical number of diagnosed mpox cases from April 2023 through September 2024. For **all panels**, we calculate the uncertainty of our microsimulation results via bootstrapping with 500 samples to estimate 95% uncertainty intervals for each weekly simulated estimate.

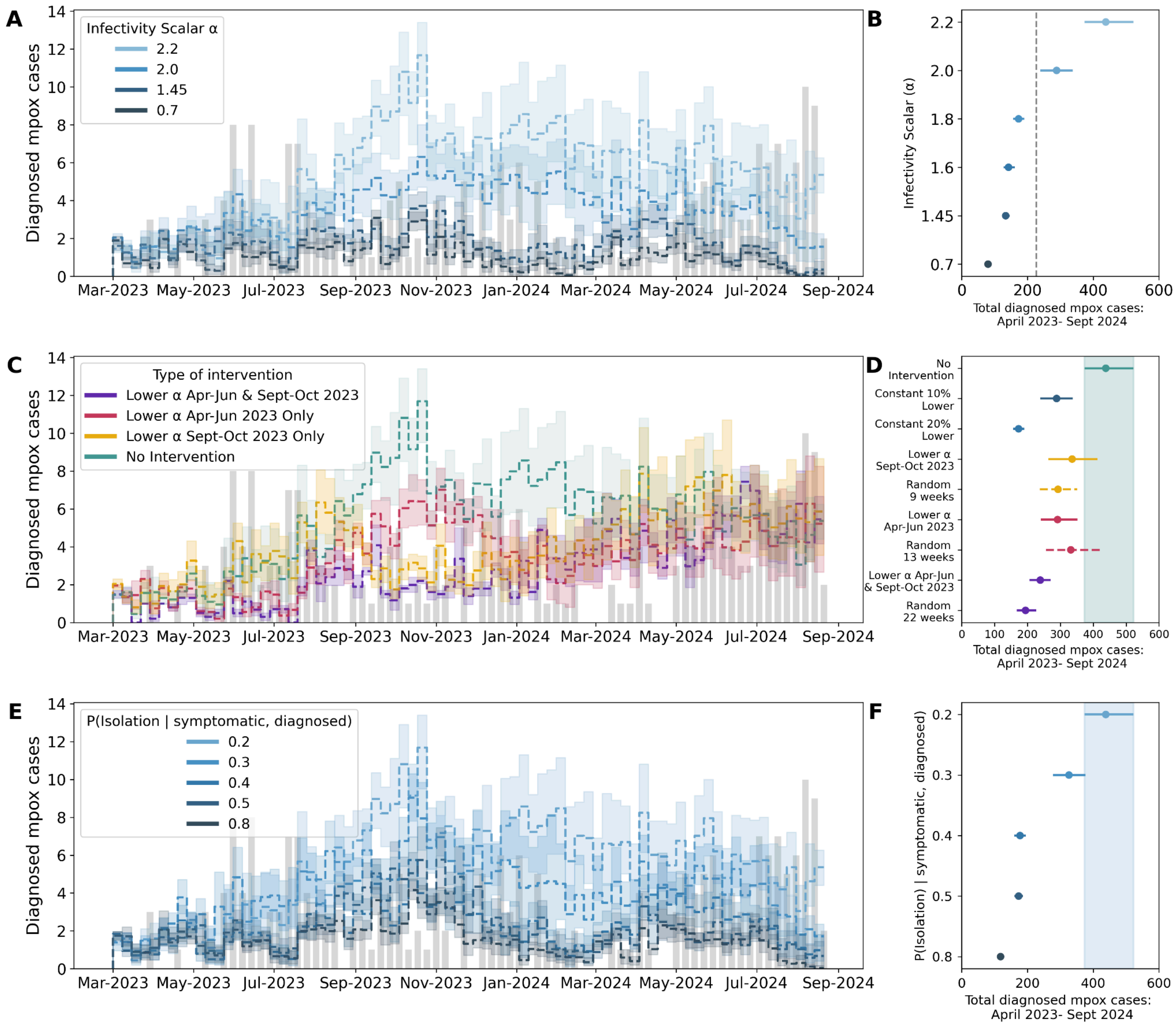

Figure S16: Inputting importations as asymptomatic and undiagnosed. Here we repeat the analysis in Main Figure 5 but rather than assume that viral introductions enter the model as symptomatic and diagnosed, they enter as asymptomatic and undiagnosed, allowing them to contribute longer to secondary infections and transition into being both symptomatic and diagnosed.Panels **A** and **B** represents an exploration of the Infectivity Scalar α parameter that best explains the empirical weekly number of diagnosed mpox cases (gray bars). Line graphs represent the mean weekly number of mpox diagnoses simulated using increasing α. Panels **C** and **D** repeat the different counterfactual scenarios of public health interventions during specific time periods represented by lowering the α to 0.7 while keeping the α at 2.2 during the remaining time. The bold yellow, red, and purple lines represent the simulated weekly number of diagnosed mpox cases under phylodynamic-informed interventions. The black line represents a 50% reduction in introductions. In **D,** we also compared the impact of lowering the α for the same random number of weeks as each specified intervention as well as the impact of constantly lowering the α throughout the entire time period by about 10% and 20% to simulate a constant low-effectivity intervention. The green area represents the upper and lower bounds of the “No Intervention” scenario. We also tested the effect of increasing the probability of isolating upon a symptomatic individual receiving a positive mpox diagnosis on the simulated number of diagnosed mpox cases **(E, F).** In **F,** the light blue area represents the bounds of the base model scenario with an α of 2.2.

In **A, C, E**, the grey bars represent the empirical number of mpox diagnoses in LAC. For **B,** the dashed line represents the total empirical number of diagnosed mpox cases from April 2023 through September 2024. For **all panels**, we calculate the uncertainty of our microsimulation results via bootstrapping with 500 samples to estimate 95% uncertainty intervals for each weekly simulated estimate.

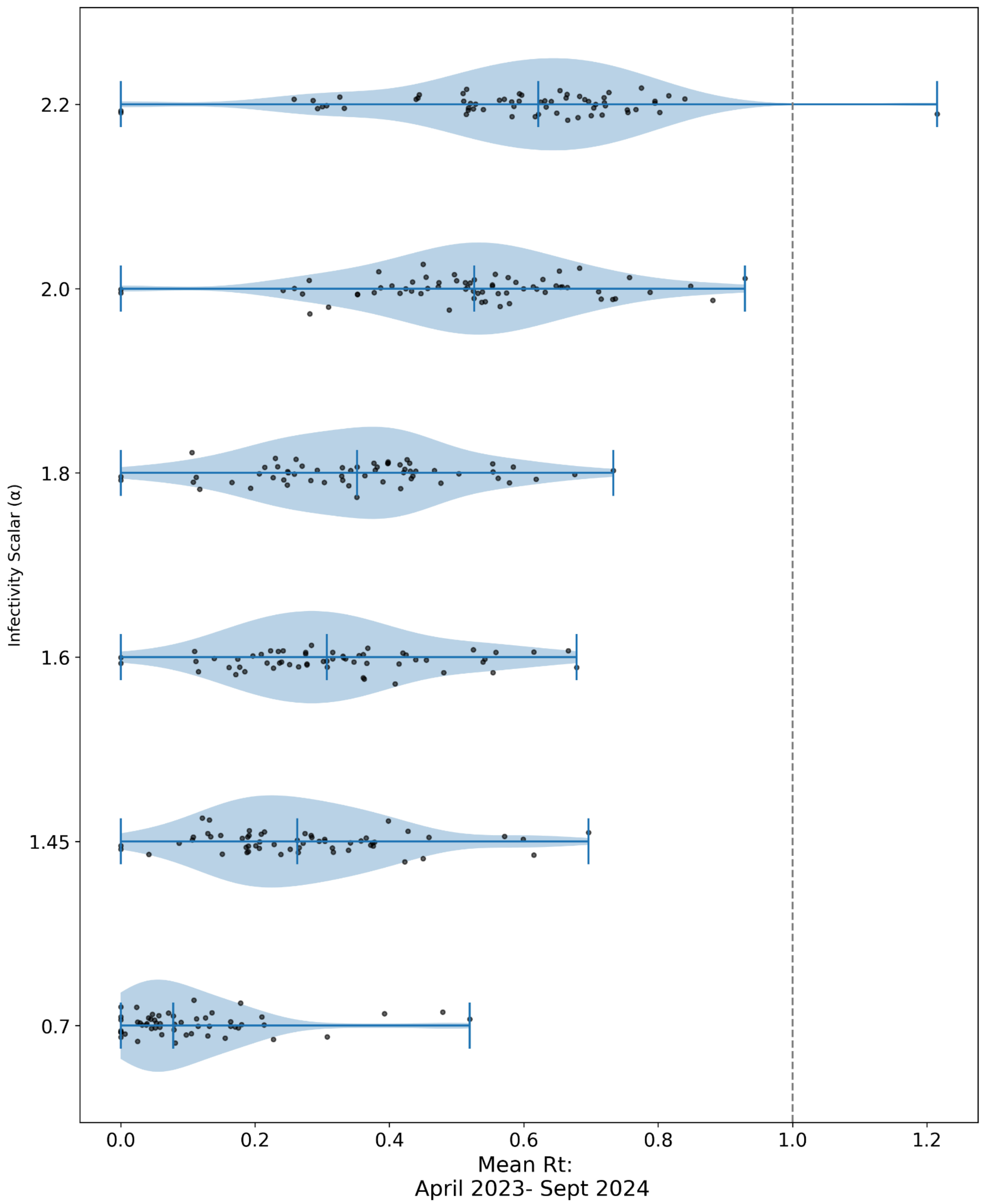

Figure S17: Mean *Rt* estimates for varying values of the Infectivity scalar via microsimulation for LAC from March 2023 through October 2024. The mean estimates of *Rt* for mpox for increasing values of α showing the spread via a violin plot with the extremes and the median highlighted by the darker blue horizontal lines. The dashed black line denotes an *Rt* of 1. *Rt* was estimated by tracking the average weekly number of secondary infections per infected individual multiplied by the time that individuals remain infectious.

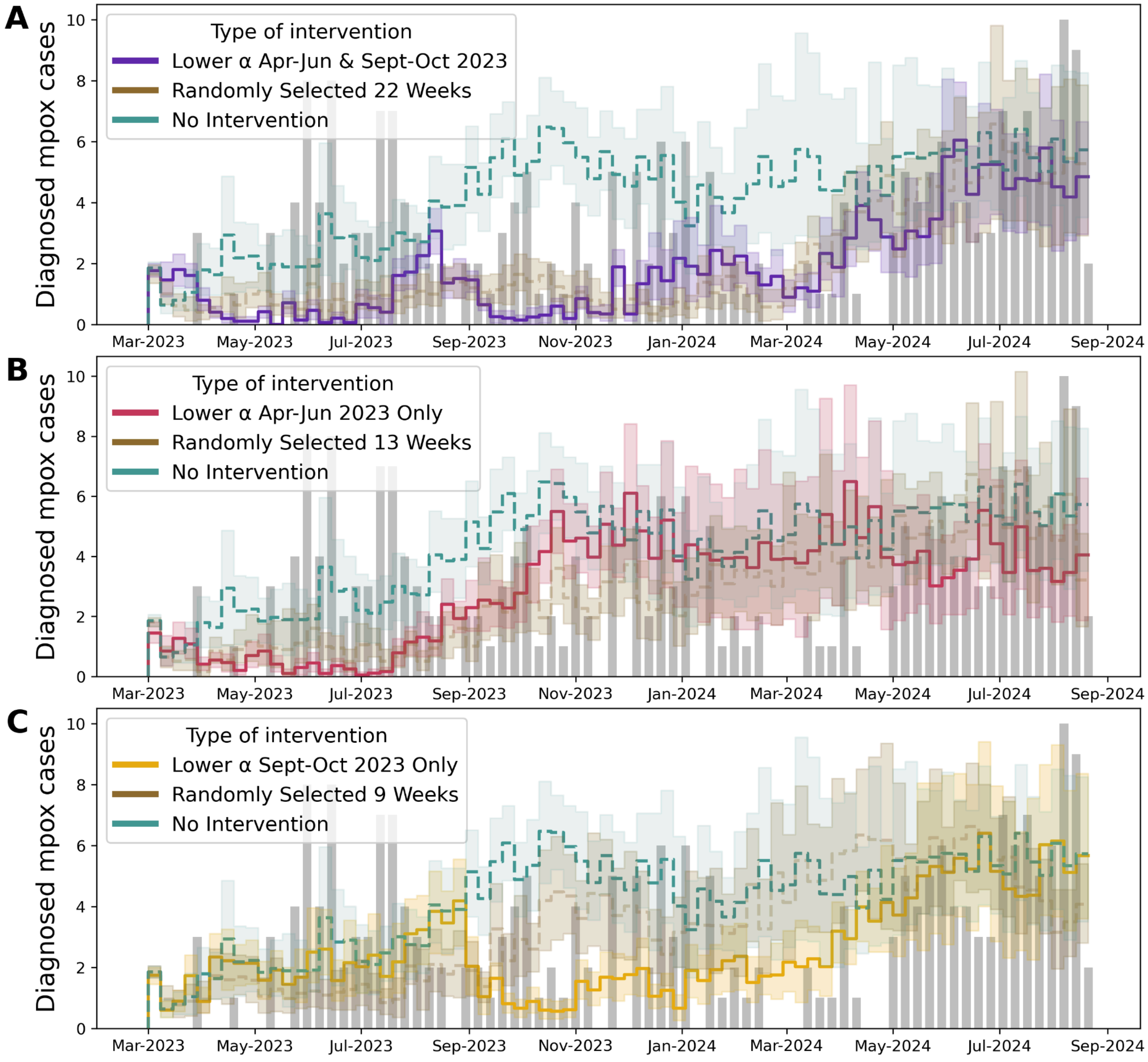

Figure S18: Factors maintaining mpox prevalence and modeling counterfactual public health interventions with statistical tests. (A-C) Given the non-constant pattern of viral introductions seen in the phylodynamic analysis, we tested different counterfactual scenarios of public health interventions during specific time periods represented by lowering the α to 0.7 while keeping the α at 2.2 during the remaining time. The bold yellow, red, and purple solid lines represent the simulated weekly number of diagnosed mpox cases under phylodynamic-informed interventions. To test for non-specific effects, we also reran our microsimulation model by randomly selecting the same number of weeks in 2023 as our phylodynamics-informed interventions to lower the α to 0.7 (brown dashed lines) as well as a simulation without any interventions. (green dashed lines) In all plots, the grey bars represent the empirical number of mpox diagnoses in LAC.

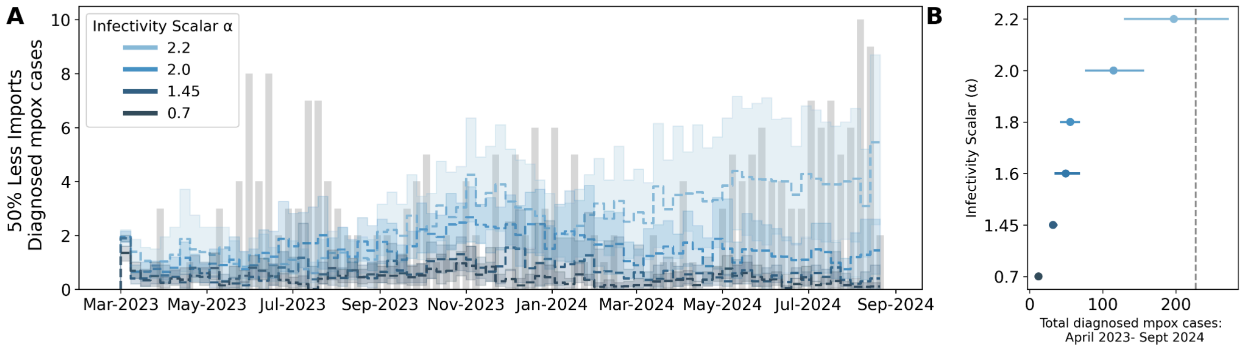

Figure S19: Infectivity Scalar α with half as many phylodynamics-informed viral introductions. To test the impact of interventions that targeted introductions, we halved the number of introductions, reran our microsimulation model, and, in Panel **A**, explored the Infectivity Scalar α that best explains the empirical weekly number of diagnosed mpox cases (gray bars). Line graphs represent the mean weekly number of mpox diagnoses simulated using increasing . Each weekly estimate represents the average of 10 independent iterations of our model. The dashed line in **B** represents the total empirical number of diagnosed mpox cases from April 2023 through September 2024. For **all panels**, we calculate the uncertainty of our microsimulation results via bootstrapping with 500 samples to estimate 95% uncertainty intervals for each weekly simulated estimate.

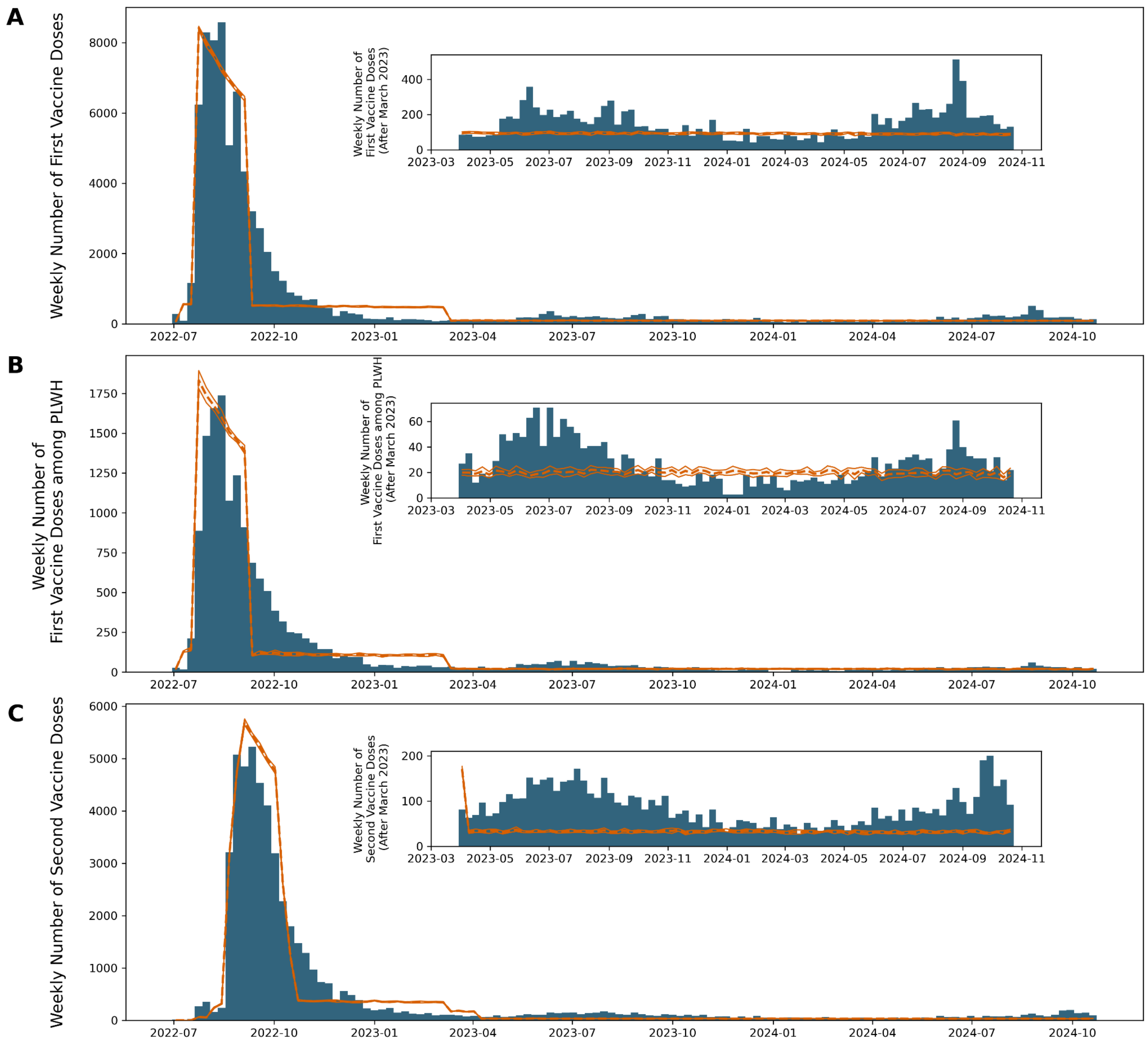

Figure S20: Vaccination validation of microsimulation model. All three panels represent the comparison between the empirical number of vaccination doses given (dark blue) and the number of doses administered as simulated by our model (orange). The dashed orange line represents the mean; the bands represent the 95% uncertainty interval calculated via bootstrapping. The inset graphs for each panel represents the same data but only after March 2023 to allow for better visualization of smaller numbers. Panel **A** represents the comparison of the number of first doses of the mpox vaccine given, panel **B** is for the number of first doses among people with HIV (PWH), and panel **C** is the number of second doses given.

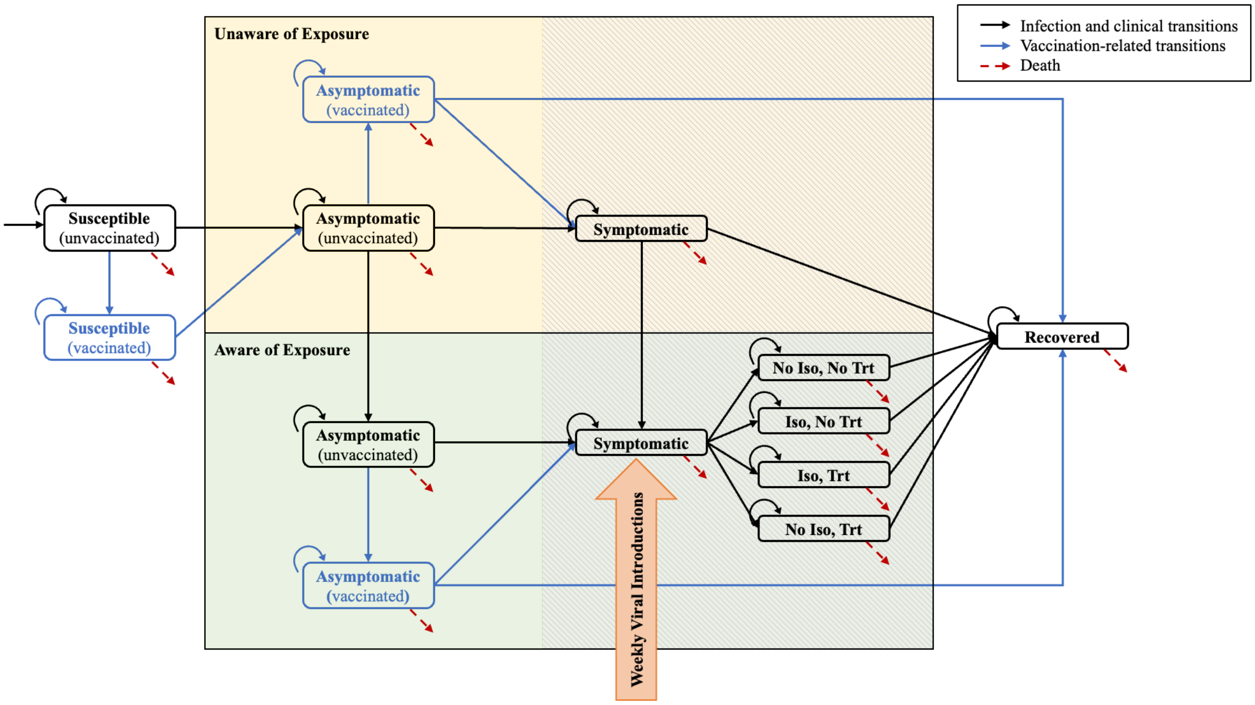

Figure S21. Schematic of the individual-based microsimulation model for mpox transmission dynamics in LAC, incorporating phylodynamics-informed viral introductions. The model simulates weekly transitions through infection, vaccination, diagnosis, isolation, treatment, and recovery. Individuals enter as susceptible and, upon infection, progress through an asymptomatic stage before becoming symptomatic. Transmission occurs when symptomatic individuals expose susceptible partners through sexual contact. Awareness status (yellow vs. green regions) drives care-seeking behaviors. Individuals Aware of Exposure (green) actively seek protection; they have a high probability of receiving vaccine post-exposure prophylaxis (PEP) while asymptomatic, transitioning directly to recovery (blue arrow). Conversely, those Unaware of Exposure (yellow) receive vaccination at the same rate as susceptible individuals. Upon developing symptoms, all individuals enter the symptomatic phase (grey-patterned region). Within this phase, aware individuals are highly likely to be diagnosed and channeled into isolation (Iso) and/or treatment (Trt) pathways, whereas unaware individuals have a lower probability of diagnosis and typically recover without intervention. Viral Introductions (orange arrow) represent phylodynamics-informed imported cases, which enter the model directly into the symptomatic, aware state. These cases contribute to ongoing transmission but are not counted as locally acquired diagnoses in LAC. All individuals eventually exit via recovery or non–mpox-related death (red dashed arrows). Adapted from Liang et al. (18)

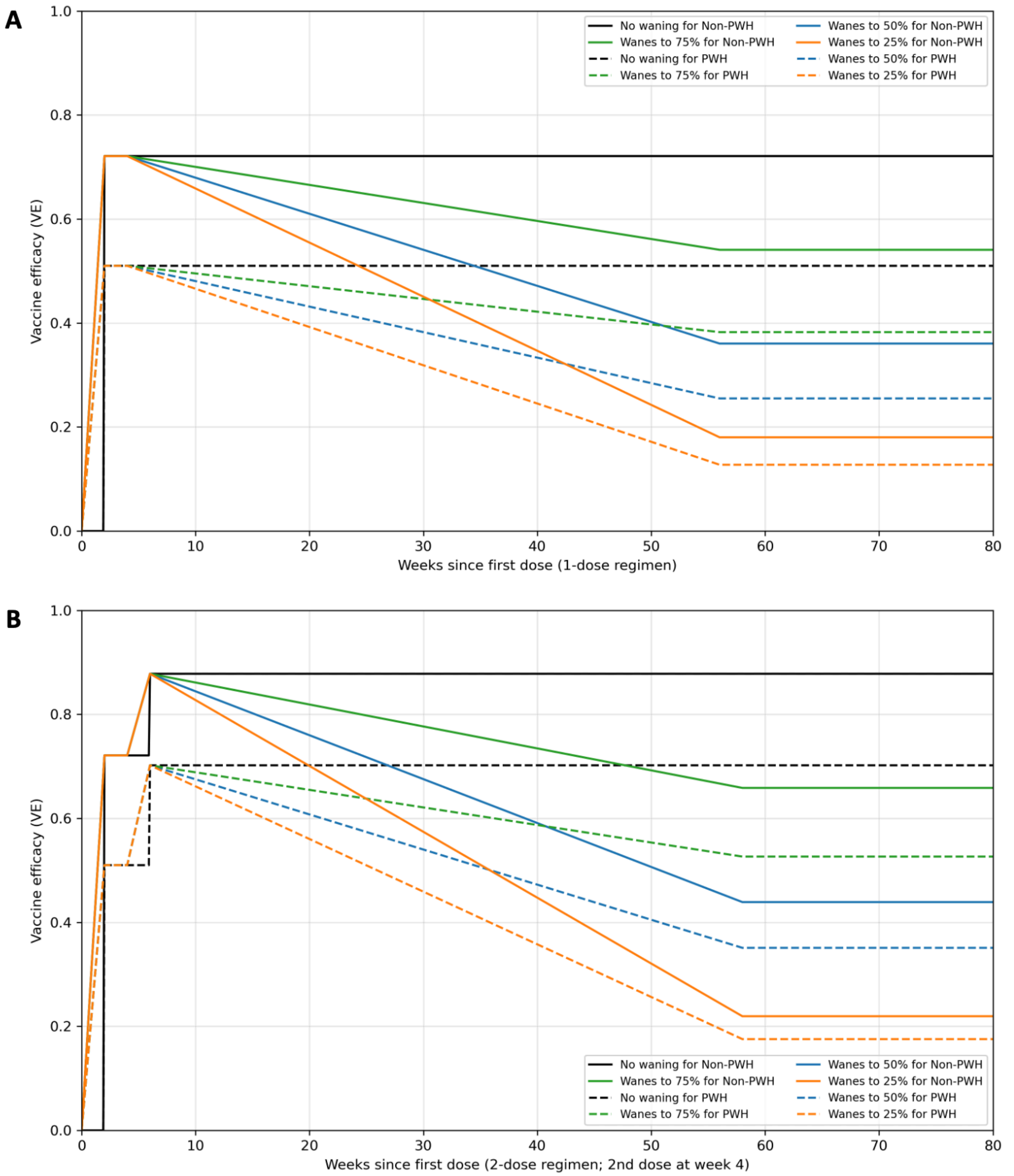

Figure S22. Vaccine efficacy over time by waning scenario and HIV status. (A) One-dose regimen. (B) Two-dose regimen. For the one-dose regimen (Panel A), vaccine efficacy increases linearly from zero to peak over 2 weeks following the first dose, remains at peak for 2 weeks, then wanes linearly over 52 weeks to a target fraction of peak efficacy. For the two-dose regimen (Panel B), vaccine efficacy follows the one-dose trajectory through week 4, then increases linearly over 2 weeks (weeks 4–6) following the second dose to reach full two-dose efficacy, and subsequently wanes linearly over 52 weeks (weeks 6–58) to a target fraction of peak efficacy. Solid lines represent HIV-negative individuals; dashed lines represent people with HIV (PWH). The base case assumes waning to 50% of peak efficacy (blue); sensitivity analyses explore waning to 25% (orange) and 75% (green) of peak efficacy. The no-waning scenario (black) maintains constant efficacy after the initial development period as a reference comparison.

Parameter	Value	Source
**Initial Population (March 2023)**		
LAC total MSM population	263,322	*
Mpox symptomatic cases (Undiagnosed)	2	*
Mpox symptomatic cases (Diagnosed)	3	*
Individuals Recovered from Mpox	1,337	*
Mpox vaccinated (1st dose)	19,563	*
Mpox vaccinated (Fully vaccinated)	45,520	*
**Weekly Transition Probabilities**		
**Demographics**		
Weekly entry rate (fraction of current MSM)	0.0003	†
**Disease Progression (Natural History)**		
Mpox asymptomatic Symptomatic	0.50	†
Mpox asymptomatic Recovered (post-vaccination)	0.43	†
Mpox symptomatic Recovered (untreated)	0.28	†
Mpox symptomatic Recovered (treated)	0.50	†
**Diagnosis P(Mpox undiagnosed Diagnosed)**		
Asymptomatic (HIV-negative or Status Unknown)	0.01	†
Asymptomatic (HIV-positive, Aware)	0.10	†
Symptomatic (by age group) ‡	0.7 - 0.9	†
**Vaccination Uptake P(Unvaccinated Vaccinated)**		
1st dose (Susceptible or Unaware asymptomatic)	0.0018	§
1st dose (Aware asymptomatic)	0.95	†
2nd dose (Eligible) ‖	0.50	†
**Vaccine efficacy**		
1st dose and HIV-positive	0.51	†
1st dose and HIV-negative	0.72	†
2nd dose and HIV-positive	0.70	†
2nd dose and HIV-negative	0.88	†

* Derived from simulation outputs in Liang et al. (18)

† Parameters adopted from Liang et al. (18)

‡ Diagnosis probabilities by age group: 15–29 (0.8), 30–39 (0.9), 40–49 (0.8), 50–59 (0.9), 60–100 (0.7).

§ Calibrated to match observed vaccination trends in LAC.

‖ Eligible definition: individuals who received the 1st dose vaccination 4-6 weeks prior and remain susceptible or undiagnosed asymptomatic

Table S1: Key Model Inputs and parameters for the microsimulation. The table summarizes the initial population conditions for March 2023, weekly transition probabilities governing demographic turnover, disease progression, diagnosis, and vaccination uptake. Parameters marked with * are derived from simulation outputs in Liang et al (18); those marked with † are adopted directly from Liang et al (18); and those marked with § are calibrated to updated surveillance data.
