# Supplementary material for: Viral introductions and return to baseline sexual behaviors maintain low-level mpox incidence in Los Angeles
Source: Nat Commun. 2026 Apr 17;17:5376. doi: 10.1038/s41467-026-71993-w (PMC13276399; doi:10.1038/s41467-026-71993-w)
Supplement: Supplementary file 3 — Reporting Summary [file 41467_2026_71993_MOESM3_ESM.pdf]

nature portfolio

Corresponding author(s): Miguel I. Paredes, Citina Liang

Last updated by author(s): 03/08/2026

Reporting Summary

Nature Portfolio wishes to improve the reproducibility of the work that we publish. This form provides structure for consistency and transparency in reporting. For further information on Nature Portfolio policies, see our Editorial Policies and the Editorial Policy Checklist.

Statistics

For all statistical analyses, confirm that the following items are present in the figure legend, table legend, main text, or Methods section.

n/a	Confirmed	
☐	☒	The exact sample size (*n*) for each experimental group/condition, given as a discrete number and unit of measurement
☐	☒	A statement on whether measurements were taken from distinct samples or whether the same sample was measured repeatedly
☐	☒	The statistical test(s) used AND whether they are one- or two-sided<br>*Only common tests should be described solely by name; describe more complex techniques in the Methods section.*
☒	☐	A description of all covariates tested
☐	☒	A description of any assumptions or corrections, such as tests of normality and adjustment for multiple comparisons
☐	☒	A full description of the statistical parameters including central tendency (e.g. means) or other basic estimates (e.g. regression coefficient) AND variation (e.g. standard deviation) or associated estimates of uncertainty (e.g. confidence intervals)
☐	☒	For null hypothesis testing, the test statistic (e.g. *F*, *t*, *r*) with confidence intervals, effect sizes, degrees of freedom and *P* value noted<br>*Give P values as exact values whenever suitable.*
☐	☒	For Bayesian analysis, information on the choice of priors and Markov chain Monte Carlo settings
☒	☐	For hierarchical and complex designs, identification of the appropriate level for tests and full reporting of outcomes
☐	☒	Estimates of effect sizes (e.g. Cohen's *d*, Pearson's *r*), indicating how they were calculated

Our web collection on statistics for biologists contains articles on many of the points above.

Software and code

Policy information about availability of computer code

Data collection	Data on the number of diagnosed mpox cases in Los Angeles County were downloaded from the Los Angeles County mpox data dashboard (http://publichealth.lacounty.gov/media/monkeypox/data/index.htm/ ; last accessed on 01-20- 2025).<br>All available MPXV sequences were downloaded from GenBank on 01-20-2024. All sequences are available on GenBank with accession numbers found in https://github.com/blab/mpox-la/tree/main.
Data analysis	Nextstrain builds, BEAST2 XMLs, scripts, sequence information, and de-identified data for the phylogenetic and phylodynamic analyses can be found at https://github.com/blab/mpox-la/tree/main. All sequences are available on GenBank with accession numbers found in the supplementary information. The code for the microsimulation model developed to study mpox incidence and dynamics is available at https://github.com/citina/microsimulation-mpox, which includes all scripts, parameter files, and usage instructions necessary to replicate the study findings.

For manuscripts utilizing custom algorithms or software that are central to the research but not yet described in published literature, software must be made available to editors and reviewers. We strongly encourage code deposition in a community repository (e.g. GitHub). See the Nature Portfolio guidelines for submitting code & software for further information.

Data

Policy information about availability of data

All manuscripts must include a data availability statement. This statement should provide the following information, where applicable:

- Accession codes, unique identifiers, or web links for publicly available datasets
- A description of any restrictions on data availability
- For clinical datasets or third party data, please ensure that the statement adheres to our policy

Nextstrain builds, BEAST2 XMLs, scripts, sequence information, and de-identified data for the phylogenetic and phylodynamic analyses can be found at https://github.com/blab/mpox-la/tree/main. All sequences are available on GenBank with accession numbers found in the supplementary information and in the above github repo. The code for the microsimulation model developed to study mpox incidence and dynamics is available at https://github.com/citina/microsimulation-mpox, which includes all scripts, parameter files, and usage instructions necessary to replicate the study findings.

Research involving human participants, their data, or biological material

Policy information about studies with human participants or human data. See also policy information about sex, gender (identity/presentation), and sexual orientation and race, ethnicity and racism.

Reporting on sex and gender	N/A
Reporting on race, ethnicity, or other socially relevant groupings	N/A
Population characteristics	N/A
Recruitment	N/A
Ethics oversight	All data used in this study is publicly available, suitably anonymized viral sequence or epidemiological data. As such it does not constitute human-subjects research.

Note that full information on the approval of the study protocol must also be provided in the manuscript.

Field-specific reporting

Please select the one below that is the best fit for your research. If you are not sure, read the appropriate sections before making your selection.

☐ Life sciences ☐ Behavioural & social sciences ☒ Ecological, evolutionary & environmental sciences

For a reference copy of the document with all sections, see nature.com/documents/nr-reporting-summary-flat.pdf

Ecological, evolutionary & environmental sciences study design

All studies must disclose on these points even when the disclosure is negative.

Study description	This observational study integrates phylogenetic, phylodynamic, and microsimulation modeling techniques to understand the factors promoting low level mpox spread in Los Angeles County. Using whole-genome sequenced data, Bayesian phylodynamics inference methods and modified stochastic microsimulation modeling that is informed by the phylodynamics, the study aims to determine the interplay of introductions vs local transmission of mpox as well as how behavioral changes might affect case counts.
Research sample	The research sample consists of 7,859 whole genome sequences of MPXV along with related metadata, that were available on the GenBank database on 20 January 2025
Sampling strategy	All available MPXV sequences were downloaded from GenBank on 01-20-2024. Sequences with ambiguous date of collection in the month column, with a sample collection earlier than January 2022, and flagged as being low quality by Nextclade were excluded. Given that mpox transmission in the United States is driven by clade IIb viruses, sequences from other clades were also excluded, resulting in 7859 genome sequences included in our analysis.
Data collection	We gratefully acknowledge all data contributors, i.e., the authors and their originating laboratories responsible for obtaining the specimens, and their submitting laboratories for generating the genetic sequence and metadata and sharing via GenBank. We have included a detailed acknowledgment table in https://github.com/blab/mpox-la .
Timing and spatial scale	We included all clade IIb MPXV viruses found in Genbank from January 1 2022 to December 12, 2024. We included all sequences from around the globe. To conduct our phylogeographic and subsequent LA county focused analyses and due to the low number of sequences from various countries, we analyzed mpox spread with a focus on large metropolitan US cities and areas that have the highest level of mpox sequencing effort. Our focus areas were: Los Angeles County, California; Washington State; Cook County, Illinois; New York City, New York; California without Los Angeles County; North America excluding the areas previously mentioned; and Global regions outside of North America.

Given that Los Angeles County Department of Public Health (LA DPH) sequences the mpox cases for LAC, we assume that any genome labelled as being sequenced by LA DPH was sampled in LAC, while those sampled by the California Department of Health (CDPH) were sampled in locations within California but outside of LAC. From these 719 genomes, the dataset was filtered down to 497 by LA DPH to remove duplicated sequences from the same individual and samples that were collected outside of LA DPH. Despite this, there is always a small chance that CDPH might have received and sequenced a LAC case, but we expect this to be small and should result in a conservative bias as misclassification of an LAC sequence as non-LAC would result in smaller clusters and less intense transmission dynamics.

Data exclusions	Sequences with ambiguous date of collection in the month column, with a sample collection earlier than January 2022, and flagged as being low quality by Nextclade were excluded. Given that mpox transmission in the United States is driven by clade IIb viruses, sequences from other clades were also excluded, resulting in 7859 genome sequences included in our analysis. For our LA county specific genomes, the dataset of 719 LA county genomes was filtered down to 497 by LA DPH to remove duplicated sequences from the same individual and samples that were collected outside of LA DPH.
Reproducibility	To ensure reproducibility of the findings, the study utilized widely accessible open-source tools and methodologies. The methods outlining the data acquisition and analysis are described in detail. All relevant scripts have been uploaded to our GitHub repository (https://github.com/blab/mpox-la for phylodynamic analyses and https://github.com/citina/microsimulation-mpox for microsimulation analyses), and links to the sequences used in the study are included in https://github.com/blab/mpox-la . We also ran twenty independent iterations of each microsimulation scenario to capture inherent variability in the outcomes
Randomization	Randomization is not relevant to this study
Blinding	Blinding is not relevant to this study

Did the study involve field work? ☐ Yes ☒ No

Reporting for specific materials, systems and methods

We require information from authors about some types of materials, experimental systems and methods used in many studies. Here, indicate whether each material, system or method listed is relevant to your study. If you are not sure if a list item applies to your research, read the appropriate section before selecting a response.

Materials & experimental systems

n/a	Involved in the study
☒	☐ Antibodies
☒	☐ Eukaryotic cell lines
☒	☐ Palaeontology and archaeology
☒	☐ Animals and other organisms
☒	☐ Clinical data
☒	☐ Dual use research of concern
☒	☐ Plants

Methods

n/a	Involved in the study
☒	☐ ChIP-seq
☒	☐ Flow cytometry
☒	☐ MRI-based neuroimaging

Plants

Seed stocks	*Report on the source of all seed stocks or other plant material used. If applicable, state the seed stock centre and catalogue number. If plant specimens were collected from the field, describe the collection location, date and sampling procedures.*
Novel plant genotypes	*Describe the methods by which all novel plant genotypes were produced. This includes those generated by transgenic approaches, gene editing, chemical/radiation-based mutagenesis and hybridization. For transgenic lines, describe the transformation method, the number of independent lines analyzed and the generation upon which experiments were performed. For gene-edited lines, describe the editor used, the endogenous sequence targeted for editing, the targeting guide RNA sequence (if applicable) and how the editor was applied.*
Authentication	*Describe any authentication procedures for each seed stock used or novel genotype generated. Describe any experiments used to assess the effect of a mutation and, where applicable, how potential secondary effects (e.g. second site T-DNA insertions, mosiacism, off-target gene editing) were examined.*
