# Supplementary material for: Viral introductions and return to baseline sexual behaviors maintain low-level mpox incidence in Los Angeles
Source: Nat Commun. 2026 Apr 17;17:5376. doi: 10.1038/s41467-026-71993-w (PMC13276399; doi:10.1038/s41467-026-71993-w)
Supplement: Supplementary file 2 — Peer Review file [file 41467_2026_71993_MOESM2_ESM.pdf]

nature portfolio

Peer Review File

Viral introductions and return to baseline sexual behaviors maintain low-level mpox incidence in Los Angeles

Corresponding Author: Dr Miguel Paredes

This file contains all reviewer reports in order by version, followed by all author rebuttals in order by version.

Version 0:

Reviewer comments:

Reviewer #1

(Remarks to the Author)
Paredes et al. present an important study highlighting the dynamics of MPXV in Los Angeles County (LAC) after the 2022 global epidemic. Using a combination of phylodynamics and microsimulations, the authors showcase the role of importations in sustaining low-level transmission of MPXV and that local transmission alone would have likely been unable to cause the observed case numbers in LAC. Their results suggest that a return to near-baseline sexual behavior in 2023¬–2024, or at least a relative increase compared to behavior during the peak of the epidemic in 2022, explains observed case counts. This is a valuable contribution bridging genomic epidemiology with public health modelling, and I only have a few suggestions, delineated below.

1. Consider presenting the microsimulation model after the phylodynamic analysis. Currently, the text introduces a version of the model that initially does not incorporate time-varying importations, only to later re-parameterize and improve upon it. The text could be streamlined by starting with the phylodynamics and importation dynamics, followed by transitioning to the coupled phylogeography and microsimulations.
2. The results described in lines 246–249 and the large peaks of Ne in late 2023 and early-mid 2024 in Fig. 3B suggest underdetected transmission. Was there a concomitant increase in viral diversity during those time periods supporting this hypothesis?
3. The dot sizing and coloring in Fig. S5A make it difficult to interpret cluster sizes. Consider simplifying this visual (e.g., using just size or color), and if size is retained, please include a legend with example dot sizes. The legend for Fig. S5 describes error bars – are those for S5B? Is the CI very tight? I was unable to identify any error bars.
4. Lines 340–341: the authors write that removing the influence of viral importations results in Rt getting “close” to 1; perhaps “closer” to 1 would be more appropriate, as the estimates are quite close to 1 already for all methods aside from the microsimulations.
5. Fig. 4C: are the scatter points also sized based on the size of the resulting transmission cluster?
6. There appears to be sizeable uncertainty in most of Fig. 4D, and it’s unclear whether the uncertainty increases specifically when case counts are low. Is there a metric (or simple plot) the authors could create or use to correlate case counts with uncertainty? Also, please clarify what the colors in Fig. 4D correspond to.
7. In the paragraph starting at line 433, the text refers to a sensitivity analysis on vaccine waning but does not specify the baseline assumption used in the main simulations. Please state what the effectiveness is after 1 year in the primary simulations (50% of the initial effectiveness?).
8. In the microsimulations, do all importations result in infected individuals at the onset of symptomatic infection? If so, could this lead to an underestimation of importations (by missing importations with partially elapsed symptomatic phases)? It may be worthwhile to examine the consequences of importations arriving at different points within the symptomatic infection (e.g., some arriving at the start of the infection, others halfway through, etc.).
9. Since LAC often sequenced >50% of cases, it could be useful to repeat the analysis represented in Fig. S8A under multiple assumed sequencing coverages and contextualize the results accordingly. Also, please state what the black cross represents in the legend.

Minor comments:
1. Fig. S3: please consider using matching y-axis limits for the two bottom panels. Could have insets that zoom in on low values.
2. Fig. S10: did the authors mean blue, rather than yellow?

3. Fig. S16: please consider including what the different arrows and abbreviations represent in the legend, as in the original figure in Liang et al.

(Remarks on code availability)
I have briefly reviewed the code; it appears thorough and reproducible. The code does appear to provide enough instructions to replicate much of the results, but I have not attempted to reproduce the analysis myself.

Reviewer #2

(Remarks to the Author)
Summary
The manuscript “Viral introductions and return to baseline sexual behaviors maintain low-level mpox incidence in Los Angeles County, USA, 2023-2024” presents results from two years of the mpox epidemic in Los Angeles County derived from both phylodynamic and microsimulation analysis. The authors find strong evidence that the mpox epidemic is currently driven primarily by importations rather than local transmission, and I appreciated the integration of epidemiological and phylodynamic lines of evidence. My main concerns are with the calibration of the microsimulation model and the simulated intervention scenarios, but I believe these concerns can be addressed in a revised version of the manuscript. Overall, the work presents a novel view of recent mpox transmission relevant to many cities in the United States, and I applaud the authors on their comprehensive undertaking!

Major Comments

My primary concern is with the calibration of the microsimulation model to the case data. The alpha parameter controls a large portion of the transmission dynamics and thus far the authors have calibrated it in an ad-hoc manner (e.g. letting it vary during 3 time points of the epidemic), so conclusions drawn from the microsimulations need to be qualified. I have two key concerns:

The authors note: “It showed, however, that without viral introductions into the county, mpox incidence would have been expected to drop to zero by March 2023,” in reference to the orange line in Figure 1A, but the authors had to alter alpha to match both the increase and decline in the epidemic. One could likely match the subsequent dynamics (early 2023 onward) through a model where alpha was increased to put the reproduction number fluctuating near 1 rather than adding in the importations). This also relates to the conclusions from Figure 1D (e.g. “With the same model parameters calibrated after August 2022, the model required a large number of mpox introductions (> 5 per week) to maintain ongoing transmission similar to the empirical number of cases (Fig. 1B).” That sentence is correct, but the authors suggest these results as evidence for importations being important to the subsequent dynamics, even though they do not compare it to model simulations where alpha during that time period is roughly calibrated to the case counts. If the authors would like to make the claim from these data, they should compare it against a counterfactual where the transmission pressure (alpha) was the primary driver of the dynamics and assess model fits between the two. Alternatively, the phylodynamic evidence is quite strong for the importance of importations for sustaining transmission, so I suggest the authors focus more on that for those conclusions rather than the microsimulation work presented in figure 1.

For Figure 5, the calibration becomes much more important. The authors “calibrate” alpha to the case counts, but ultimately their best fit value of 2.0 does not match the case time-series well (though I admit it does get roughly the cumulative case count correct). It is clear that alpha must fluctuate over time even during this time period to recreate the results, for example, potentially alpha needs to be increased in the Spring of 2023 to recreate the summer bump in cases, and then potentially decreased again. As it stands, the importation surge in the summer of 2023 is leading to increased case counts in fall/winter of 2023 rather than in the summer of 2023 leading to a case/simulation mismatch. Based on the data available to them, I would suggest that the authors implement a more systematic calibration of alpha to more closely align the case counts from the microsimulation to the data itself. If this is computationally infeasible, then the authors need to very strongly justify their manual calibration procedure and I would suggest allowing alpha to vary more widely over time to get a closer match with the data. This is particularly important for Figure 5, because the interventions that impact alpha are going to have very different impacts on case counts depending on how the timing of those interventions align with the fluctuations of alpha and the imported case seasonality.

I like the principle behind the idea for Figure 5C and D, but based on the authors results I am having trouble understanding the conclusions. Looking at the interventions it appears that randomly timing interventions is always better or equal to timing the intervention during the period of high importation pressure. Based on Fig S13, I think this is because the random interventions occur throughout the whole time period whereas the targeted interventions occur only in 2023. If this is the case, the authors should instead run the simulations where the random weeks are chosen randomly only for 2023 as a comparison rather than being spaced over the whole time period. If it’s not because of that, then the authors should think deeply about why the random interventions are having a disproportionately large impact as that may provide an interesting public health strategy to reduce future case counts.

Related to my above comment, I am a bit puzzled at the choice of interventions the authors explore in Figure 5. First, I was curious why the authors did not explore what strategies are necessary to push mpox case counts down to zero as much as

possible. If that truly is the public health goal it would be helpful to know what needs to happen (e.g. how low does alpha need to be to get there?). If that isn't the goal, then I would suggest the authors more clearly state what the public health objectives would be in these scenarios (e.g. why they only consider scenarios where alpha is lowered to the extent they lower it in the simulations). Second, and potentially more importantly, in the previous results before this figure the authors showed decisively that the reproduction number is well below one and that case counts are driven primarily from importation-associated transmission. If that's the case, it seems non-optimal to focus public health interventions on reducing local transmission, and it would be useful for the authors to compare with a different control strategy that might be more impactful (e.g. reducing importations). If you can reduce importations by 50% how does that compare in cumulative case counts to the strategies where you are simply reducing transmission? How much would you need to reduce importations to get the cumulative case counts near zero? There are a number of ways one could imagine public health officials focusing their efforts on reducing importations that would be different than how they might reduce transmission (alpha), and it would likely be helpful to run this analysis to help them allocate their resources appropriately (my hypothesis would be that one could get a better bang for the buck focusing on importations, but I'm curious to see what the modeling results suggest).

It would be great if the authors could include more of a discussion on the fluctuation of viral importations they identified. Is this linked to epidemic waves in other regions that the authors discuss (e.g. NYC, Chicago, California, etc)? Is it linked to specific events (e.g. pride)? Something related to the seasonality of travel? The authors note in the discussion "Of note, both sexual behavior and travel vary by season" and mention how summer travel is particularly large, but this wouldn't seem to explain the bump in importations in Feb-April of 2024 or the pattern of monthly importations from Figure 4B.

The authors reference Ref 18 for almost all of the details about the microsimulation, but it would be helpful if some of the key components of the model were included in this paper or the associated supplemental information rather than having to flip back and forth between two papers. For example, one could include the overall model architecture or diagram (e.g. the process and measurement models), the table of parameters associated with that paper, initialization values, simulation timelines, etc.

Minor Comments

Line 170: remove errant "the."
Line 182: should reference Fig. 1D.
Figure 5A: color and interval distinctions are difficult to differentiate; consider alternative schemes.
Figure 5C: the figure may be clearer if the grey case bars are removed, since the main purpose is to compare scenarios rather than match to data.

(Remarks on code availability)
I did not attempt to recreate the analyses, but upon my quick glance through the code it appears to be reasonably well organized and documented for reproducibility purposes.

Reviewer #3

(Remarks to the Author)
Parades et al., performed a phylodynamic analysis and simulation on mpox incidence in Los Angeles County, 2023-2024. The study is well performed, the manuscript is well written and the figures are excellent.
I have some comments to be addressed:
- Line 133, there is an extra point.
- Please report in the figure 1 legend the study period(from.. to ..).
-In the Model overview, there is no mention of importations. Do the authors think it could have affected the model?
- I suggest reporting the resulting phylogeny in the results (lines 947-948).
- Considering the inclusion criteria of the genomes and the filter, I have concerns about the limitations of the local transmission. Since the inclusion criteria were very strict, I am afraid that a part of the introduction history is missing.
- Were the genomes aligned?
- Line 211. "...we analyzed 497 mpox genomes..." and Figure 2 legend " we analyzed more than 7,500 publicly available mpox clade IIb genomes...". I am confused about these more than 7,500 publicly available mpox clade IIb genomes. Please clarify and report the actual number.

(Remarks on code availability)

Version 1:

Reviewer comments:

Reviewer #1

(Remarks to the Author)
The authors have done an excellent job responding to my and the other reviewer's feedback, and I only have minor suggestions prior to publication.

For Fig. S7, would it be possible to make the circles a little smaller? It's challenging to see everything in the plot given their current size.

Based on Figures S15–16, it could be worthwhile to comment on how simulating individuals at different stages of infection and symptom/diagnosis states affects the magnitude of return to baseline sexual activity required to recapitulate empirical trends (and, equally importantly, the conditions under which sustained, albeit limited, transmission is maintained). At a practical level, this is an important public health finding.

(Remarks on code availability)

Reviewer #2

(Remarks to the Author)
The authors have thoroughly addressed all of the concerns raised in the previous review. The revisions have substantially strengthened the clarity, rigor, and overall contribution of the manuscript. I commend the authors on an excellent paper and congratulate them on this high-quality work.

(Remarks on code availability)
Nothing different than my previous comments.

Reviewer #3

(Remarks to the Author)
The authors have done an exceptional job in addressing the reviewers' comments. The manuscript has truly benefited from these clarifications, rephrasing, and additions. It is now ready for publication and will undoubtedly be a valuable addition to the journal's publication.

(Remarks on code availability)

Open Access This Peer Review File is licensed under a Creative Commons Attribution 4.0 International License, which permits use, sharing, adaptation, distribution and reproduction in any medium or format, as long as you give appropriate credit to the original author(s) and the source, provide a link to the Creative Commons license, and indicate if changes were made.
In cases where reviewers are anonymous, credit should be given to 'Anonymous Referee' and the source.
The images or other third party material in this Peer Review File are included in the article's Creative Commons license,

unless indicated otherwise in a credit line to the material. If material is not included in the article's Creative Commons license and your intended use is not permitted by statutory regulation or exceeds the permitted use, you will need to obtain permission directly from the copyright holder.
To view a copy of this license, visit https://creativecommons.org/licenses/by/4.0/

Reviewer’s Comments:

Reviewer #1 (Remarks to the Author)

Paredes et al. present an important study highlighting the dynamics of MPXV in Los Angeles County (LAC) after the 2022 global epidemic. Using a combination of phylodynamics and microsimulations, the authors showcase the role of importations in sustaining low-level transmission of MPXV and that local transmission alone would have likely been unable to cause the observed case numbers in LAC. Their results suggest that a return to near-baseline sexual behavior in 2023¬–2024, or at least a relative increase compared to behavior during the peak of the epidemic in 2022, explains observed case counts. This is a valuable contribution bridging

genomic epidemiology with public health modelling, and I only have a few suggestions, delineated below.

Thank you so much for the comments and your kind words!

1. Consider presenting the microsimulation model after the phylodynamic analysis. Currently, the text introduces a version of the model that initially does not incorporate time-varying importations, only to later re-parameterize and improve upon it. The text could be streamlined by starting with the phylodynamics and importation dynamics, followed by transitioning to the coupled phylogeography and microsimulations.

Thank you for the suggestion! This was also suggested by Reviewer 2 and thus we have changed the narrative structure of the manuscript to start with general mpox epidemiology in LAC, followed by phylodynamic analysis of importations, and finishing with coupled phylo and microsimulation.

2. The results described in lines 246–249 and the large peaks of Ne in late 2023 and early-mid 2024 in Fig. 3B suggest underdetected transmission. Was there a concomitant increase in viral diversity during those time periods supporting this hypothesis?

Locally, the diversity of mpox will largely be driven by introductions from elsewhere. Or in other words, diversity in mpox is created everywhere where there is transmission. In our case, the Ne tau is a measure of how rapidly we observe coalescence locally. As such Ne tau is reflective only of the transmission patterns locally and not the standing diversity in the global viral population at that time. Volz (2009 and 2012) shows how in phylodynamic model, Ne can be expressed using the parameters in an SIR model such that $N_e\tau = \frac{I(t)}{2\beta S(t)}$, where I(t) denotes the number of infected individuals, S(t) the number of susceptibles, β is the transmission rate, and τ denotes the generation time, so that Ne is not directly affected by changes in genetic diversity but rather changes in incidence and prevalence of the disease.

Another way to think about it is that the diversity informs the tree, with branch lengths in units of time, not genetic distance. Ne tau then reflects how rapidly we observe lineages to coalesce, but does not directly correspond to a measure of genetic diversity.

3. The dot sizing and coloring in Fig. S5A make it difficult to interpret cluster sizes. Consider simplifying this visual (e.g., using just size or color), and if size is retained, please include a legend with example dot sizes. The legend for Fig. S5 describes error bars – are those for S5B? Is the CI very tight? I was unable to identify any error bars.

We’ve simplified Fig. S5A (now Fig S7) and just used color to represent cluster sizes for easier visualization as seen below. Additionally, we thank the reviewer for catching the lack of intervals, there was an issue with the script so we’ve replotted everything with matplotlib and highlighted

the HPD intervals. The figure legend was also changed to highlight the fact that the intervals are HPDs and not CI as follows: “The error bars represent the 95% HPD and these estimates were used to parameterize our microsimulation model. “

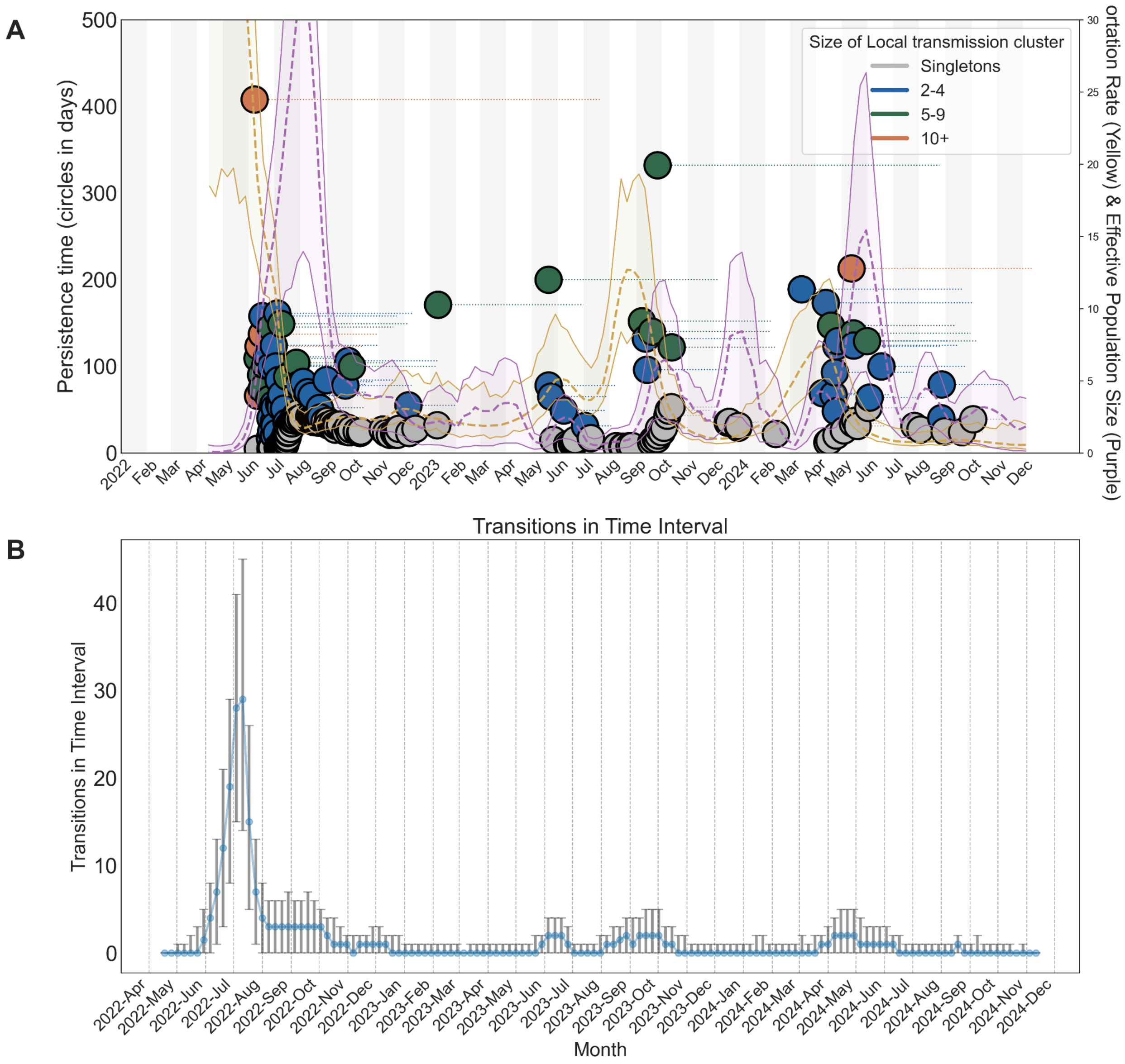

Figure S7: Mpox importation dynamics in LA County estimated via Bayesian Phylodynamics. Panel **A** shows the persistence time of each identified local outbreak cluster according to the date of its inferred introduction time. Each dot represents an inferred introduction into LA County, the radius of the dot is proportional to the size of the resulting transmission cluster. The yellow streamgraph is the rate of introduction (events/lineage/year) into LA county and the purple streamgraph represents the estimated effective population size**,** both inferred by our phylodynamic model. The dashed line represents the median and the bands represent the 95% HPD. Panel **B** shows the absolute number of viral introductions inferred via our phylodynamic model for each week that was calculated by analyzing the entire posterior set of phylogenetic trees after burn in. The error bars represent the 95% HPD and these estimates were used to parameterize our microsimulation model.

4. Lines 340–341: the authors write that removing the influence of viral importations results in Rt getting “close” to 1; perhaps “closer” to 1 would be more appropriate, as the estimates are quite close to 1 already for all methods aside from the microsimulations.

Thank you for catching that! We’ve changed it to “closer”:
“Removing the influence of viral importations dropped the median Rt estimate closer to 1 with high variability.”

5. Fig. 4C: are the scatter points also sized based on the size of the resulting transmission cluster?

They are, but similar to Fig S5A, we’ve removed the double encoding and just left color to represent cluster sizes as seen below:

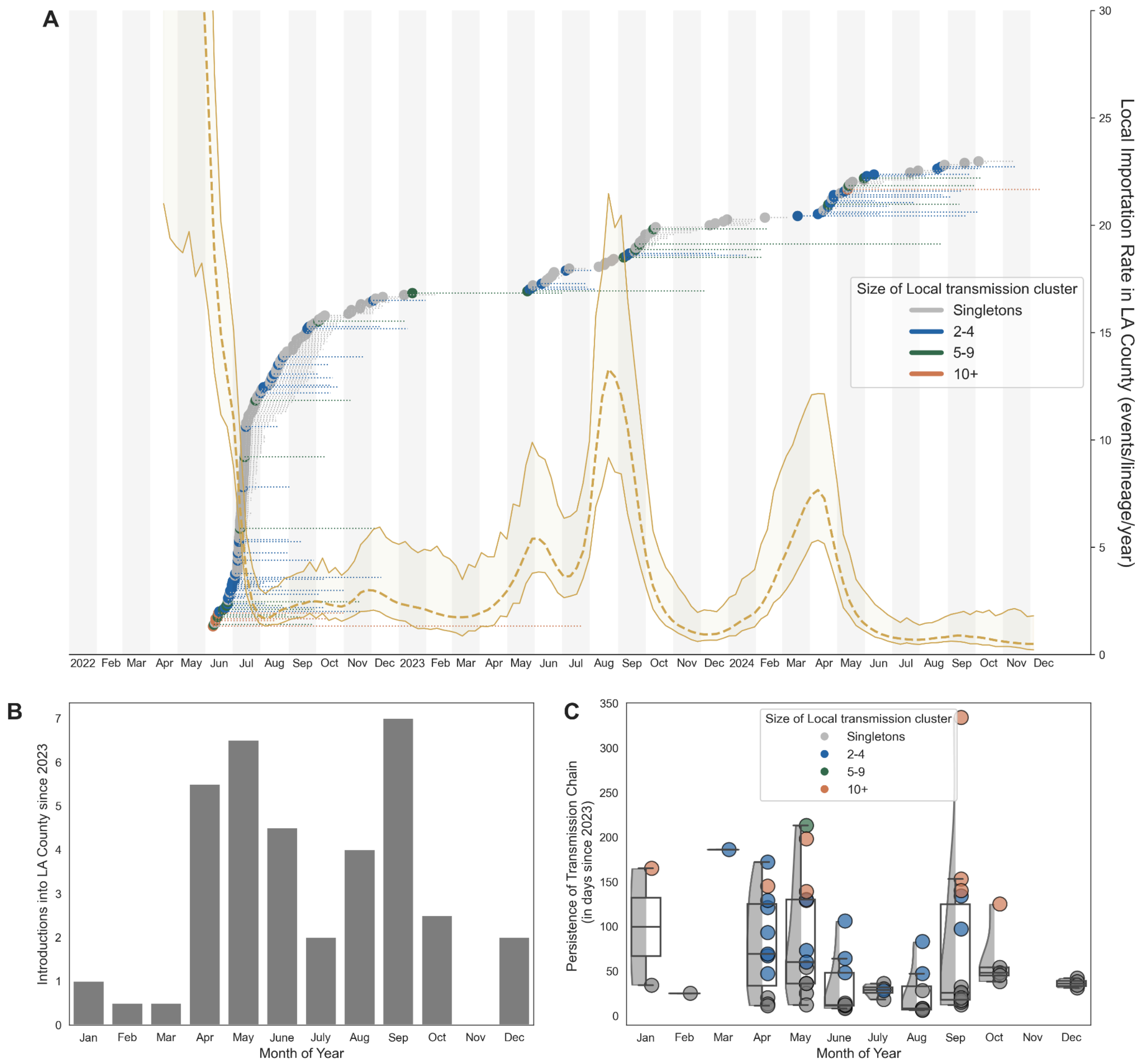

6. There appears to be sizeable uncertainty in most of Fig. 4D, and it's unclear whether the uncertainty increases specifically when case counts are low. Is there a metric (or simple plot) the authors could create or use to correlate case counts with uncertainty? Also, please clarify what the colors in Fig. 4D correspond to.

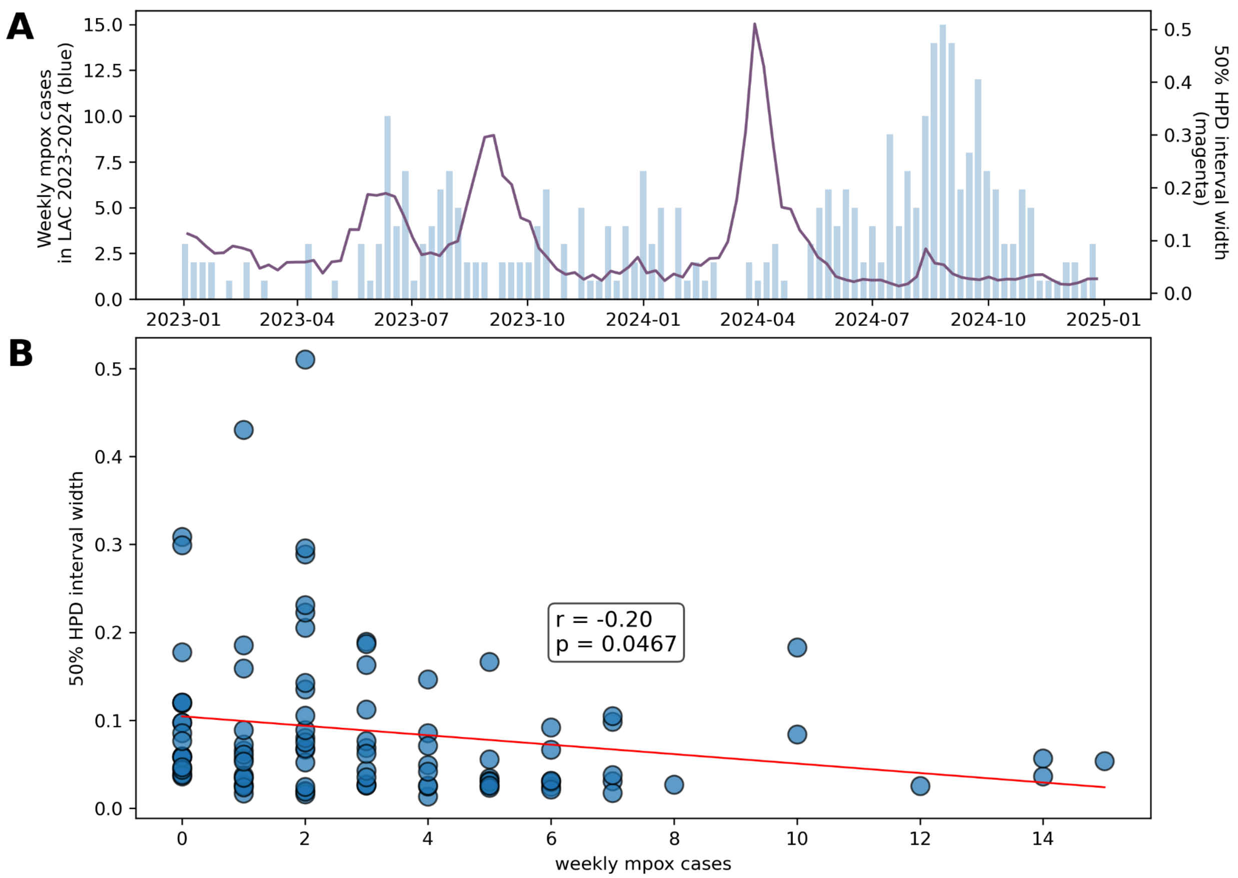

Figure S8: Relationship between uncertainty in percentage of cases due to introductions and case counts. Panel **A** plots the weekly case counts in LAC from March 2023 through December 2024 in light blue. The purple line denotes the width of the 50% HPD interval found for the estimated percentage of cases due to introductions found in Fig 3D as a measure of uncertainty. Panel **B** shows the relationship between the weekly number of mpox cases in LAC and the width of the same 50% HPD interval. The red line is a linear regression of the scatterplot with the associated Pearson correlation coefficient and p value.

In Panel A above, we use the width of the 50% HPD interval (defined as the difference between the upper and lower bound, plotted in magenta) to represent the degree of time varying uncertainty, where a larger interval denotes greater uncertainty. When plotted against the weekly number of mpox cases in LAC from 2023-2024, we see, in agreement to the reviewer's comment, that the highest periods of uncertainty are often when the weekly number of cases are lowest. We further explore this correlation in Panel B by creating a scatterplot of weekly mpox cases since 2023 on the x-axis and HPD interval width on the y-axis. Using linear regression, we see additional support of a negative correlation whereby increased uncertainty is associated with lower weekly mpox cases. We have included this figure in the manuscript as Figure S8 and have referenced it as " In periods of low case counts, we see a wide uncertainty (Fig. 3D, S8, light bands), highlighting the potentially increased impact of introductions during those time periods."

Additionally, the figure legend for Fig. 4D was updated as follows: "The percentage of new cases due to introductions was estimated as the relative contribution of introductions to the overall

number of infections in the region, with the dark magenta band representing the 50% HPD and the lighter magenta representing the 95% HPD. “

7. In the paragraph starting at line 433, the text refers to a sensitivity analysis on vaccine waning but does not specify the baseline assumption used in the main simulations. Please state what the effectiveness is after 1 year in the primary simulations (50% of the initial effectiveness?).

Thank you for the suggestions; we modified the sentence as follows: “We also tested the sensitivity of our findings by increasing and decreasing the average effectiveness of mpox vaccination against infection after one year by 25% *(from a baseline one-year average effectiveness drops to 50% of its peak value)* and find that the cumulative number of empirical mpox cases still falls within the 95% CI of α of 2.0 (Fig S14).”

To address the reviewer’s broader concern regarding vaccine waning, we also added a more explicit description of the vaccine efficacy trajectory in the manuscript. This text has been included in the *Microsimulation Model* section, *Model Overview* subsection:

“We also replaced the previous assumption of constant vaccine efficacy with a time-varying, piecewise linear vaccine efficacy (VE) function. VE is modeled separately for people with HIV (PWH) and HIV-negative individuals and for one-dose versus two-dose regimens. Following vaccination, VE increases from zero to its peak over two weeks, remains at peak for two weeks, and then wanes linearly over 52 weeks. In the base case, VE declines to 50% of its peak one year after vaccination (e.g., two-dose VE in HIV-negative individuals declines from 87.8% to 43.9%) (47, 48, 60, 61). Sensitivity analyses evaluate alternative waning assumptions of 25% and 75% of peak efficacy (see Fig. S22 for the vaccine waning efficacy visualization)."

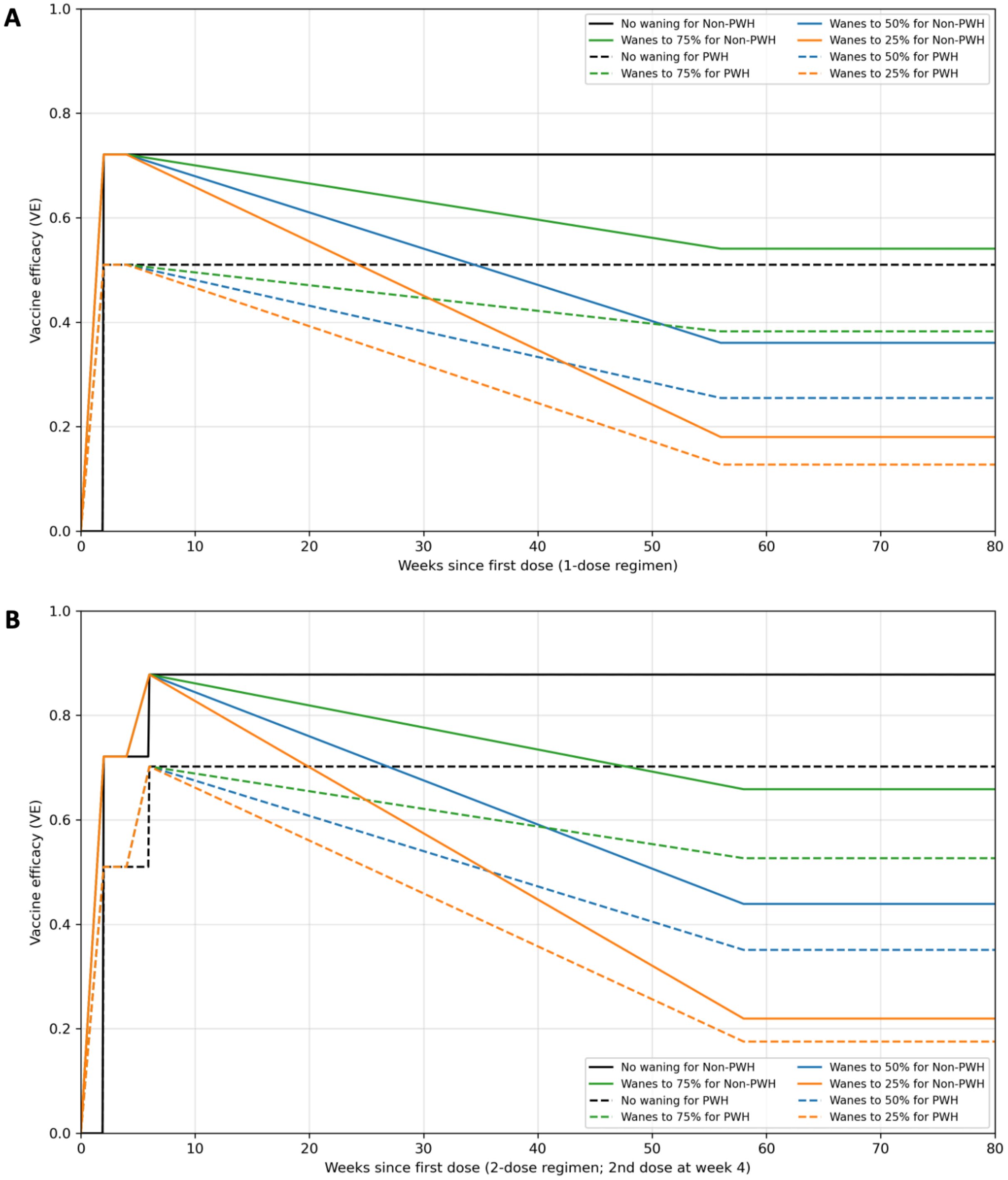

Figure S22. Vaccine efficacy over time by waning scenario and HIV status. (A) One-dose regimen. (B) Two-dose regimen. For the one-dose regimen (Panel A), vaccine efficacy increases linearly from zero to peak over 2 weeks following the first dose, remains at peak for 2 weeks, then wanes linearly over 52 weeks to a target fraction of peak efficacy. For the two-dose regimen (Panel B), vaccine efficacy follows the one-dose trajectory through week 4, then increases linearly over 2 weeks (weeks 4–6) following the second dose to reach full two-dose efficacy, and subsequently wanes linearly over 52 weeks (weeks 6–58) to a target fraction of peak efficacy. Solid lines represent HIV-negative individuals; dashed lines represent people with HIV (PWH). The base case assumes waning to 50% of peak efficacy (blue);

sensitivity analyses explore waning to 25% (orange) and 75% (green) of peak efficacy. The no-waning scenario (black) maintains constant efficacy after the initial development period as a reference comparison.

8. In the microsimulations, do all importations result in infected individuals at the onset of symptomatic infection? If so, could this lead to an underestimation of importations (by missing importations with partially elapsed symptomatic phases)? It may be worthwhile to examine the consequences of importations arriving at different points within the symptomatic infection (e.g., some arriving at the start of the infection, others halfway through, etc.).

We input all the introductions as individuals at the onset of symptomatic infection and already diagnosed with mpox as we wanted the model to not count the introductions as new cases but rather focus on the secondary cases to better capture the downstream effect of introductions. In order to explore the impact of this decision, we repeated our main analyses by both (1) assuming that introductions were at the onset of symptomatic infection but not yet diagnosed and (2) assuming that introductions come into the model both asymptomatic and undiagnosed, which allows importations to be introduced in different health states in our model. The reproduced Main Figures 5 are shown below:

First we assume that importations are symptomatic but not yet diagnosed:

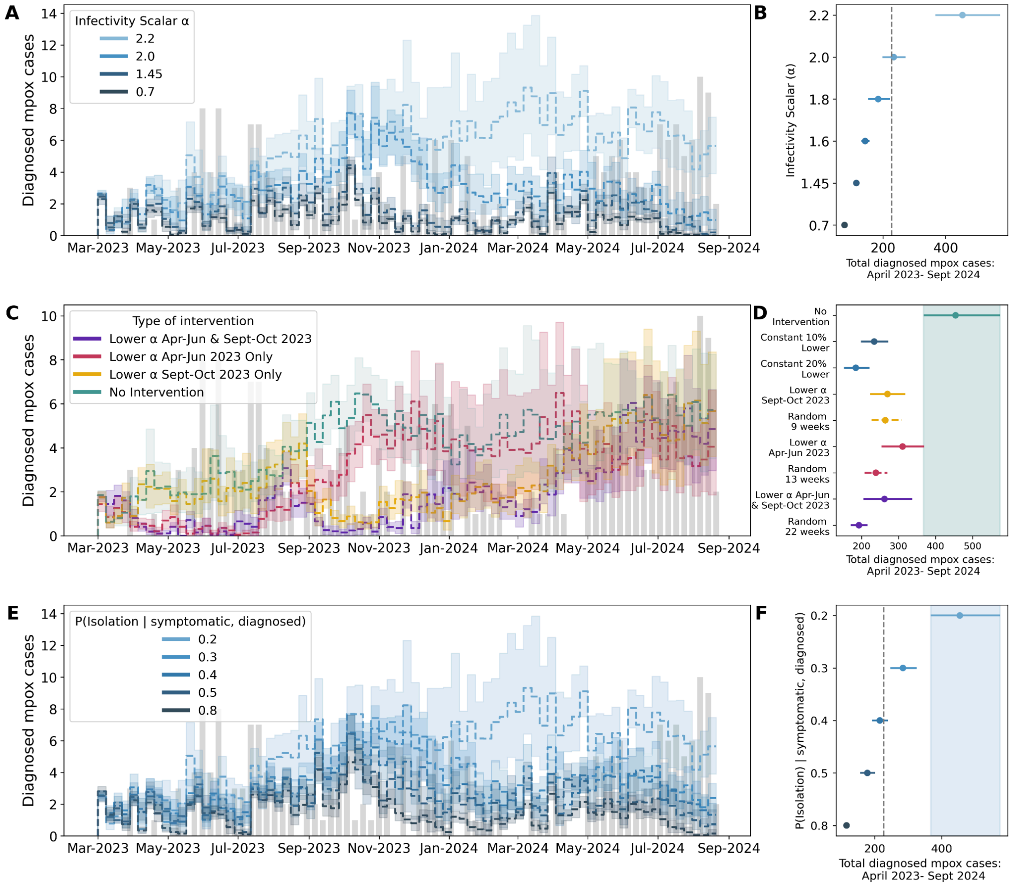

Figure S15: Inputting importations as symptomatic but undiagnosed. Here we repeat the analysis in Main Figure 5 but rather than assume that viral introductions enter the model as symptomatic and diagnosed, they enter as symptomatic and undiagnosed, allowing them to contribute longer to secondary infections before possibly becoming diagnosed.

Then we assume that importations are asymptomatic and undiagnosed:

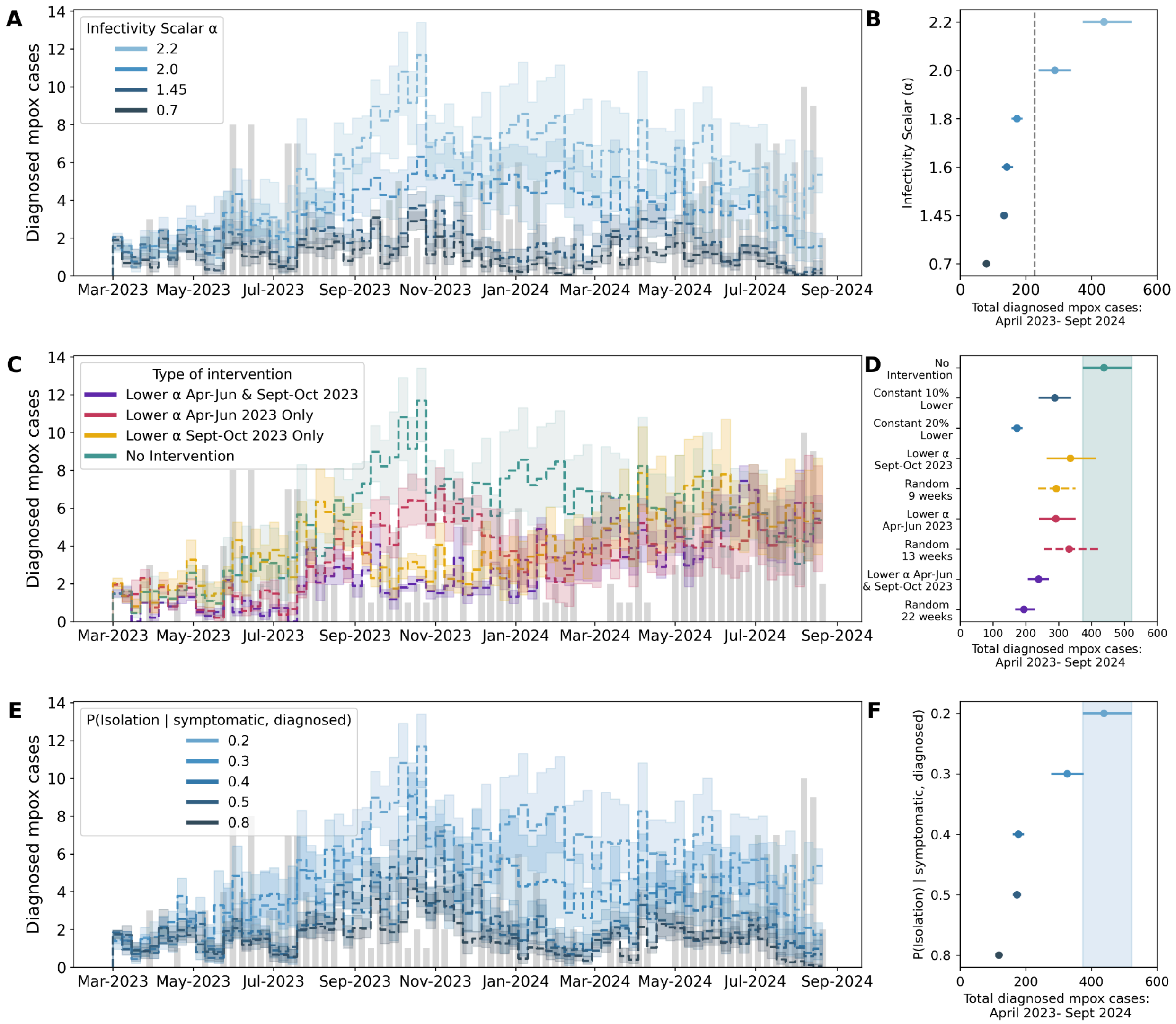

Figure S16: Inputting importations as asymptomatic and undiagnosed. Here we repeat the analysis in Main Figure 5 but rather than assume that viral introductions enter the model as symptomatic and diagnosed, they enter as asymptomatic and undiagnosed, allowing them to contribute longer to secondary infections and transition into being both symptomatic and diagnosed.

As you can see from these figures, the conclusions are highly similar. When importations are both asymptomatic and undiagnosed, we see that the the model assumes a slightly lower alpha (Panel A-B: between 1.8 and 2.0 instead of between 2.0 and 2.2 as in our main figure) showing that since importations are allowed to contribute slightly more time in the model, a lower increase in sexual behavior is needed to recapitulate empirical trends. These results continue to support the conclusion that sexual activity returned to baseline levels in 2023-2024.

The above figures have been included in the manuscript as supplementary figures S15-16 and we have modified the results section to include “While we input introductions in our base model as being both diagnosed and symptomatic (See *Methods),* inputting introductions as either

asymptomatic or undiagnosed still support a return to near baseline sexual activity (Fig S15-16).”

9. Since LAC often sequenced >50% of cases, it could be useful to repeat the analysis represented in Fig. S8A under multiple assumed sequencing coverages and contextualize the results accordingly. Also, please state what the black cross represents in the legend.

Below you will find Fig S8A (now Fig S11A) repeated assuming multiple sequencing coverage. We included values ranging from 1.5% to 50% sequencing probability. While assuming a low probability of sequencing decreases the likelihood of very low R with a high dispersal parameter *k*, prior studies have found the *k* for mpox in the US to be at or below 0.35. At every studied sequencing probability, our results show that it is highly probable to observe a cluster of size 16 among 64 clusters with an R significantly below one, further supporting our conclusions that the true R during 2023-2024 in LAC is below 1.

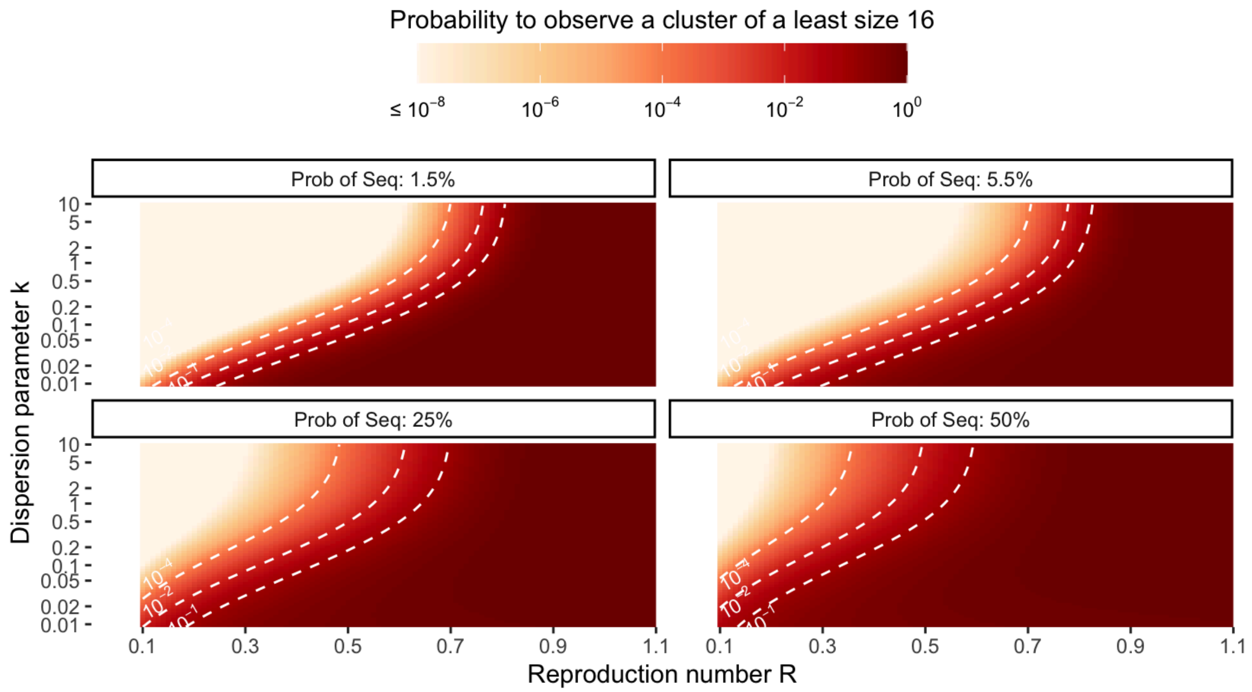

Minor comments:

1. Fig. S3: please consider using matching y-axis limits for the two bottom panels. Could have insets that zoom in on low values.

Thanks for catching that discrepancy. The bottom two panels of Fig S3 now have identical y axes as seen below:

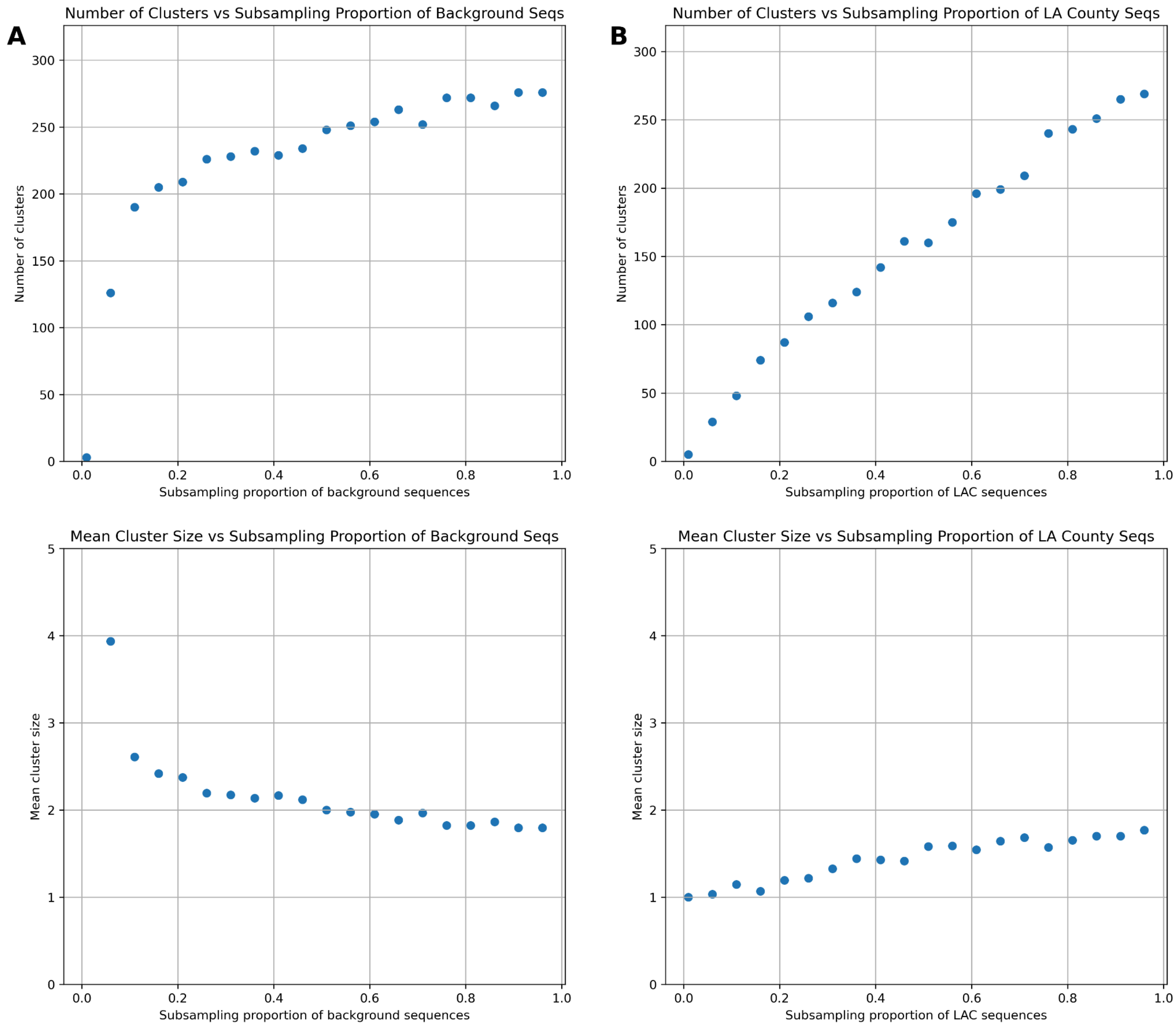

2. Fig. S10: did the authors mean blue, rather than yellow?

Thanks for catching that! That sentence has been updated to “The left column shows the true percentage in *black* with the estimated percentage in *blue* (showing the 95% HPD intervals).”

3. Fig. S16: please consider including what the different arrows and abbreviations represent in the legend, as in the original figure in Liang et al.

We thank the reviewer for this helpful suggestion. We have revised the model schematic (now Figure S21) to include a detailed legend that explicitly defines all arrows (e.g., transitions vs. viral introductions) and abbreviations (e.g., Iso, Trt). We have also expanded the figure caption to ensure the logical flow is clear and self-explanatory.

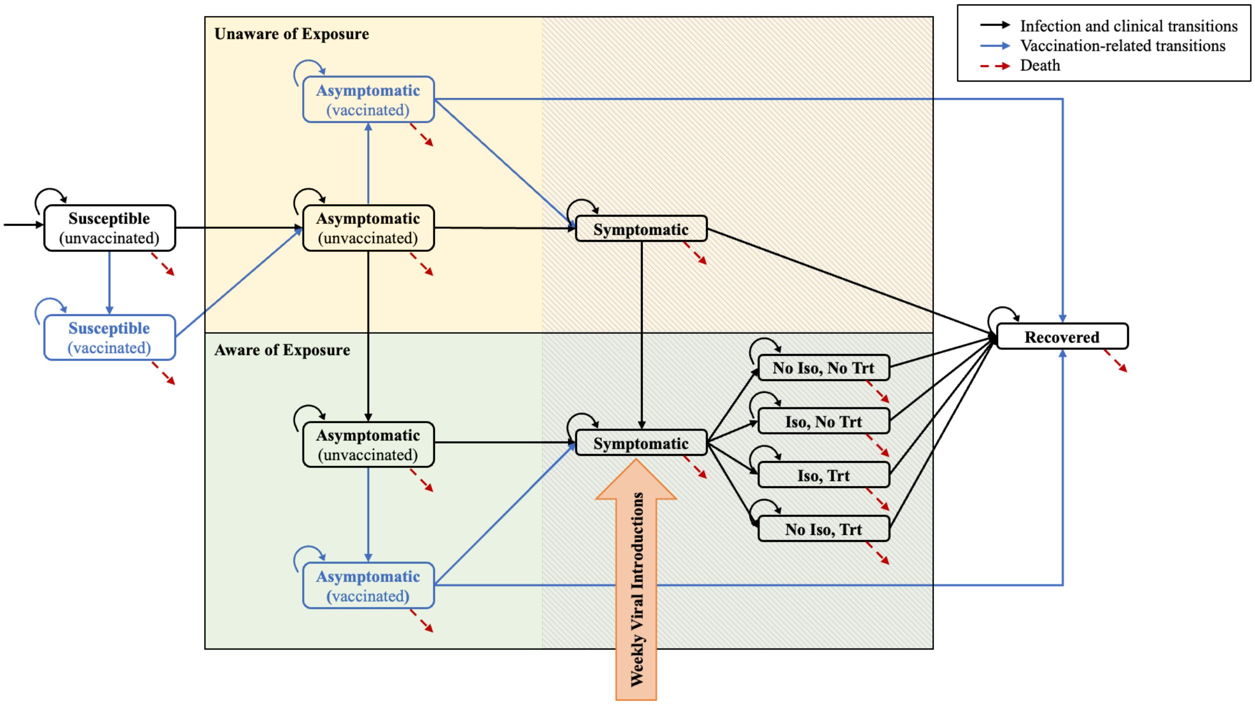

Figure S21. Schematic of the individual-based microsimulation model for mpox transmission dynamics in LAC, incorporating phylodynamics-informed viral introductions. The model simulates weekly transitions through infection, vaccination, diagnosis, isolation, treatment, and recovery. Individuals enter as susceptible and, upon infection, progress through an asymptomatic stage before becoming symptomatic. Transmission occurs when symptomatic individuals expose susceptible partners through sexual contact. Awareness status (yellow vs. green regions) drives care-seeking behaviors. Individuals Aware of Exposure (green) actively seek protection; they have a high probability of receiving vaccine post-exposure prophylaxis (PEP) while asymptomatic, transitioning directly to recovery (blue arrow). Conversely, those Unaware of Exposure (yellow) receive vaccination at the same rate as susceptible individuals. Upon developing symptoms, all individuals enter the symptomatic phase (grey-patterned region). Within this phase, aware individuals are highly likely to be diagnosed and channeled into isolation (Iso) and/or treatment (Trt) pathways, whereas unaware individuals have a lower probability of diagnosis and typically recover without intervention. Viral Introductions (orange arrow) represent phylodynamics-informed imported cases, which enter the model directly into the symptomatic, aware state. These cases contribute to ongoing transmission but are not counted as locally acquired diagnoses in LAC. All individuals eventually exit via recovery or non–mpox-related death (red dashed arrows). Adapted from Liang et al. (18)

(Remarks on code availability)

I have briefly reviewed the code; it appears thorough and reproducible. The code does appear to provide enough instructions to replicate much of the results, but I have not attempted to reproduce the analysis myself.

Reviewer #2 (Remarks to the Author):

Summary

The manuscript “Viral introductions and return to baseline sexual behaviors maintain low-level mpox incidence in Los Angeles County, USA, 2023-2024” presents results from two years of the mpox epidemic in Los Angeles County derived from both phylodynamic and microsimulation analysis. The authors find strong evidence that the mpox epidemic is currently driven primarily by importations rather than local transmission, and I appreciated the integration of epidemiological and phylodynamic lines of evidence. My main concerns are with the calibration of the microsimulation model and the simulated intervention scenarios, but I believe these concerns can be addressed in a revised version of the manuscript. Overall, the work presents a novel view of recent mpox transmission relevant to many cities in the United States, and I applaud the authors on their comprehensive undertaking!

We thank the reviewer for the thoughtful comments and suggestions!

Major Comments

My primary concern is with the calibration of the microsimulation model to the case data. The alpha parameter controls a large portion of the transmission dynamics and thus far the authors have calibrated it in an ad-hoc manner (e.g. letting it vary during 3 time points of the epidemic), so conclusions drawn from the microsimulations need to be qualified. I have two key concerns:

The authors note: “It showed, however, that without viral introductions into the county, mpox incidence would have been expected to drop to zero by March 2023,” in reference to the orange line in Figure 1A, but the authors had to alter alpha to match both the increase and decline in the epidemic. One could likely match the subsequent dynamics (early 2023 onward) through a model where alpha was increased to put the reproduction number fluctuating near 1 rather than adding in the importations). This also relates to the conclusions from Figure 1D (e.g. “With the same model parameters calibrated after August 2022, the model required a large number of mpox introductions (> 5 per week) to maintain ongoing transmission similar to the empirical number of cases (Fig. 1B).” That sentence is correct, but the authors suggest these results as evidence for importations being important to the subsequent dynamics, even though they do not compare it to model simulations where alpha during that time period is roughly calibrated to the case counts. If the authors would like to make the claim from these data, they should compare it against a counterfactual where the transmission pressure (alpha) was the primary driver of the dynamics and assess model fits between the two. Alternatively, the phylodynamic evidence is quite strong for the importance of importations for sustaining transmission, so I suggest the authors focus more on that for those conclusions rather than the microsimulation work presented in figure 1.

We apologize for some confusion. In this analysis, alpha is actually kept constant throughout our base cases simulation period (March 2023 - October 2024) and does not vary throughout the time period of interest except under counterfactual scenarios. Variation in alpha only occurred during the

calibration phase of the baseline microsimulation model found in Liang et al (July 2022 - March 2023) and in the hypothetical intervention scenarios evaluated in this present study (Fig 5C), which may have led to this misunderstanding. We have clarified this distinction explicitly in the revised manuscript and have elaborated much more in our response to the comment below this one.

We have also restructured the manuscript for clarity (as also suggested by Reviewer 1) by focusing on the phylodynamic evidence at the beginning and not on the microsimulation evidence for importation. The manuscript structure now goes from general mpox epidemiology in LAC, to our phylodynamic analyses, to our phylodynamics-informed microsimulation work.

We also explored the potential for our microsimulation model to recreate empirical mpox dynamics via only alpha and without importations as suggested:

Alpha of 2.2 (baseline sexual activity) with no viral importations:

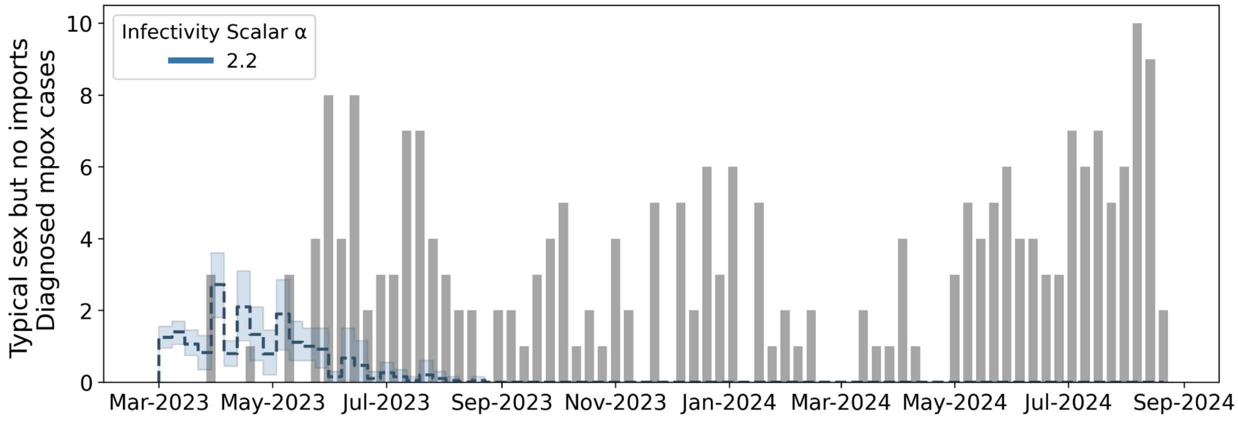

Above, we model the improbable scenario of sexual behavior rebounding immediately from epidemic levels (alpha of 0.7) directly back to baseline in March 2023 (represented by increasing the alpha to 2.2). We believe this to be a likely upper bound as prior work found that greater than 20% of MSM in North America maintained behavioral adaptations to the 2022 epidemic through May 2023 (https://www.thelancet.com/journals/laninf/article/PIIS1473-3099(24)00531-0/fulltext). As seen above, even assuming a rapid return to sexual behaviors in March 2023, the stochastic model is unable to recapitulate empirical case trends without the influence of importations. This figure has been added to the manuscript as Fig. S13:

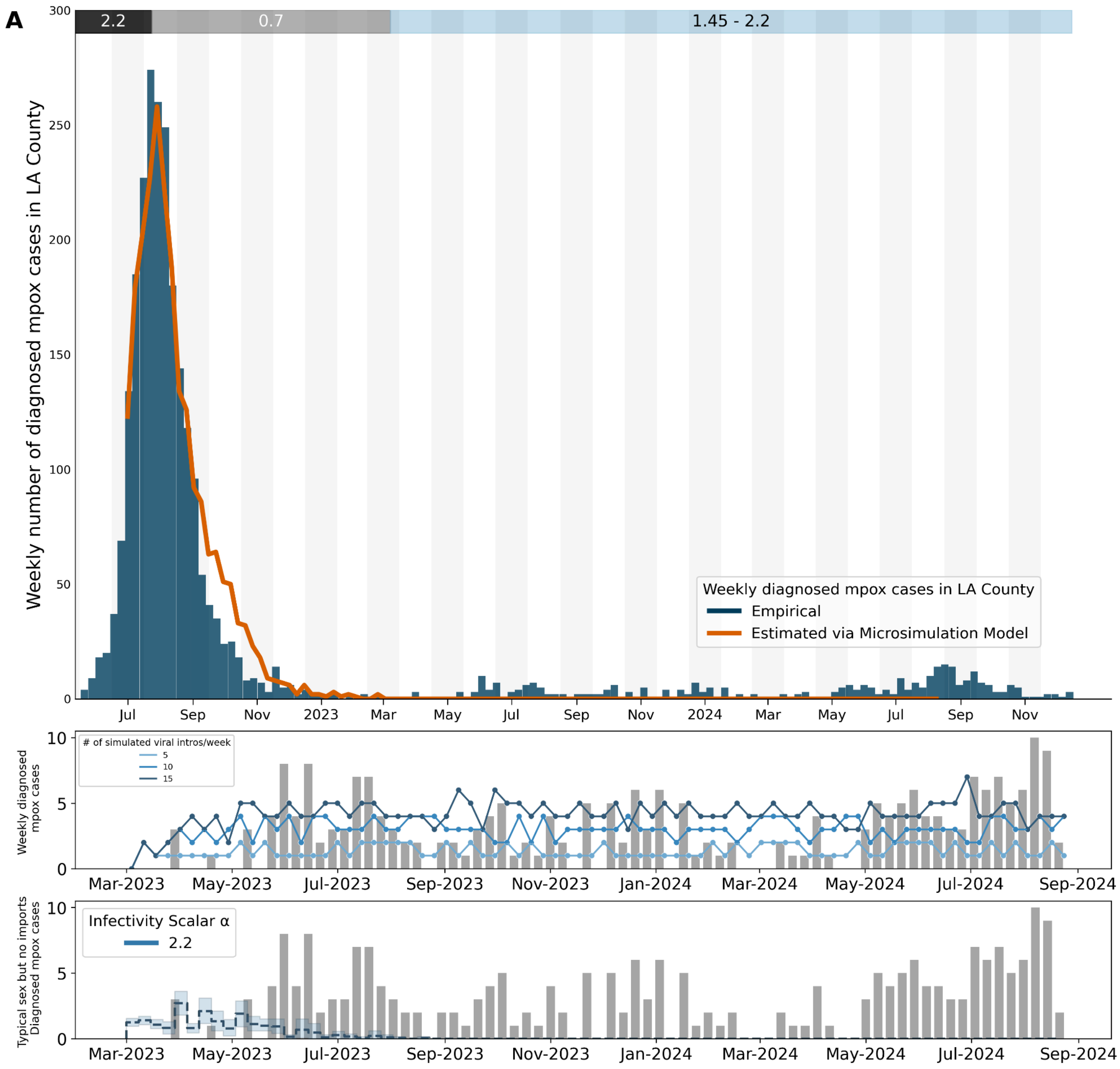

Figure S12: Infectivity Scalar (α) during 2022 mpox outbreak in LA County. **(A)** Main figure shows the weekly number of diagnosed mpox cases in LA County from June 2022 through December 2024 (blue) with the number of diagnosed mpox cases simulated via our microsimulation model without viral importations overlaid in orange. The bars in the top of the figure are a visual representation of the periods of time for which alpha was calibrated. Panel **B** shows the daily number of diagnosed mpox cases (blue) with the daily number of mpox sequences collected in LAC overlaid (purple) from June 2022 to December 2024. Panel **C** shows the weekly number of mpox vaccinations that were administered in Los Angeles County from June 2022 to October 2024 divided between the number of first doses (pink) and second doses (teal) given. The grey bars represent the initial model calibration for the epidemic period and the blue bar shows the period of interest for this study. **(D)** Scenario analysis of the impact of a constant force of viral introductions on our microsimulation model from March 2023 to September 2024. The empirical mpox case counts are represented by the gray bar chart while the simulated cases are represented by the blue point and line charts with an increasing number of viral importations per week. Panel **E** represents the counterfactual scenario of a complete revision to baseline sexual activity in March 2023 (represented by an alpha of 2.2) but without any introductions into the model

For Figure 5, the calibration becomes much more important. The authors “calibrate” alpha to the case counts, but ultimately their best fit value of 2.0 does not match the case time-series well (though I admit it does get roughly the cumulative case count correct). It is clear that alpha must fluctuate over time even during this time period to recreate the results, for example, potentially alpha needs to be increased in the Spring of 2023 to recreate the summer bump in cases, and then potentially decreased again. As it stands, the importation surge in the summer of 2023 is leading to increased case counts in fall/winter of 2023 rather than in the summer of 2023 leading to a case/simulation mismatch. Based on the data available to them, I would suggest that the authors implement a more systematic calibration of alpha to more closely align the case counts from the microsimulation to the data itself. If this is computationally infeasible, then the authors need to very strongly justify their manual calibration procedure and I would suggest allowing alpha to vary more widely over time to get a closer match with the data. This is particularly important for Figure 5, because the interventions that impact alpha are going to have very different impacts on case counts depending on how the timing of those interventions align with the fluctuations of alpha and the imported case seasonality.

We appreciate the reviewer’s insightful comment. We agree that alpha, the infectivity scalar, may change in reality due to behavioral shifts or public health messaging. In the prior work (Liang et al.), alpha was calibrated using a grid-search procedure and adjusted once from 2.2 to 0.7 to capture the pronounced peak and subsequent sharp decline in mpox cases during mid-2022. This reflected potential changes in behavior that influenced transmission.

After that change in mid-2022, alpha is held constant within each baseline simulation scenario and is varied only across scenarios rather than across time. We agree that, in reality, alpha could have had small changes over time during this time period (e.g., possibly in the Spring 2023, resulting in a summer bump of cases). However, we chose not to implement a finely time-varying alpha because the fluctuations in reported cases after March 2023 are relatively small and noisy. Calibrating a time-varying alpha would likely lead to model overfitting and also make it difficult to interpret intervention effects, as changes in alpha would be confounded with the intervention itself. Thus, at the cost of not precisely fitting the empirical case count data, we have assumed a constant alpha to better illustrate the effect of importations and interventions. Our results should be interpreted within that assumption, and we have clarified that in the manuscript (see below).

In a small subset of intervention scenarios, alpha is reduced during predefined time windows to represent short-term reductions in sexual activity associated with public health interventions. These changes are imposed mechanistically to represent hypothesized behavioral responses and are not calibrated to short-term variations in case counts.

To respond to the reviewer’s concern, we have revised the calibration section in the manuscript to clarify the one-time change in alpha in the previous model, to explain why alpha is held constant within each scenario in the present study, and to describe when and why alpha is

varied in specific intervention scenarios. The revised text appears in the Microsimulation Model section, under *Calibrating and Estimating the Infectivity Scalar* (α) subsection, and reads as follows:

"In the initial model setup, α was calibrated through a grid search across 2.1 to 2.5, in combining with the asymptomatic infections in the initial cohort, and was set to 2.2. This value aligns with empirical trends from the early phase of the mpox outbreak in LAC, reflecting the high transmission levels observed prior to August 2022. Following the pronounced peak and subsequent sharp decline in reported cases during late summer and early fall of 2022, α was recalibrated using a grid search across 0.6 to 0.9, and set to 0.7, which likely reflects the impact of enhanced public health guidelines and changes in public behaviors during that period. Details of the grid-search procedures used to determine these α values are provided in Liang et al. (18). This one-time adjustment allowed the model to capture both the elevated transmission in mid-2022 and the sharp reduction afterward."

"In the present study, α is held constant within each baseline simulation scenario and is varied only across scenarios within the empirically supported range of 0.7 to 2.2. We did not implement continuous or weekly time-varying calibration because empirical fluctuations after March 2023 are small and noisy. However, in a subset of intervention scenarios, α is intentionally reduced during predefined time windows to represent short-term reductions in sexual activity associated with public health interventions. These changes are imposed mechanistically to reflect hypothesized behavioral responses rather than calibrated to short-term fluctuations in reported case counts. This approach preserves the interpretability of intervention effects while avoiding overfitting to transient noise in the surveillance data."

We have also clarified that our results should be interpreted within the context of a constant-alpha assumption in the manuscript as follows in the Discussion section:

"Microsimulation results also rely on the assumption that α remains constant after mid-2022. In reality, transmission pressure and sexual activity may have fluctuated during the analysis period, creating small variations in weekly case counts. However, we lacked reliable data on these short-term behavioral changes, and adopting a time-varying α risked overfitting transient noise in the surveillance data. Consequently, we assumed a constant , which means the model does not match empirical case counts exactly week by week, although it successfully captures the overall epidemic trajectory."

I like the principle behind the idea for Figure 5C and D, but based on the authors results I am having trouble understanding the conclusions. Looking at the interventions it appears that randomly timing interventions is always better or equal to timing the intervention during the period of high importation pressure. Based on Fig S13, I think this is because the random interventions occur throughout the whole time period whereas the targeted interventions occur only in 2023. If this is the case, the authors should instead run the simulations where the

random weeks are chosen randomly only for 2023 as a comparison rather than being spaced over the whole time period. If it's not because of that, then the authors should think deeply about why the random interventions are having a disproportionately large impact as that may provide an interesting public health strategy to reduce future case counts.

We thank the reviewer for noticing this! That was in fact the case: the random weeks were occurring throughout the whole time period rather than just being focused in 2023 like the targeted interventions were. We have rerun our analyses with the random weeks just being focused in 2023. You can see by the updated Main Figure 5C-D and Supp Figure S13 below.

New main Fig 5:

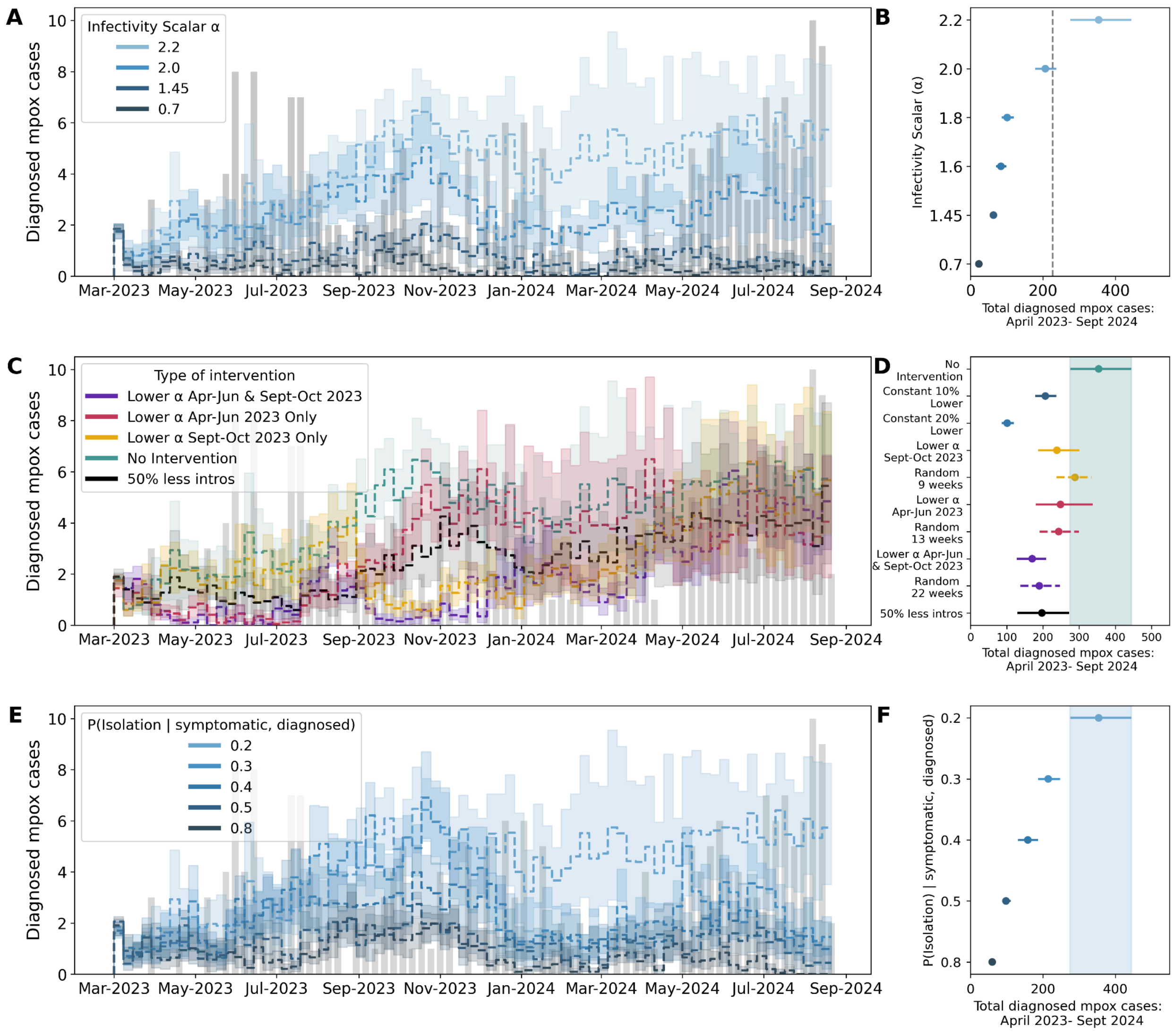

Figure 5: Factors maintaining mpox prevalence and modeling counterfactual public health interventions. After parameterizing our microsimulation model with the number of viral importations estimated via phylodynamics, **(A, B)** we explored the α that best explains the empirical weekly number of diagnosed mpox cases (gray bars). Line graphs represent the mean weekly number of mpox diagnoses simulated using increasing α. Given the non-constant

pattern of viral introductions seen in the phylodynamic analysis, we tested different counterfactual scenarios of public health interventions during specific time periods **(C, D)** represented by lowering the α to 0.7 while keeping the α at 2.2 during the remaining time. The bold yellow, red, and purple lines represent the simulated weekly number of diagnosed mpox cases under phylodynamic-informed interventions. The black line represents a 50% reduction in introductions. In **D,** we also compared the impact of lowering the α for the same random number of weeks as each specified intervention as well as the impact of constantly lowering the α throughout the entire time period by about 10% and 20% to simulate a constant low-effectivity intervention. The green area represents the upper and lower bounds of the "No Intervention" scenario. We also tested the effect of increasing the probability of isolating upon a symptomatic individual receiving a positive mpox diagnosis on the simulated number of diagnosed mpox cases **(E, F).** In **F,** the light blue area represents the bounds of the base model scenario with an α of 2.2. In **A, C, E**, the grey bars represent the empirical number of mpox diagnoses in LAC. We calculate the uncertainty of our microsimulation results via bootstrapping to estimate 95% uncertainty intervals for each weekly simulated estimate. For **B,** the dashed line represents the total empirical number of diagnosed mpox cases from April 2023 through September 2024

Compared to the original Main Fig 5, panel D shows that targeted and random lowering of introductions have pretty similar impact on the cumulative number of diagnosed cases by the end of the two year period, with the targeted approach having a slightly lower median number of cases. To understand why, we can see updated Supp Fig 13 (now Supp fig 18) below:

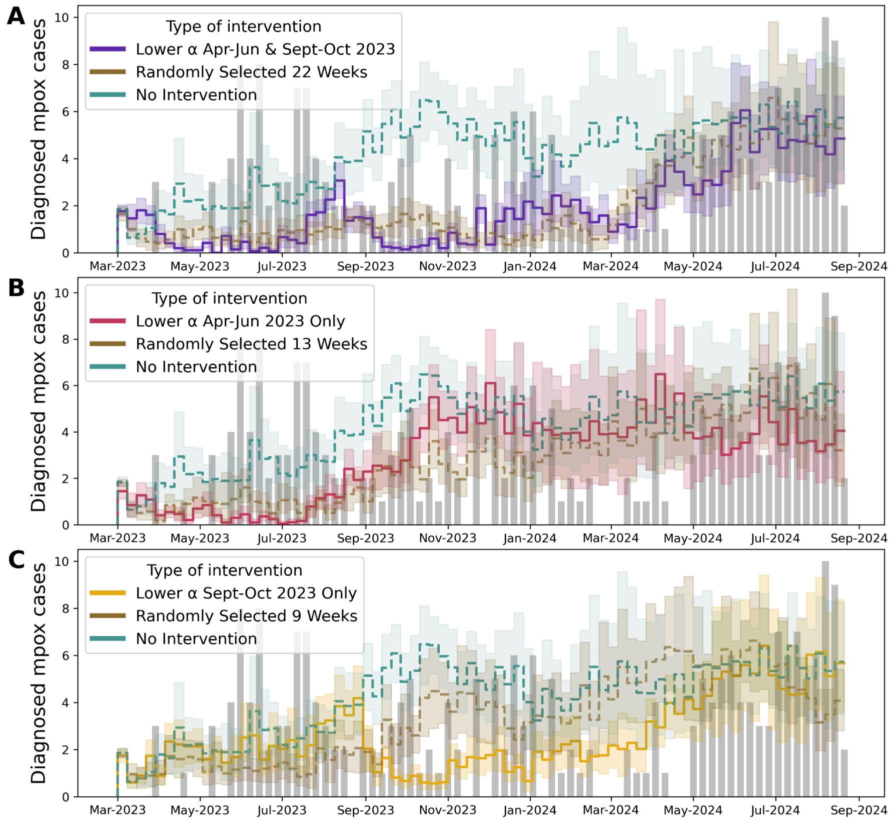

Figure S18: Factors maintaining mpox prevalence and modeling counterfactual public health interventions with statistical tests. (A-C) Given the non-constant pattern of viral introductions seen in the phylodynamic analysis, we tested different counterfactual scenarios of public health interventions during specific time periods represented by lowering the α to 0.7 while keeping the α at 2.2 during the remaining time. The bold yellow, red, and purple solid lines represent the simulated weekly number of diagnosed mpox cases under phylodynamic-informed interventions. To test for non-specific effects, we also reran our microsimulation model by randomly selecting the same number of weeks in 2023 as our phylodynamics-informed interventions to

lower the α to 0.7 (brown dashed lines) as well as a simulation without any interventions. (green dashed lines) In all plots, the grey bars represent the empirical number of mpox diagnoses in LAC.

Supp Fig S18 shows that by the end of the 2 year period, the number of diagnosed cases between the random and targeted approaches is highly similar. We believe that this is due to the fact that dynamics in LAC are highly driven and dependent on importations that create “entangled stutter chains” rather than ongoing local transmission. This means that while controlling importations is an impactful strategy to control cases, it must be done consistently and frequently, rather than just one-off. This also explains why our prior approach of randomly lowering introductions throughout the entire 2 year period tended to be more impactful on the cumulative number of cases than a strongly targeted approach in 2023. This is highlighted in Main Figure 5 under the “Constant lower 20%” scenario which shows that a constant intervention, even if low efficacy, is more impactful than strongly targeted approaches in the absence of completely being able to eliminate viral introductions.

We have updated the figures appropriately in the text and added “ We also found that having a constant intervention was often more impactful than targeted interventions, suggesting a transmission dynamic dominated by introductions rather than continued local transmission.”

Related to my above comment, I am a bit puzzled at the choice of interventions the authors explore in Figure 5. First, I was curious why the authors did not explore what strategies are necessary to push mpox case counts down to zero as much as possible. If that truly is the public health goal it would be helpful to know what needs to happen (e.g. how low does alpha need to be to get there?). If that isn’t the goal, then I would suggest the authors more clearly state what the public health objectives would be in these scenarios (e.g. why they only consider scenarios where alpha is lowered to the extent they lower it in the simulations). Second, and potentially more importantly, in the previous results before this figure the authors showed decisively that the reproduction number is well below one and that case counts are driven primarily from importation-associated transmission. If that’s the case, it seems non-optimal to focus public health interventions on reducing local transmission, and it would be useful for the authors to compare with a different control strategy that might be more impactful (e.g. reducing importations). If you can reduce importations by 50% how does that compare in cumulative case counts to the strategies where you are simply reducing transmission? How much would you need to reduce importations to get the cumulative case counts near zero? There are a number of ways one could imagine public health officials focusing their efforts on reducing importations that would be different than how they might reduce transmission (alpha), and it would likely be helpful to run this analysis to help them allocate their resources appropriately (my hypothesis would be that one could get a better bang for the buck focusing on importations, but I’m curious to see what the modeling results suggest).

While mpox elimination in LAC is always the aspiration, the goal of our current work after discussions with our public health partners was to explore feasible and plausible strategies for mpox control. While completely eliminating importations (as seen in the response to the comment above) or stopping sexual activity would result in locally-acquired mpox cases eventually going to zero, such strategies are implausible to implement. We chose to explore alpha values from 0.7 to 2.2 as we believe they represent upper and lower bounds of sexual activity: as seen in Fig S12, an alpha of 0.7 represents the reduced sexual activity during the peak of the 2022 mpox epidemic where significant behavior modification among queer communities was documented; additionally, an alpha of 2.2 describes higher than baseline sexual behavior during June-July which includes major LGBTQ+ events. Values representing sexual activity below or above that, respectively, we believe, would be unfeasible in a typical public health context (outside of statewide stay-at-home orders as seen during the COVID-19 pandemic). We have clarified these choices and assumptions in the text as follows:

“Notably, we bounded our modeled interventions in the realm of plausible scenarios that could be enacted by public health agencies. While complete elimination of viral importations (as seen in Fig S12E) or complete cessation of sexual activity would likely result in elimination of locally-transmitted mpox, such strategies are implausible to implement.“

Additionally, we'd like to thank the reviewer for the suggestion to explore the impact of having 50% less importations – it's an idea we were excited to explore! Below you'll find our exploration of how reducing importations by 50% would impact our simulations with varying levels of alpha. As seen in panel A, our model has a difficult time recapitulating similar case numbers even with a higher alpha than our base model, showing the significant impact of the reduction of importations. We have included this analysis as Fig. S19 .

50% less importations:

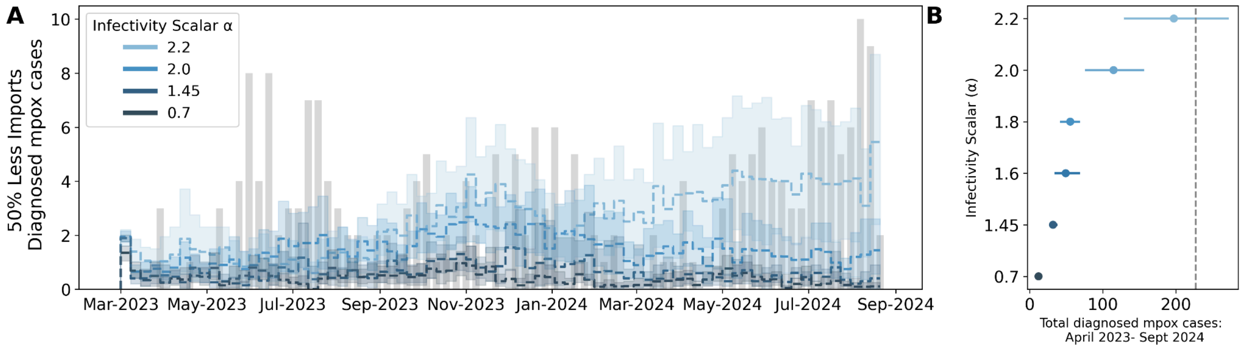

Figure S19: Infectivity Scalar with half as many phylodynamics-informed viral introductions. To test the impact of interventions that targeted introductions, we halved the number of introductions, reran our microsimulation model, and explored the Infectivity Scalar that best explains the empirical weekly number of diagnosed mpox cases (gray bars). Line graphs represent the mean weekly number of mpox diagnoses simulated using increasing . Each weekly estimate represents the average of 10 independent iterations of our model. The dashed line in **B** represents the total empirical number of diagnosed mpox cases from April 2023 through September 2024.

We have also included this scenario in our reworked main figure 5C-D (black lines) as seen below. Given that dynamics in LAC are heavily driven by importations, we see that a 50%

reduction in importations results in about a 50% reduction in the cumulative number of diagnosed mpox cases in the two years studied (Fig 5D). Together, these scenarios provide our public health partners with various options with which to achieve significant reductions in the local levels of mpox.

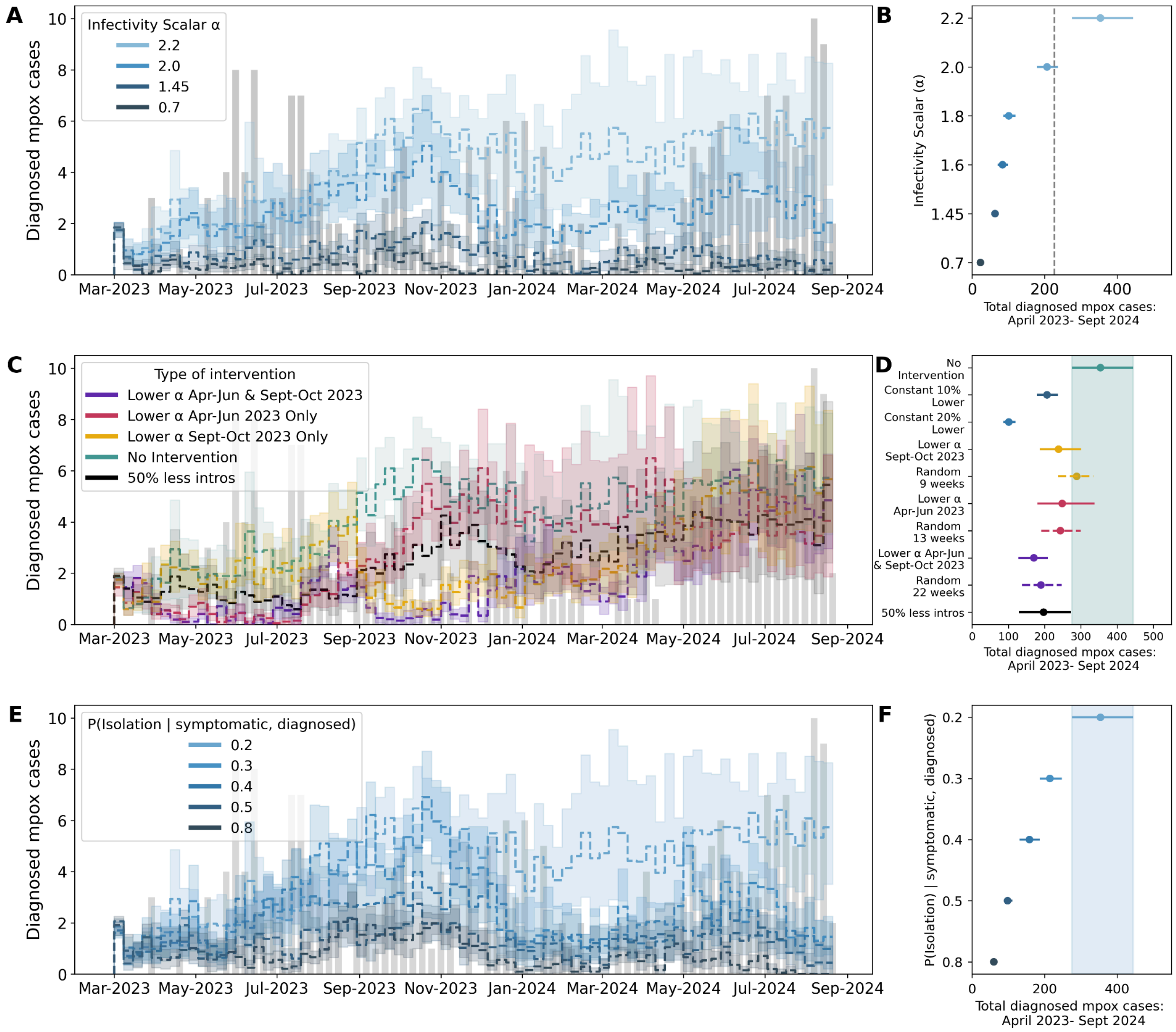

Figure 5: Factors maintaining mpox prevalence and modeling counterfactual public health interventions. After parameterizing our microsimulation model with the number of viral importations estimated via phylodynamics, **(A, B)** we explored the α that best explains the empirical weekly number of diagnosed mpox cases (gray bars). Line graphs represent the mean weekly number of mpox diagnoses simulated using increasing α. Given the non-constant pattern of viral introductions seen in the phylodynamic analysis, we tested different counterfactual scenarios of public health interventions during specific time periods **(C, D)** represented by lowering the α to 0.7 while keeping the α at 2.2 during the remaining time. The bold yellow, red, and purple lines represent the simulated weekly number of diagnosed mpox cases under phylodynamic-informed interventions. The black line represents a 50% reduction in introductions. In **D,** we also compared the impact of lowering the α for the same random number of weeks as each specified intervention as well as the impact of constantly lowering the α throughout the entire time period by about 10% and 20% to simulate a constant low-effectivity intervention. The green area represents the upper and lower bounds of the "No Intervention" scenario. We also tested the effect of increasing the probability of isolating upon a symptomatic individual receiving a positive mpox diagnosis on the simulated number of diagnosed mpox cases **(E, F).** In **F,** the light blue area represents the bounds of the base model scenario with an α of 2.2. In **A, C, E**, the grey bars represent the empirical number of mpox diagnoses in LAC. We calculate the uncertainty of our microsimulation results via bootstrapping to estimate 95% uncertainty intervals for each weekly simulated estimate. For **B,** the dashed line represents the total empirical number of diagnosed mpox cases from April 2023 through September 2024

It would be great if the authors could include more of a discussion on the fluctuation of viral importations they identified. Is this linked to epidemic waves in other regions that the authors discuss (e.g. NYC, Chicago, California, etc)? Is it linked to specific events (e.g. pride)? Something related to the seasonality of travel? The authors note in the discussion “Of note, both sexual behavior and travel vary by season” and mention how summer travel is particularly large, but this wouldn’t seem to explain the bump in importations in Feb-April of 2024 or the pattern of monthly importations from Figure 4B.

We added the following to the discussion:

“We demonstrate that importations of mpox into LA county follow a roughly seasonal pattern with peaks in Aug-Sept and March (Figure 4), but the importations at these times do not appear to stem from the same geographical locations (Figure 1b). While we suspect return from summer (Pride) and holiday travel within the most impacted subpopulations for mpox infection may play a role in these peaks, additional work using data from the International Air Transport Association (6) and case-specific metadata to determine the scale of travel to Los Angeles at these times could provide more mechanistic detail underlying this pattern. Previous work shows that nearly 20% of MSM travel with a primary goal of engaging in anal sex without a condom (41), a potentially useful population for mpox outreach. Seasonal patterns in STIs in the US also show peaks in March and August, with the authors suggesting travel relating to summer vacation and university spring breaks as a potential cause, although the data are now over 20 years old (42). University-related travel may likewise provide an opportunity for mpox vaccination outreach if younger individuals are newer to sexual networks and have not yet received a complete vaccine series and are therefore immunologically naive (43)”

The authors reference Ref 18 for almost all of the details about the microsimulation, but it would be helpful if some of the key components of the model were included in this paper or the associated supplemental information rather than having to flip back and forth between two papers. For example, one could include the overall model architecture or diagram (e.g. the process and measurement models), the table of parameters associated with that paper, initialization values, simulation timelines, etc.

Thank you for this suggestion. We expanded the Supplementary Information to include key components of the microsimulation from the previous model and clarifications specific to the current analysis. Supplementary Table S1 now summarizes the key model inputs used in this study, including baseline initialization values and weekly transition probabilities drawn from Ref. 18 or updated using new surveillance data. We also revised Figure S21 and its caption to provide a clearer schematic of the model structure, including major health states, vaccination

pathways, diagnosis and treatment flows, and phylodynamics-informed viral introductions; the caption now explains all arrow conventions, box colors, and abbreviations to ensure the figure is self-contained. In addition, we clarified the simulation timeline. Revised sentences added to Microsimulation Model section, Model Overview subsection:

"The original model was run from July 2022 to March 2023 to generate a realistic baseline population, which served as the starting population for the present analysis covering March 12, 2023 to October 27, 2024 (85 weeks). The model schematic is shown in Fig. S21, and the key initial conditions and weekly transition probabilities are summarized in Table S1."

These additions provide essential model architecture and parameterization details within this manuscript while allowing Ref. 18 to remain the full methodological reference.

Parameter	Value	Source
**Initial Population (March 2023)**		
LAC total MSM population	263,322	*
Mpox symptomatic cases (Undiagnosed)	2	*
Mpox symptomatic cases (Diagnosed)	3	*
Individuals Recovered from Mpox	1,337	*
Mpox vaccinated (1st dose)	19,563	*
Mpox vaccinated (Fully vaccinated)	45,520	*
**Weekly Transition Probabilities**		
**Demographics**		
Weekly entry rate (fraction of current MSM)	0.0003	†
**Disease Progression (Natural History)**		
Mpox asymptomatic Symptomatic	0.50	†
Mpox asymptomatic Recovered (post-vaccination)	0.43	†
Mpox symptomatic Recovered (untreated)	0.28	†
Mpox symptomatic Recovered (treated)	0.50	†
**Diagnosis P(Mpox undiagnosed Diagnosed)**		
Asymptomatic (HIV-negative or Status Unknown)	0.01	†
Asymptomatic (HIV-positive, Aware)	0.10	†
Symptomatic (by age group) ‡	0.7 - 0.9	†
**Vaccination Uptake P(Unvaccinated Vaccinated)**		
1st dose (Susceptible or Unaware asymptomatic)	0.0018	§
1st dose (Aware asymptomatic)	0.95	†
2nd dose (Eligible) ‖	0.50	†
**Vaccine efficacy**		
1st dose and HIV-positive	0.51	†
1st dose and HIV-negative	0.72	†
2nd dose and HIV-positive	0.70	†
2nd dose and HIV-negative	0.88	†

* Derived from simulation outputs in Liang et al. (18)
† Parameters adopted from Liang et al. (18)
‡ Diagnosis probabilities by age group: 15–29 (0.8), 30–39 (0.9), 40–49 (0.8), 50–59 (0.9), 60–100 (0.7).
§ Calibrated to match observed vaccination trends in LAC.
‖ Eligible definition: individuals who received the 1st dose vaccination 4-6 weeks prior and remain susceptible or undiagnosed asymptomatic

Table S1: Key Model Inputs and parameters for the microsimulation. The table summarizes the initial population conditions for March 2023, weekly transition probabilities governing demographic turnover, disease progression, diagnosis, and vaccination uptake. Parameters marked with * are derived from simulation outputs in Liang et al (18); those marked with † are adopted directly from Liang et al (18); and those marked with § are calibrated to updated surveillance data.

Minor Comments

Line 170: remove errant "the."
This was addressed with the reorganization of the manuscript – thank you for catching this.

Line 182: should reference Fig. 1D.

This was also addressed with the reorganization of the manuscript – thank you for catching this as well!

Figure 5A: color and interval distinctions are difficult to differentiate; consider alternative schemes.
This is added jointly with the comment below.

Figure 5C: the figure may be clearer if the grey case bars are removed, since the main purpose is to compare scenarios rather than match to data.

Agreed that the main purpose is to compare scenarios rather than match to the data but we feel that the grey case bars in 5C helps anchor the simulations in a real world context. To make sure that they do not distract from the goal of the figure, we have made the grey bars much more transparent and have removed the dashed grey line representing "empirical case data" in Figure 5D. We have also lightened the grey bars for the other panels as well, which makes the interval distinctions in panel A and E more clear. And have removed the "alpha = 1.8" line in panel A to allow for more clear visualization of the remaining 4. We explored other color schemes to display the data in Fig 5A, but based on Makinlay's principles for graphical presentations of ordinal quantities, we focused on modulating color density as using increasing color density to represent increasing values has been found to be more effective than changing color hue (https://dl.acm.org/doi/10.1145/22949.22950) .

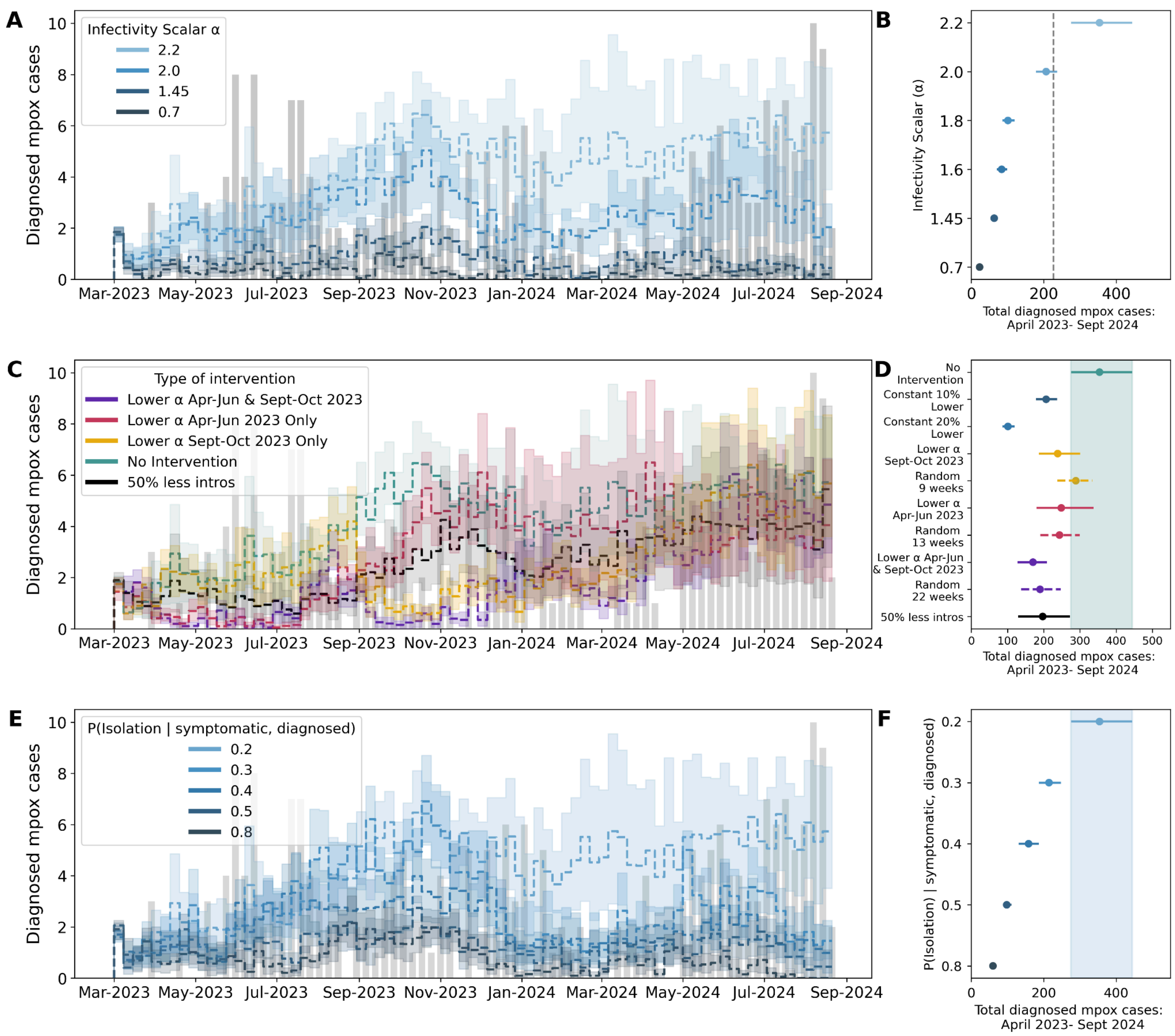

Reviewer #2 (Remarks on code availability):

I did not attempt to recreate the analyses, but upon my quick glance through the code it appears to be reasonably well organized and documented for reproducibility purposes.

Reviewer #3 (Remarks to the Author)

Parades et al., performed a phylodynamic analysis and simulation on mpox incidence in Los Angeles County, 2023-2024.
The study is well performed, the manuscript is well written and the figures are excellent.

Thank you for your kind words!

I have some comments to be addressed:

- Line 133, there is an extra point.

Fixed, thank you!

- Please report in the figure 1 legend the study period(from.. to ..).

The Fig. 1 legend now includes the study period for each panel : Main figure shows the weekly number of diagnosed mpox cases in LAC from *June 2022 through December 2024* (blue) with the number of diagnosed mpox cases simulated via our microsimulation model without viral importations overlaid in orange. Panel B shows the daily number of diagnosed mpox cases (blue) with the daily number of mpox sequences collected in LAC overlaid (purple) from *June 2022 to December 2024*. Panel C shows the weekly number of mpox vaccinations that were administered in Los Angeles County from *June 2022 to October 2024* divided between the number of first doses (pink) and second doses (teal) given.

-In the Model overview, there is no mention of importations. Do the authors think it could have affected the model?

The original model (Liang et al. 2024: https://academic.oup.com/ofid/article/11/Supplement_2/S137/7715945) was calibrated using data starting 5 weeks after the initial cases of mpox were reported in Los Angeles County. The original model assumed that local transmission dominated local mpox dynamics during the 2022 mpox epidemic which we confirmed to be an appropriate assumption in Paredes et al 2024 (https://www.cell.com/cell/fulltext/S0092-8674(24)00124-7). In 2023 and onwards, however, we believe this to be an unrealistic assumption, as evidenced by the model's inability to reproduce the local dynamics past March 2023 (Fig. S12A) and then confirmed by our subsequent scenario analysis adding in importations (Fig. S12D). This scenario analysis prompted us to inform the microsimulation model with the phylogenetics-informed importations that led to the more realistic model results in Fig 4.

- I suggest reporting the resulting phylogeny in the results (lines 947-948).

We have updated the results to include the following:
"To investigate transmission dynamics into LAC, we analyzed 497 mpox genomes sampled in LAC through December 12, 2024 alongside 7,362 contextual sequences from around the world by creating a time-resolved phylogeny using Nextstrain (Fig. S2, can be viewed interactively at https://nextstrain.org/groups/blab/mpox/allcladeIIseqs) (23)."

Figure S2 also shows a screenshot of the phylogeny and includes the hyperlinks as well.

- Considering the inclusion criteria of the genomes and the filter, I have concerns about the limitations of the local transmission. Since the inclusion criteria were very strict, I am afraid that a part of the introduction history is missing.

While our phylodynamic analyses will always be limited by the number of cases diagnosed and subsequently sequenced, our Nextstrain pipeline was designed to be as inclusive as possible, including every Clade IIb (since there were no Clade I mpox cases in LAC in 2023-2024) from 2022-2024. We excluded sequences with low quality as this is standard in most phylodynamic analyses as low sequence quality could artificially bias our results by distorting the natural genetic diversity with sequencing artifacts, ultimately leading to biased results. Outside of these quality control checks, we worked together with LADPH to ensure that the sequences included as part of LAC were truly from Los Angeles County to reduce the risk of misclassification bias. While this might represent a conservative approach to genome selection, it is done so to reduce the introducing bias into our results so that our introduction history reflects the lower bound of importations as we describe in the discussion. We state in the manuscript that “Given that phylogenies only capture successful introductions and that only a number of these introductions are sequenced, the phylodynamic-estimated number of introductions into LAC is expected to be a lower bound. “

- Were the genomes aligned?

The genomes were aligned using nextalign via Nextclade (https://github.com/nextstrain/nextclade). The methods state “A temporally-resolved phylogeny was created using a modified version of the Nextstrain (23) mpox workflow (https://github.com/nextstrain/mpox), which aligns sequences against the MK783032 (collection date: Nov. 2017) reference using nextalign (60), infers a maximum-likelihood phylogeny using IQ-TREE (61) with a GTR nucleotide substitution model, and estimates molecular clock branch lengths using TreeTime (62). “
The alignment can be recreated using the Nextstrain pipeline found in the manuscript’s github repo at https://github.com/blab/mpox-la/tree/main/mpox_build which creates the phylogeny found on https://github.com/blab/mpox-la/tree/main/mpox_build . The raw alignment file was not included due to file size limitations.

- Line 211. "...we analyzed 497 mpox genomes..." and Figure 2 legend " we analyzed more than 7,500 publicly available mpox clade IIb genomes...". I am confused about these more than 7,500 publicly available mpox clade IIb genomes. Please clarify and report the actual number.

The total number of sequences analyzed in our maximum likelihood phylogeny is 7,850; these can be visualized here: https://nextstrain.org/groups/blab/mpox/allcladeIIseqs . We updated line 211 to read:

“To investigate transmission dynamics into LAC, we analyzed 497 mpox genomes sampled in LAC through December 12, 2024 alongside 7,362 contextual sequences from around the world by creating a time-resolved phylogeny using Nextstrain (Fig. S2).”

And the start of Fig. 2 legend as:

“We analyzed 7,859 publicly available mpox clade IIb genomes from around the world via maximum likelihood phylogenetics using Nextstrain”

(Remarks on code availability)
